# New azolyl-derivatives as multitargeting agents against breast cancer and fungal infections: synthesis, biological evaluation and docking study

**DOI:** 10.1080/14756366.2021.1954918

**Published:** 2021-07-21

**Authors:** Cristina Maccallini, Marialucia Gallorini, Francesca Sisto, Atilla Akdemir, Alessandra Ammazzalorso, Barbara De Filippis, Marialuigia Fantacuzzi, Letizia Giampietro, Simone Carradori, Amelia Cataldi, Rosa Amoroso

**Affiliations:** a Department of Pharmacy, University “G. d’Annunzio” of Chieti -Pescara, Chieti, Italy; b Department of Biomedical, Surgical and Dental Sciences, University of Milan, Milan, Italy; c Department of Pharmacology, Faculty of Pharmacy, Bezmialem Vakif University, Computer-aided drug discovery laboratory, Istanbul, Turkey

**Keywords:** Aromatase, breast cancer

## Abstract

Nonsteroidal aromatase inhibitors (NSAIs) are well-established drugs for the therapy of breast cancer. However, they display some serious side effects, and their efficacy can be compromised by the development of chemoresistance. Previously, we have reported different indazole-based carbamates and piperidine-sulphonamides as potent aromatase inhibitors. Starting from the most promising compounds, here we have synthesised new indazole and triazole derivatives and evaluated their biological activity as potential dual agents, targeting both the aromatase and the inducible nitric oxide synthase, being this last dysregulated in breast cancer. Furthermore, selected compounds were evaluated as antiproliferative and cytotoxic agents in the MCF-7 cell line. Moreover, considering the therapeutic diversity of azole-based compounds, all the synthesized compounds were also evaluated as antifungals on different *Candida* strains. A docking study, as well as molecular dynamics simulation, were carried out to shed light on the binding mode of the most interesting compound into the different target enzymes catalytic sites.

## 1. Introduction

The aromatase enzyme (CYP19) is a haem-containing enzyme involved in the conversion of androgens into oestrogens in the last step of steroidogenesis^1^. The role of aromatase in producing higher levels of oestrogens in breast cancer (BC) cells compared to noncancerous cells^2^ has led to numerous studies on the development of inhibitors for therapeutic purposes. A variety of steroidal and nonsteroidal aromatase inhibitors (SAIs and NSAIs) has been reported^3^, and, basically, the NSAIs are better tolerated with respect to SAIs, not displaying any severe androgenic side effects^4^. However, they induce an increase in bone loss as a serious harmful effect, and therefore new NSAIs with lower drawbacks are still needed. Many of the novel NSAI molecules have azole-based structures, which are responsible for the coordination of the aromatase haem moiety, binding aromatase through noncovalent interactions in a reversible fashion^5^. Mainly, they contain imidazole, triazole and tetrazole rings, although interesting results were obtained also from some pyridyl- and indolyl-derivatives^6–8^. Compounds BAS02077837 and SYN20028567 were previously identified as promising scaffolds for the development of new AIs, showing quite potent *in vitro* activity (IC_50_ = 16.5 nM and 9.4 nM, respectively)^9^. Starting from these molecules, we have recently reported a set of imidazole- and triazole-based carbamates^10^ and imidazolyl- and indolyl-piperidine sulfonamides^11^^,^^12^ which showed interesting results. In particular, the BAS02077837-related compound **13a**^10^ and the SYN20028567 analogue **3o**^11^ were able to inhibit the aromatase with improved potency with respect to their parent compounds. In the present study, **13a** and **3o** were considered for further modifications to explore their structure-activity relationships (SARs).

**Figure 1.**
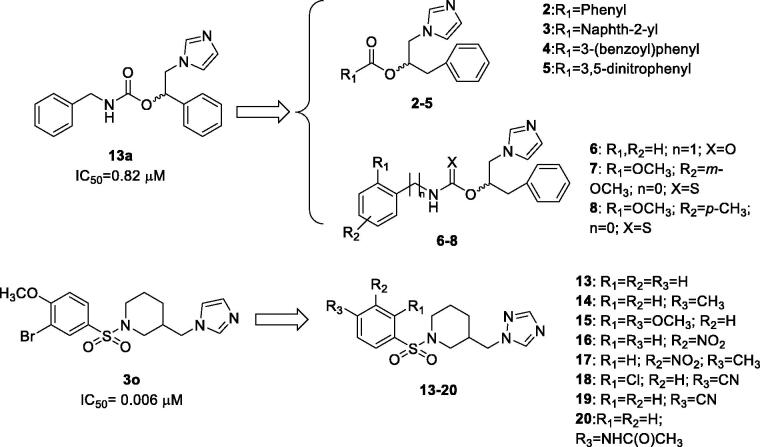
Chemical structures of the designed analogues of **13a**^9^ and **3o**^11^.

In particular, **13a** chemical scaffold was modified connecting to the stereogenic centre a benzyl group instead of the phenyl one, and different bulky aromatic moieties by means of an ester, or urethane, or a thiourethane group (Figure 1, compounds **2**–**8**). Compound **3o** was modified by introducing a triazole instead of the imidazole as the aromatase haem-coordinating moiety, and inserting different substituents on the aromatic ring, similarly to the previously reported imidazolyl-piperidine sulphonamides (Figure 1, compounds **13**–**20**). Considering the therapeutic diversity of many azole-based compounds^13^^,^^14^ and the multifactorial features of BC, it was supposed that molecules **2**–**8** and **13**–**20** could be useful to manage such a pathological condition acting as multi-target-directed ligands.

The inducible isoform of the enzyme nitric oxide synthase (iNOS) is reported to be involved in BC development^15^. It is a homodimeric protein which catalyses the biosynthesis of nitric oxide (NO) from L-arginine and is involved in the immune response, being physiologically expressed after pro-inflammatory stimuli. Nevertheless, it appears over-expressed in several pathologic conditions, including breast cancer, and its inhibition could represent a valuable tool to counteract the disease progression. Since azole-containing molecules have demonstrated activity towards this enzyme both as substrate analogues and dimerisation inhibitors^16^ and based on our ongoing efforts in the research of new molecules able to inhibit iNOS^17^^,^^18^, we evaluated compounds **2**–**8** and **13**–**20** as iNOS inhibitors, with the aim to ascertain their potential polypharmacological effects against breast cancer progression. To confirm the therapeutic potential of the most promising inhibitors of both aromatase and iNOS, they were then evaluated on a breast cancer cell line as antiproliferative and cytotoxic agents.

Further, since azole compounds are also well-established antifungal drugs able to impair membrane sterols biosynthesis in fungi by inhibiting the 14α-demethylase enzyme, we decided to select some representative compounds and evaluate them as potential antifungal agents. Indeed, chemotherapy is often associated with immune system depression, and cancer patients are at high risk of developing invasive fungal infections^19^. In particular, molecules **2**–**7** and **16**–**18** were assayed as potential anti-*Candida* agents.

Finally, a molecular docking study, as well as molecular dynamics (MD) simulations, were performed on the most promising compound (**2**, for both stereoisomers), to shed light on the binding mode with respect to two human and two fungal biological targets.

## 2. Chemistry

**Scheme 1.**
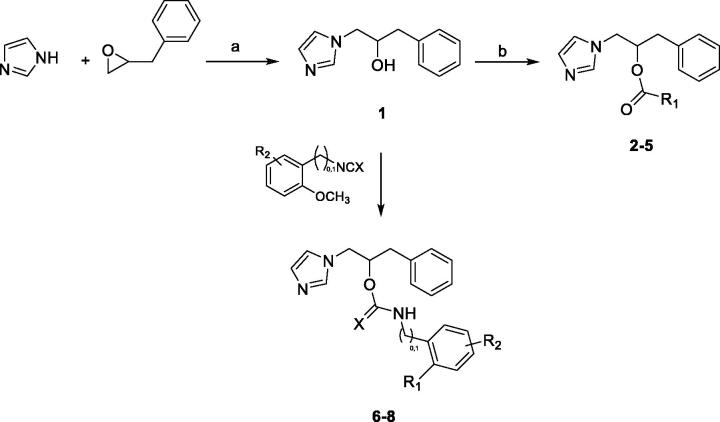
Reagents and conditions: (a) 60 °C, 12 h; (b) arylcarboxylic acid, DMAP, EDC, CH_3_CN dry, N_2_, from 0 °C to r.t., 18–22 h; (c) Et_3_N, CH_3_CN_dry_, N_2_, r.t., 18 h or NaH 60%, DMF_dry_, N_2_, from 0 °C to r.t., 20 h.

The synthesis of target molecules **2**–**8** was performed according to Scheme 1. The imidazole was reacted with (2,3-epoxypropyl)benzene to give the intermediate chiral alcohol **1**. This last was then coupled with the appropriate carboxylic acid to give molecules **2**–**5** or reacted with the appropriate benzyl isocyanate or substituted phenyl isothiocyanate to obtain molecules **6**–**8**. Standard work-up and column chromatography purification procedures were adopted to isolate in high purity each compound as a racemic mixture.

**Scheme 2.**
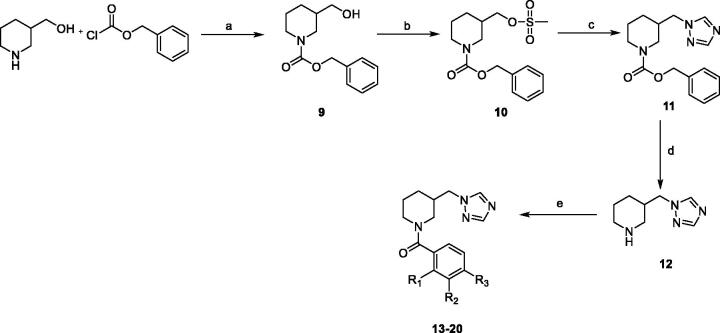
Reagents and conditions. (a) NEt_3_, DCM, from 0 °C to r.t., 24 h. (b) Mesylchloride, NEt_3_, DCM, r.t., 1 h. (c) Triazole, NaH 60% mineral oil, DMF_dry_, N_2_, 100 °C, 8 h. (d) H_2_, Pd/C, CH_3_OH_dry_, N_2_, r.t., 6 h. (e) ArSO_2_Cl, NEt_3_, DCM_dry_, N_2_, 2 h at 0 °C and 2 h at r.t.

As for molecules **13**–**20**, a synthetic route previously reported^11^ was adopted with some modifications, as represented in Scheme 2. The amino group of the 3-hydroxymethylpiperidine was protected with the benzyloxycarbonyl group, and then the obtained intermediate **9** was converted into the corresponding mesylate **10**. This last was reacted with triazole to give compound **11**, which was deprotected by means of catalytic hydrogenation. Finally, the obtained intermediate **12** was reacted with the appropriate substituted-phenyl-sulphonyl chloride to give the desired target molecules. Standard work-up and chromatographic purification procedures were used to isolate intermediates and final compounds in good yields and high purity.

## 3. Biology

### 3.1. Aromatase inhibition

Compounds **2**–**8** were evaluated as potential aromatase inhibitors by means of a fluorimetric assay kit (Aromatase-CYP19A Inhibitor Screening kit, BioVision), according to previously reported methods^10^^,^^12^. Results are listed in Tables 1–3, and they were expressed as enzyme inhibition percent, normalised to letrozole as the reference drug (positive control, 100% inhibition at 1 μM).

**Table 1.** Inhibition of aromatase and iNOS by compounds **2**–**5**.
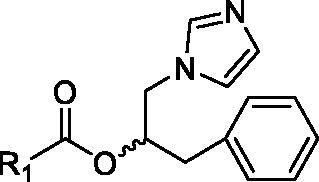

Compound	R_1_	Aromatase % inhibition	iNOS % inhibition
**2**	Phenyl	77 ± 1.8	100 ± 2.6
**3**	Naphth-2-yl	13 ± 0.5	0 ± 0.05
**4**	3-(Benzoyl)phenyl	36 ± 1.2	47 ± 1.3
**5**	3,5-dinitro-phenyl	82 ± 3.8	100 ± 1.7

Each experiment was performed in triplicate. Values are mean ± SD.

**Table 2.** Inhibition of aromatase and iNOS by compounds **6**–**8**.
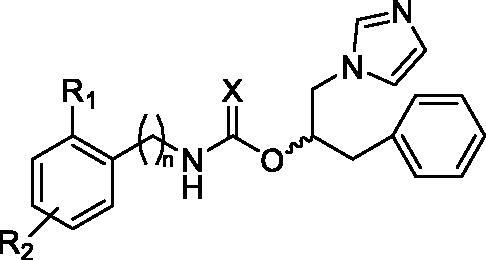

Compound	R_1_	R_2_	*n*	X	Aromatase % inhibition	iNOS % inhibition
**6**	H	H	1	O	72 ± 1.4	52 ± 0.7
**7**	OCH_3_	OCH_3_	0	S	15 ± 0.3	7 ± 0.08
**8**	OCH_3_	CH_3_	0	S	54 ± 0.9	12 ± 0.1

Each experiment was performed in triplicate. Values are mean ± SD.

**Table 3.** Inhibition of aromatase and iNOS by compounds **13**–**20**.
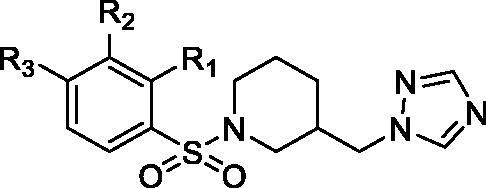

Compound	R_1_	R_2_	R_3_	Aromatase % inhibition	iNOS % inhibition
**13**	H	H	H	29 ± 1.5	27 ± 0.4
**14**	H	H	CH_3_	32 ± 1.2	22 ± 0.5
**15**	OCH_3_	H	OCH_3_	62 ± 2.8	18 ± 0.1
**16**	H	NO_2_	H	63 ± 3.1	47 ± 0.3
**17**	H	NO_2_	CH_3_	25 ± 1.3	39 ± 0.20
**18**	Cl	H	CN	55 ± 1.1	14 ± 0.20
**19**	H	H	CN	48 ± 1.7	16 ± 0.70
**20**	H	H	NHC(O)CH_3_	61 ± 2.1	0 ± 0.03

Each experiment was performed in triplicate. Values are mean ± SD.

All the evaluated molecules were from mild to strong aromatase inhibitors, confirming the usefulness of the adopted molecular scaffolds. As for analogues of **13a**, the best results were obtained from compounds **2**, **5** and **6** which gave 77, 82 and 72 enzyme percent inhibition, respectively. These data are in agreement with those observed for **13a**, which was able to affect similarly the aromatase activity, giving 81% inhibition at 1 µM. Nevertheless, compounds **3**, **4**, **7** and **8**, showing more lipophilic and hindered aromatic groups, appeared less active. Compounds **13**–**20** gave moderate aromatase inhibition, with inhibition percent values comprised between 25% and 63%. In general, with respect to the biological activity of **3o** and of the related imidazolyl-piperidin-sulphonamides derivatives of the SYN20028567 previously reported^11^, it seems that the introduction of the triazole in the molecular scaffold results in a loss of the potency of action, suggesting that in these compounds the imidazole is able to better coordinate the enzyme haem.

### 3.2. Nitric oxide synthase inhibition

Since compounds **2**–**8** and **13**–**20** bear azole moieties potentially able to inhibit the iNOS^16^ and considering the involvement of this enzyme in breast cancer progression^20^, we evaluated them as iNOS inhibitors, with the aim to ascertain their potential dual-action. The L-citrulline assay with fluorimetric detection was adopted, as previously reported^21^. The compounds were evaluated at 1 µM, and results, expressed as enzyme percent inhibition normalised to 1400 W as the reference compound (positive control, 100% inhibition at 1 μM), are reported in Tables 1–3. In general, a moderate iNOS inhibition was observed, and molecules bearing more hindered or lipophilic substituents gave a weaker or no inhibition (compounds **3**, **7**, **8**, **15**, **18**–**20**), with respect to compounds that included more simple structures or the nitro groups. Indeed, compounds **1** and **5** appeared as the most efficacious iNOS inhibitors, giving a complete enzyme inhibition. Since compounds **2**, **5** and **6** gave the most promising results both as aromatase and iNOS inhibitors, they were selected for further biological evaluations.

### 3.3. Chemical stability of 2 and 5

Compounds **2** and **5** contain an ester linkage, therefore their chemical stability was evaluated. Phosphate buffer (pH = 7.4), HCl solution (pH = 2.0), NaOH solution (pH = 9.0) were adopted as media. Immediately after dissolution and at appropriate time intervals (5′, 1 h, and 2 h), 5 µL of each solution were withdrawn and injected into an HPLC apparatus (Waters, Milford, USA), equipped with an X-Terra C8 column (Waters), eluted using a mixture of H_2_O/CH_3_CN (30:70) and revealed by a means of a photodiode array. Solutions were kept at 37 °C and monitored for 24 h. As expected, compounds **2** and **5** proved to be stable in the acidic and neutral medium, as no loss of product was observed, while they were hydrolysed in the basic medium, with a half-life of 48 min for compound **2**, and 39 min for compound **5** (the chromatogram at 254 nm is reported in Supplementary Figure S4).

### 3.4. MCF-7 proliferation and cytotoxicity evaluation

**Figure 2.**
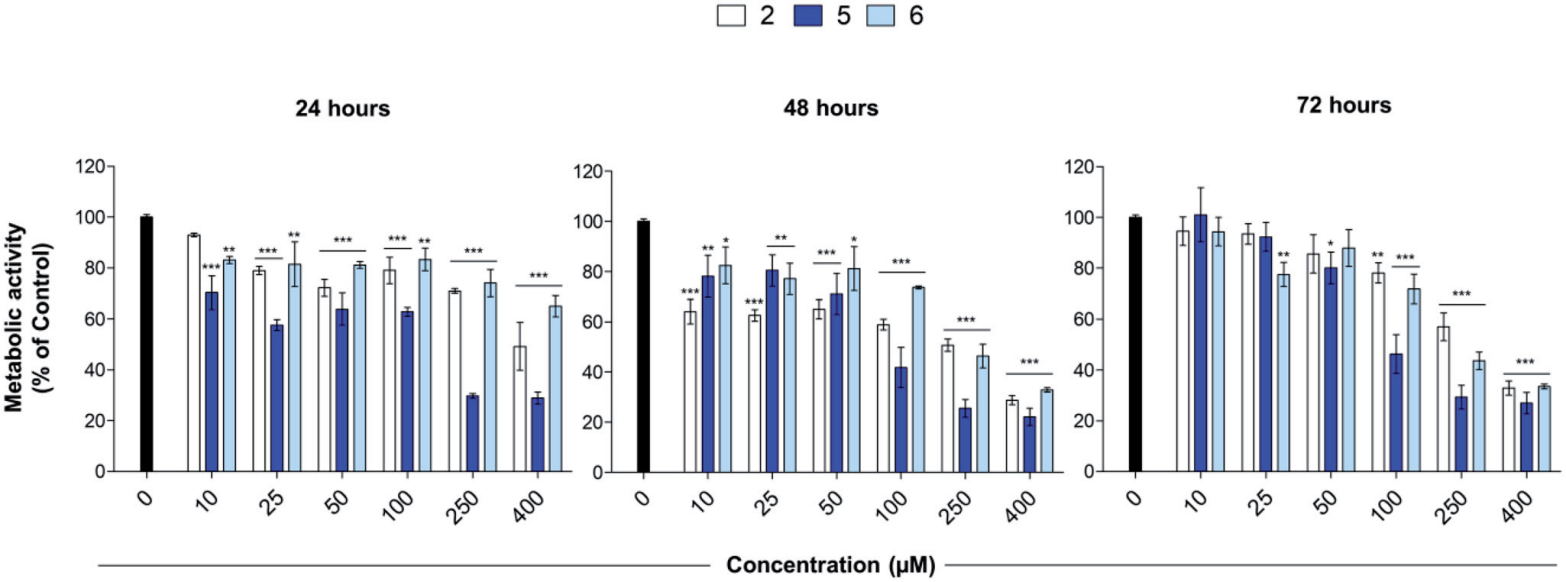
Metabolic activity of MCF-7 cells in the presence of compounds **2**, **5**, and **6** after 24, 48, and 72 h. The bar graphs show the percentage of metabolic activity of cells exposed to loading concentrations (0–400 µM) of compounds (*n* = 3). Cells in the presence of DMSO alone represent the experimental control (0 µM) set as 100%. *p < 0.01, **p < 0.001 and ***p < 0.0001 between treated samples and control (0 µM).

The antiproliferative effects of compounds **2**, **5** and **6** were evaluated on the MCF-7 breast cancer cell line by measuring their metabolic activity in response to loading concentrations of compounds up to 72 h (Figure 2). At the earliest time of exposure (24 h), a statistically significant decrease of cell metabolic activity can be detected for all the compounds tested. More in detail, the fall of the cell metabolic activity percentage calculated is clearly dose-dependent for compounds **2** and **5**. Moreover, compound **5** discloses the best IC_50_ among the series (120 µM), whereas the ones for compounds **2** and **6** are calculated over 200 and 400 µM, respectively. Likewise, after 48 h of exposure, compounds **2** and **5** reveal the best anti-proliferative activity on MCF-7 cells, being the reduction of cell metabolic activity percentages statistically significant already at the lowest concentrations tested. Furthermore, the IC_50_ calculated for compound **5** continues to decrease in a time-dependent manner, being assessed at 82.5 µM. Compound **6** is less effective than compounds **2** and **5**, starting to decrease the percentage of metabolically active cells from the dose of 100 µM. Finally, after 72 h of exposure, all the compounds here tested are less effective in the lowest concentration range (10–50 µM). Nevertheless, there is a fall in the cell metabolic activity after this concentration range for compound **5** which discloses the lowest IC_50_ comparing 24 to 48 h (68.8 µM). The higher metabolic activity registered after 72 h in the lower concentration range, as a counteraction of the decreasing trend registered after 24 and 48 h mainly for compounds **2** and **5**, could be ascribed to chemoresistance mechanisms established by MCF-7 cells^22^^,^^23^.

**Figure 3.**
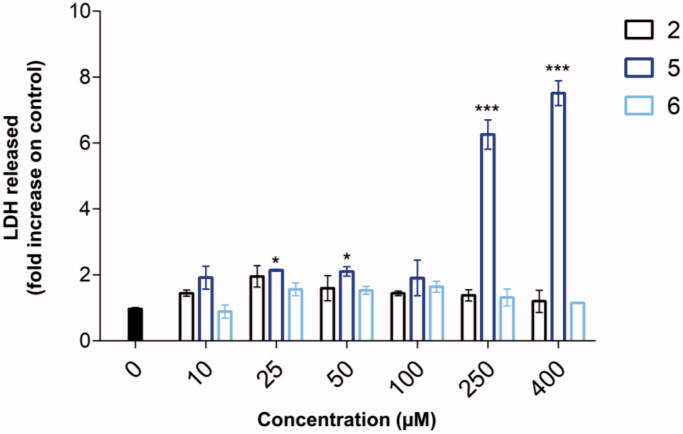
Cytotoxicity occurrence in MCF-7 cells in the presence of compounds **2**, **5**, and **6** after 24 h. The bar graph shows the LDH released from cells exposed to loading concentrations (0–400 µM) of compounds (*n* = 3) as fold increase. Cells in the presence of DMSO alone represent the experimental control (0 µM) set as 1. *p < 0.01 and ***p < 0.0001 between treated samples and control (0 µM).

In parallel, the cytotoxic effect of loading concentrations of compounds **2**, **5** and **6** on the MCF-7 cell line was evaluated after 24 h (Figure 3). As shown through the LDH assay, the decrease of the metabolic activity registered for compounds **2** and **5** can be ascribed to cytotoxicity, being the fold increases raised compared to the control sample already at the lowest concentration tested (1.44 and 1.91 folds) at 10 µM, respectively. Notably, the cytotoxicity related to compound **5** is more consistent and statistically relevant than the one exerted by compound **2**. Indeed, the LDH is released in a compound **5** dose-dependent manner, being extremely high (6.25 and 7.50 folds) after the concentration assessed as IC_50_ (120 µM).

### 3.5. Antifungal activity

Azole-based compounds are well-known antifungal agents impairing fungi membrane integrity. Typically, they are directed against the 14α-demethylase enzyme, a haem-containing oxidoreductase catalysing the lanosterol demethylation during the ergosterol biosynthesis. Since there is a need of new antifungals overcoming fungi resistance and considering the occurrence of fungi infection in oncologic and immunocompromised patients, we investigated some of the new compounds both on standard ATCC 90028 and clinical isolates of *Candida* spp. by determining their minimum inhibitory concentration (MIC). Derivatives, dissolved in dimethylsulphoxide (DMSO), were evaluated for their antifungal activity and compared with fluconazole as a reference drug. First, compounds **2**–**7** and **16**–**18**, displaying chemical features belonging to the three explored series, were chosen for an additional screening against a fluconazole-susceptible standard strain of *C. albicans* ATCC 90028 (Table 4).

**Table 4.** MIC values of the selected azoles, belonging to the three chemical scaffolds, against *C. albicans* ATCC 90028.

Compound	2	3	4	5	6	7	16	17	18	Fluconazole
MIC (µg/mL)	16	64	>64	>64	>64	>64	>64	>64	>64	0.25

The data put in evidence the lowest MIC value of compound **2**, which was further tested against 15 different clinical isolates of *Candida* spp. (Table 5).

**Table 5.** MIC values in compound **2** against a panel of 15 clinical isolates of *Candida* species.

*Candida* spp.	Compound **2**
	MIC (µg/mL)
*C. albicans* 78/5	16
*C. albicans* 1009/40	16
*C. albicans* 887/5	16
*C. albicans* 308/4	64
*C. albicans* 943/5	16
*C. albicans* 712/5	32
*C. albicans* 688/5	32
*C. albicans* 510/5	16
*C. albicans* 231/5	16
*C. albicans* SF1	16
*C. tropicalis* 74/5	64
*C. tropicalis* 295/5	64
*C. glabrata* 365/5	>64
*C. glabrata* 311/5	>64
*C. glabrata* 495/5	>64

Collectively, these data highlighted a slight preference of compound **2** for the inhibition of *C. albicans* species with respect to non-*albicans* ones (*C. tropicalis* and *C. glabrata*).

## 4. Molecular modelling studies

Considering that compound **2** was able to give anti-aromatase, iNOS inhibition and antifungal effects, it was selected for docking studies in combination with molecular dynamics simulations, in order to investigate its possible binding interactions with human aromatase and iNOS enzymes. In addition, *Candida albicans* 14α-demethylase (CaCYP51; 5v5z) and carbonic anhydrase (CaNce103; 6uwg) enzymes were investigated as possible targets to explain the antifungal effects of this compound. Both the fungal enzymes were reported to be inhibited by azole-based compounds^24^^,^^25^.

### 4.1. Investigation of possible binding interactions of compound 2 with human aromatase (hCYP19A1)

**Figure 4.**
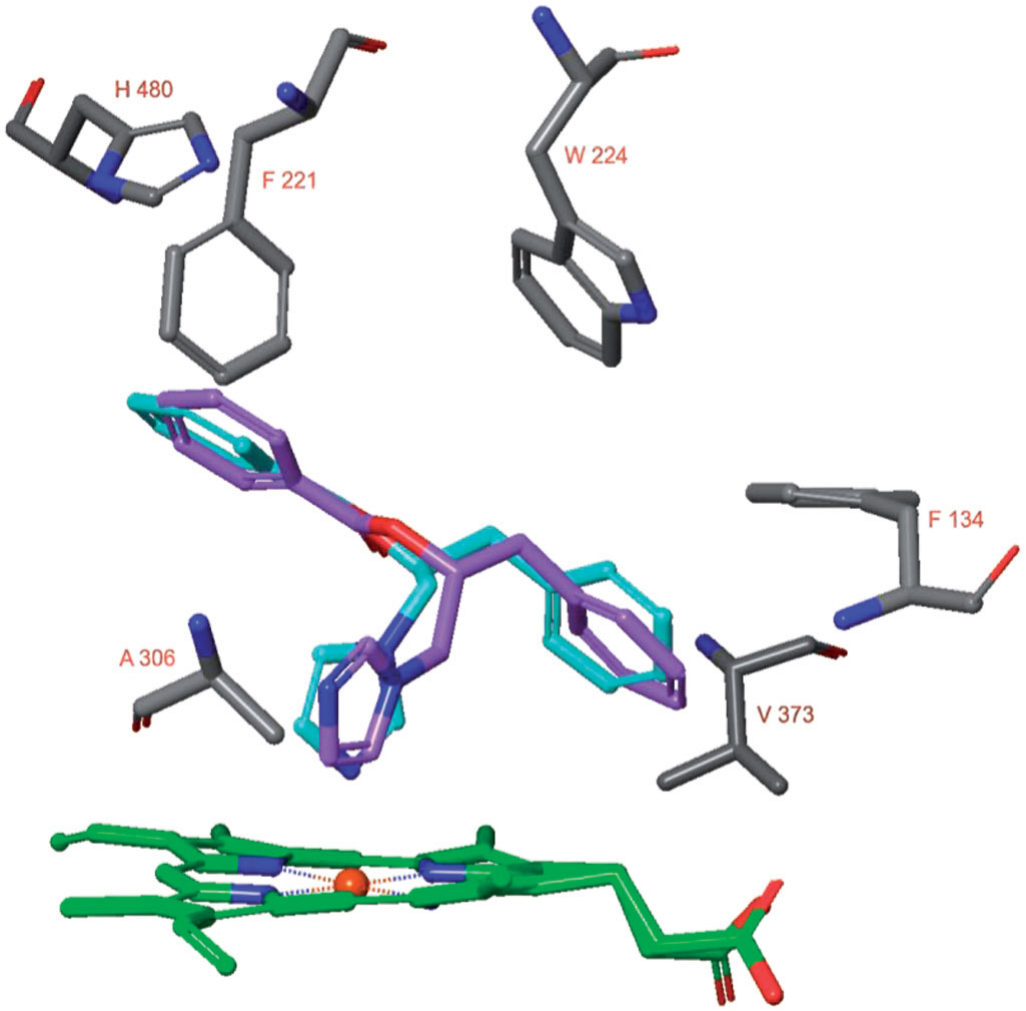
The docked poses of compounds **2(S)** (turquoise) and **2(R)** (purple) in the active site of hCYP19a1 (pdb: 3s79) are similar with the exception of the diazole nitrogen atom.

Docking studies indicated that both stereoisomers of compound **2** could adopt similar poses in the active site of aromatase (Figure 4). The diazole nitrogen atom of **2(S)** was positioned close to the haem iron atom and interactions were present. Moreover, the phenyl groups of the ligand are in proximity of Phe134, Phe221, and Trp224. Remarkably, even though the diazole moiety of compound **2(R)** was positioned similarly as observed for **2(S)**, the diazole nitrogen was not placed close to the haem iron atom. Also, no pose for **2(R)** was obtained in which this was possible. Therefore, the **2(S)** docked pose was subjected to a 50 ns MD simulation.

**Figure 5.**
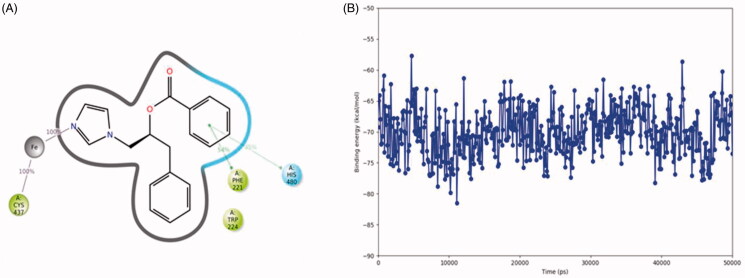
The binding interactions (A) and binding energy (B) of compound **2(S)** with the hCYP19A1 active site during the 50 ns MD simulation. Hydrophobic amino acids are indicated in green, polar amino acids are indicated in blue, aromatic hydrogen bonds and π–π stackings are indicated in green lines.

During the 50 ns MD simulation of compound **2(S)**, the interaction with the haem iron is entirely preserved (Figure 5). One of the ligand’s phenyl group forms a π–π stacking with the sidechain of Phe221 (54% of simulation time) and an aromatic interaction with the sidechain of His480 (45% of simulation time). The other phenyl group is located close to Trp224. The ligand-protein binding energy fluctuates around −70 kcal/mol (Figure 5).

hCYP19A1 in complex with the cocrystallized ligand 4-androstene-3,17-dione (pdb: 3s79) was also simulated for 50 ns according to the same protocol (Supplementary Figure S1). The binding pose of the ligand was very stable (RMSD value of ligand smaller than 1.25 Å) and the binding energy was mainly in the −60 to −55 kcal/mol range.

The stability of the docked pose of compound **2**(**S**) in the active site of hCYP19A1 and the fact that the binding energy was slightly better compared to the cocrystallized ligand (*K*_i_ = 20 nM)^26^ suggest that compound **2**(**S**) may strongly bind to the active site of hCYP19A1 in the suggested pose.

### 4.2. Investigation of possible binding interactions of compounds 2 with iNOS

**Figure 6.**
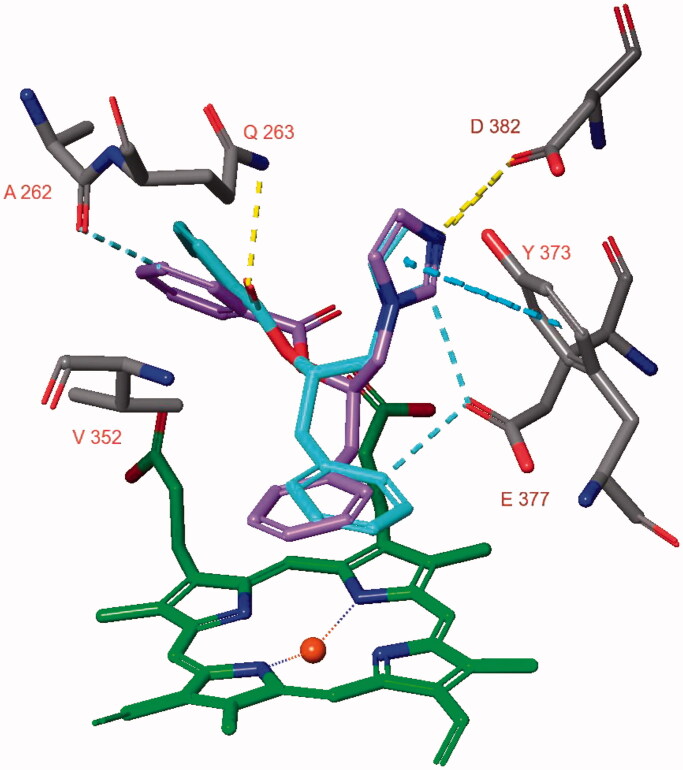
The docked pose of compounds **2(S)** (turquoise) and **2(R)** (purple) in the active site of human iNOS (pdb: 4nos).

The docked poses of compounds **2(S)** and **2(R)** in the iNOS active site are very similar (Figure 6). The ligand’s phenyl group is positioned almost parallel to the haem group and π–π stacking with the haem group is present. The diazole ring forms π–π stacking with Tyr373 and an aromatic hydrogen bond with the sidechain of Glu377. This diazole nitrogen forms a hydrogen bond and electrostatic interactions with the sidechain of Asp382. The other ligand phenyl ring is located close to Ala262. Compound **2(R)** forms an aromatic hydrogen bond with the backbone carbonyl group of Ala262, while the carbonyl group of compound **2(S)** forms a hydrogen bond with the sidechain of Gln263. As the hydrogen bond with Gln263 is expected to be stronger compared to the aromatic hydrogen bond with Ala262, the docked pose of compound **2(S)** was selected for a 50 ns MD simulation.

**Figure 7.**
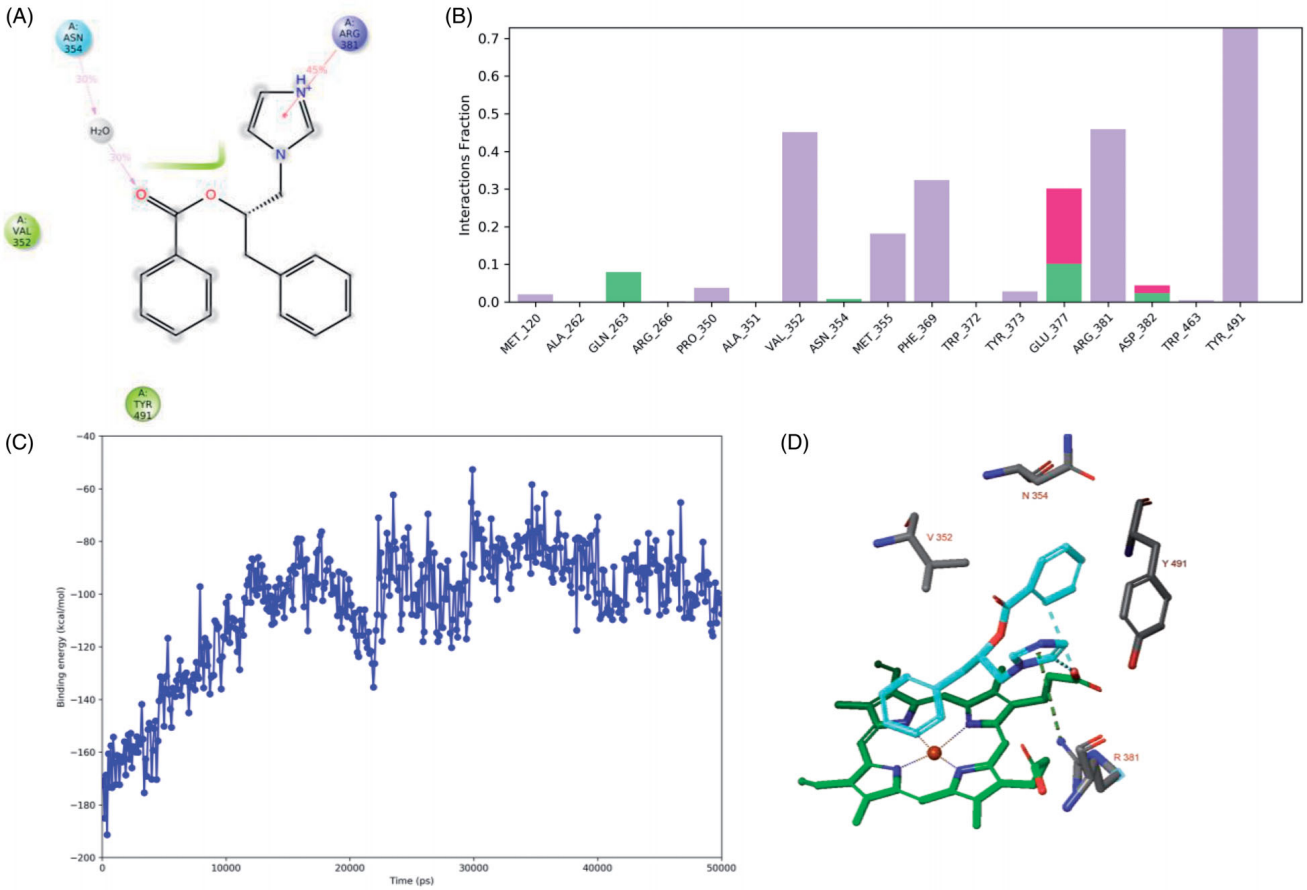
The binding interactions (panels A and B) and binding energy (panel C) of compound **2**(**S**) with the iNOS active site during the 50 ns MD simulation. The binding pose was obtained after 50 ns MD simulation (panel D). Hydrophobic amino acids are indicated in green, polar amino acids are indicated in blue, cationic amino acids are indicated in purple. Hydrogen bonds are indicated in green bars, hydrophobic interactions are indicated in purple bars and ionic interactions are indicated in red bars.

The 50 ns MD simulation shows that the hydrogen bond and electrostatic interactions between the diazole nitrogen and the sidechain of Asp382 and the hydrogen bond between the ligand’s carbonyl group with the sidechain of Gln263 are not stable (Figure 7). Instead, the ligand forms cation–π interactions with between its diazole group and the sidechain of Arg381. Interaction via a bridging water molecule occurs between the ligand’s carbonyl group and Asn354. Hydrogen bonds are occasionally formed with Gln263, Glu377 and Asp382 (≤ 10% of simulation time). In addition, ionic interactions also occur occasionally with Glu377 and Asp382 (≤ 20% of simulation time). The calculated binding energy increases from approximately −180 kcal/mol and to approximately −100 kcal/mol during the simulation. This indicates a diminishing strength of binding for the ligand.

The co-crystal structure of iNOS in complex with ethylisothiourea (*K*_i_= 5 nM)^26^ was simulated using the same protocol (Supplementary Figure S2). The ligand mainly formed interactions with Trp372 (38% of simulation time) and Glu377 (84% of simulation time). The binding energy increased during the first 25 ns from 150 kcal/mol to approximately −110 kcal/mol and later is decreased towards approximately −170 kcal/mol. The binding pose after 50 ns of MD simulation is close to the docked pose, but several important changes have been observed. The imidazole moiety does not form an interaction with Glu377, but instead forms hydrophobic interactions and aromatic hydrogen bonds with the haem group (Figure 7(D)). In addition, a cation-π interaction is present between the sidechain of Arg381 and the imidazole group of the ligand.

### 4.3. Investigation of fungal CaCYP51 as possible target for compound 2

**Figure 8.**
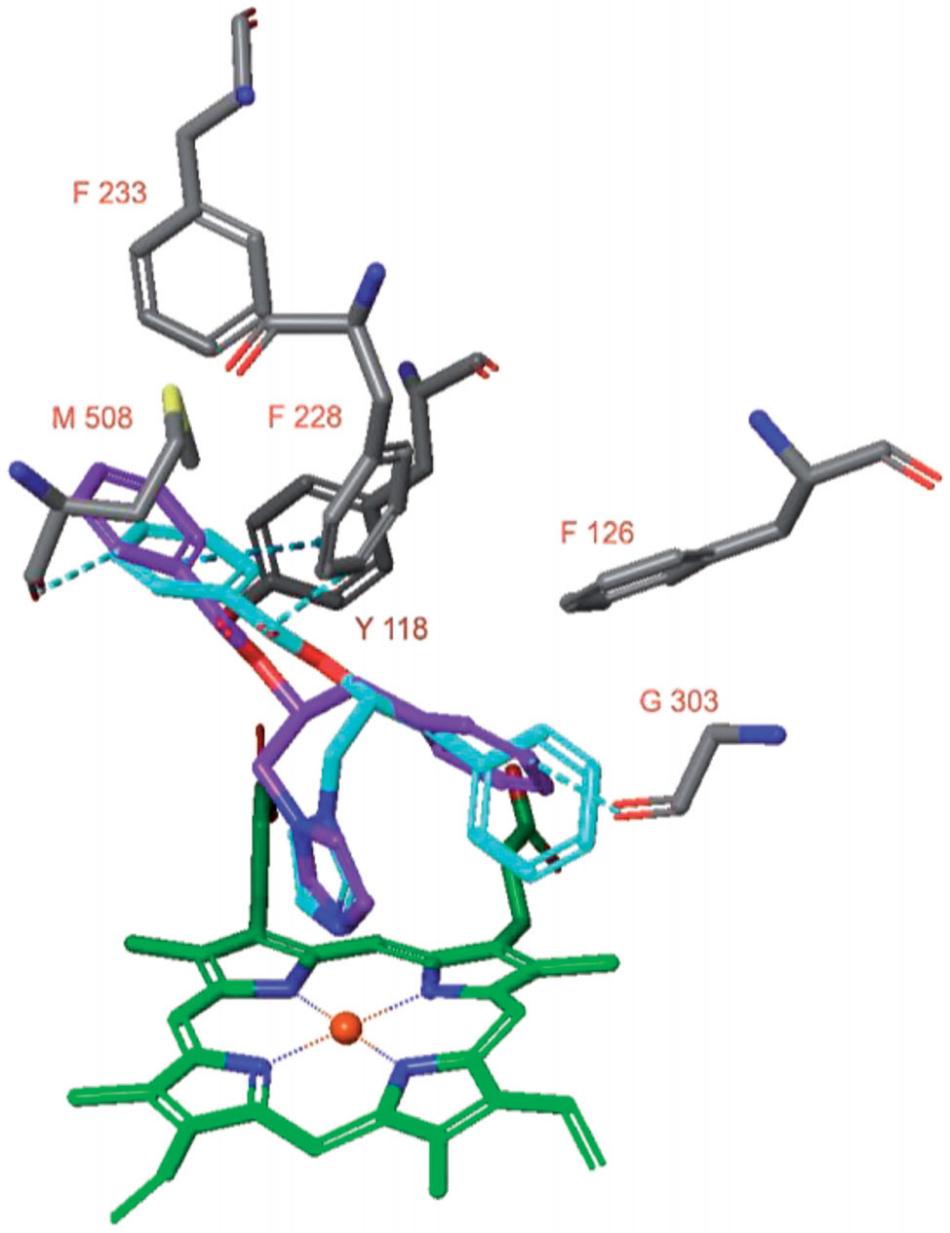
The docked pose of compound **2** (R stereoisomer in turquoise, S stereoisomer in purple) in the active site of CaCYP51 (pdb: 5v5z).

The docked poses of both stereoisomers of compound **2** in the active site of CaCYP51 (pdb: 5v5z) are very similar (Figure 8). The diazole nitrogen atom is within interaction distance to the haem iron atom and the phenyl group next to the carbonyl group is located between Tyr118 and Met508. Aromatic hydrogen bonds are formed between the ligand and the sidechain of Phe228 or carbonyl backbone of Met508. The other phenyl group is located near Phe126 and Gly303, and an aromatic hydrogen bond is formed with the latter.

**Figure 9.**
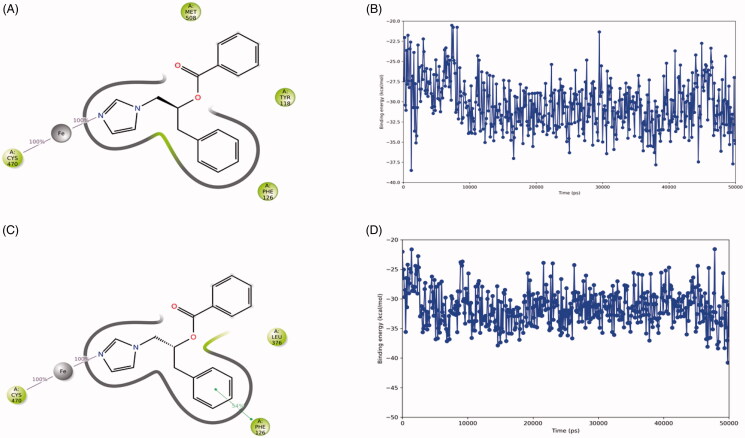
The binding interactions (panel A) and binding energy (panel B) of compound **2(R)** as well as the binding interactions (panel C) and binding energy (panel D) of compound **2(S)** with the CaCYP51 active site during the 50 ns MD simulation. Hydrophobic amino acids are indicated in green, polar amino acids are indicated in blue, aromatic hydrogen bonds and π–π stackings are indicated in green lines.

CaCYP51 in complex with itraconazole was simulated for 50 ns and the ligand formed an interaction between its triazole nitrogen atom and the haem iron atom during the entire simulation (Supplementary Figure S3). In addition, the phenyl groups formed π–π stackings with Tyr64 for 58% of the simulation time. The binding energy decreased during the simulation towards approximately −70/-80 kcal/mol. This suggests that the stereoisomers of compound **2** may bind with moderate affinity to CaCYP51 (Figure 9).

### 4.4. Investigation of fungal CaNce103 as possible target for compound 2

**Figure 10.**
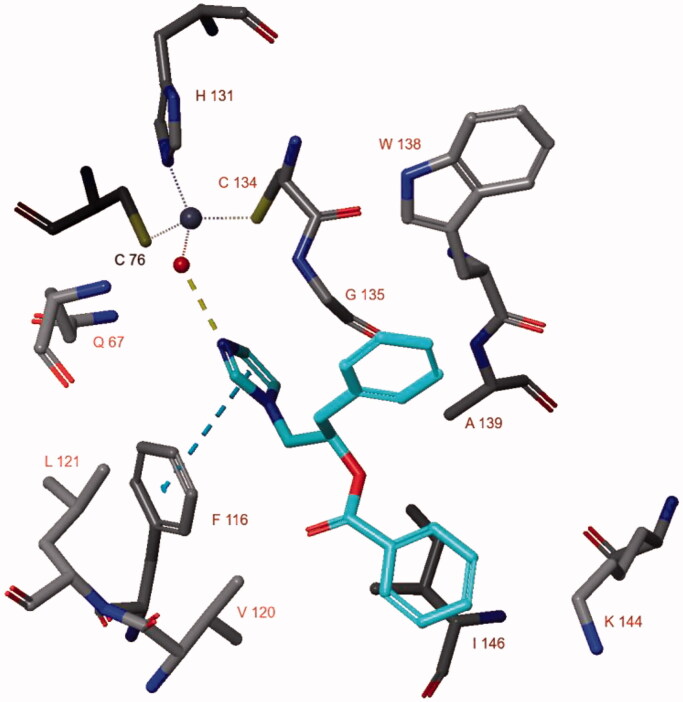
The docked pose of compound **2(R)** in the active site of CaNce103 (pdb: 5v5z).

Docking studies for CaNce103 indicated that only compound **2(R)** can form a hydrogen bond with the zinc-bound water molecule (Figure 10). Phe116 is involved in both hydrophobic interactions as well as an aromatic hydrogen bond with the ligand’s carbonyl group. Additional hydrophobic interactions were present with Trp138 and Ile146.

**Figure 11.**
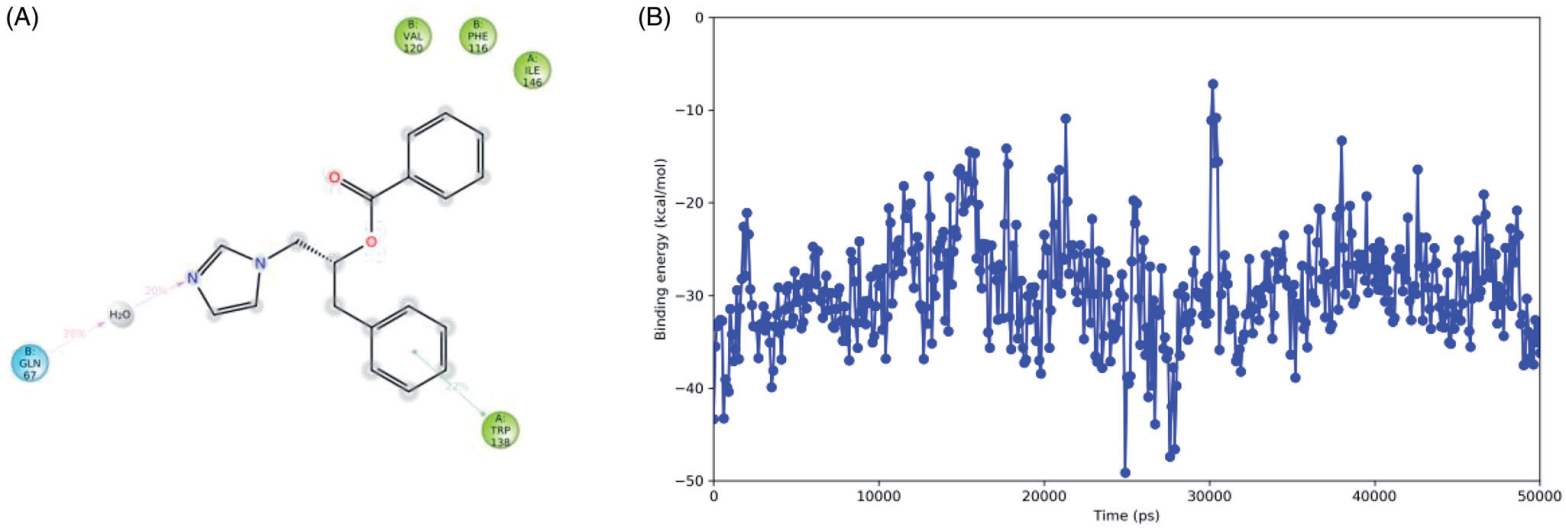
The binding interactions (panel A) and binding energy (panel B) of compound **2**(**S**) with the CaNce103 active site during the 50 ns MD simulation. Hydrophobic amino acids are indicated in green and polar amino acids are indicated in blue.

The docked pose of **2**(**S**) in the CaNce103 active site has been simulated for 50 ns and the interaction of the imidazole nitrogen atom with the Zn^2+^-bound water molecule is lost early in the simulation (Figure 11). Instead, the ligand moves outward and occasionally forms a water-bridged hydrogen bond with Gln67 (20% of the simulation time). In addition, one of the ligand’s phenyl groups forms π–π stacking with Trp138 (22% of the time). The binding energy during the simulation fluctuates around −40/–30 kcal/mol.

Due to the limited interactions registered in silico and the moderate binding affinities, specific assays against these fungal enzymes were not further performed.

## 5. Conclusions

The research of new therapeutic agents for the treatment of breast cancer often involves the evaluation of multitargeting compounds. The present study has disclosed some new imidazolyl- and triazolyl-derivatives which were evaluated as anti-aromatase and iNOS inhibitors, given the important role of these two enzymes in breast cancer development. Compounds **2**, **5**, and **6** were endowed with balanced dual activity against these two enzymes, which were almost completely inhibited at 1 µM. Interestingly, **2**, **5**, and **6** confirmed their potential therapeutic usefulness resulting in antiproliferative effects on MCF-7 breast cancer cell line after 48 h treatment, and compound **5** gave also cytotoxic effects after 24 h treatment. Moreover, based on the therapeutic diversity of azole-based compounds, selected molecules were subjected to further biological evaluations as anti-*Candida* agents, and **2** gave interesting results both on standard ATCC 90028 and clinical isolates of *Candida* spp. Therefore, this compound emerged as a potential multitherapeutic agent and the performed docking study allows to shed light on its binding poses into the four different target enzymes considered in the present study, confirming the obtained biological results. Collectively, the obtained data could be helpful in the research of new multitargeting and multitherapeutic agents.

## 6. Experimental protocols

### 6.1. Chemistry

#### 6.1.1. General methods and materials

All chemicals were purchased from commercial sources and used without further purification. Flash chromatography was performed on silica gel 60 (Merck) and TLC on silica gel 60, F254. Melting points were determined on a Buchi apparatus and given uncorrected. NMR spectra were run on a Varian instrument, operating at 300 (^1^H) or 75 (^13^C) MHz; chemical shifts (*δ*) are reported in ppm. HPLC analyses were performed using a Waters (Milford, MA, USA) system composed of a P600 model pump, a 2996 photodiode array detector, and a 7725i model sample injector (Rheodyne, Cotati, CA, USA). Chromatograms were recorded on a Fujitsu Siemens Esprimo computer and the Empower Pro software (Waters) processed data. The analyses were performed on an XTerra MS C8 column (250 × 4.6 mm i.d., 5 µm particle size) (Waters), equipped with an XTerra MS C8 guard column (Waters). A column thermostat oven module Igloo-Cil (Cil Cluzeau Info Labo, France) was used. To evaluate target compounds’ purity and chemical stability, the column was eluted at a flow rate of 1 ml/min with a mixture of ultrapure H_2_O and CH_3_CN (30:70). All tested compounds had a purity of ≥95%. Elemental analyses were carried out by the Eurovector Euro EA 3000 model analyser. Analyses indicated by the symbols of the elements were within ±0.4% of the theoretical values. For the evaluation of iNOS inhibition, the HPLC column was eluted at a flow rate of 0.7 ml/min with linear gradients of buffers A (5% CH_3_CN in 15 mM sodium borate with 0.1% v/v trifluoroacetic acid, pH 9.4) and B (50% CH_3_CN in 8 mM sodium borate with 0.1% v/v trifluoroacetic acid, pH 9.4). The solvent gradient was 0–20% B at 0–10 min, B to 25% at 10–15 min, then to 40% at 15–20 min and to 70% at 20–28 min. This composition was maintained until *t* = 35 min, before being reduced to the initial 0% B composition. The injection volume was 5 μL. The fluorescence intensity in the column eluate was monitored at 335 nm (excitation) and 439 nm (emission).

#### 6.1.2. Synthesis

##### 6.1.2.1. 1-(1*H*-imidazol-1-yl)-3-phenylpropan-2-ol (1)

Imidazole (0.0045 mol) was added to 2,3-epoxystirene (0.0045 mol) and the mixture was allowed to react under magnetic stirring at 60 °C for 12 h. Then the crude compound was purified by means of silica gel chromatography, using a mixture 8:2 of ethyl acetate and methanol as the mobile phase. Colourless oil, 85% yield. ^1^HNMR (CDCl_3_): *δ* 2.77 (dd, *J* = 5.9, 13.7 Hz, 2H, CH_2_); 3.82 (dd, *J* = 7.5, 13.8 Hz, 2H, CH_2_); 3.96 (d, *J* = 3.0 Hz, 1H, OH); 4.01–4.05 (m, 1H, CH); 6.87–7.34 (m, 8H, CHar).^13^CNMR (CDCl_3_): 40.5, 50.1, 72.2, 122.4, 127.1, 128.5, 132.2, 137.7, 138.5.

#### 6.1.3. General procedure for the synthesis of 2–5

DMAP (1.5 mmol) and EDC (1.5 mmol) were added to a solution of the appropriate carboxylic acid (1.5 mmol) in CH_3_CN dry (5 ml) at 0 °C, under N_2_, under magnetic stirring. After 15 min, a solution of compound **1** (1.5 mmol) in CH_3_CN dry (1 ml) was added dropwise under the same conditions. The mixture was allowed to react for 20–24 h at r.t., under stirring and N_2_ and then it was concentrated to dryness. The obtained residue was dissolved in dichloromethane and washed with NaHCO_3ss_ (3 × 5 ml) and brine (2 × 5 ml). The combined organic layers were dried over anhydrous Na_2_SO_4_ and concentrated to reduced pressure. The crude compound was purified by means of silica gel chromatography, using a mixture 95:5 of ethyl acetate and methanol as the mobile phase.

##### 6.1.3.1. 1-benzyl-2-(1*H*-imidazol-1-yl)ethyl benzoate (2)

White solid, 75% yield. M.p.: 68–69 °C. ^1^HNMR (CD_3_OD): *δ* 2.94–3.09 (m, 2H, CH_2_); 4.26–4.44 (m, 2H, CH_2_); 5.55–5.63 (m, 1H, CH); 6.90 (s, 1H, CHar); 7.11 (s, 1H, CHar); 7.19–7.23 (m, 1H, CHar); 7.24 (d, *J* = 4.5 Hz, 4H, CHar); 7.41 (*t*, *J* = 8.7, 15.3 Hz, 2H, CHar); 7.56–7.63 (m, 2H, CHar); 7.89 (d, *J* = 7.2 Hz, 2H, CHar). ^13^CNMR (CD_3_OD): 37.4, 41.2, 49.2, 73.8, 120.0, 126.5, 127.6, 128.1, 128.2, 129.0, 129.1, 129.4, 133.0, 136.3, 137.7, 165.3. Anal calcd for C_19_H_18_N_2_O_2_: C, 74.49; H, 5.92; N, 9.14. Found C, 74.54; H, 5.90; N, 9.15.

##### 6.1.3.2. 1-benzyl-2-(1*H*-imidazol-1-yl)ethyl 2-naphthoate (3)

White solid, 70% yield. M.p.: 71–72 °C. ^1^HNMR (CD_3_OD): *δ* 2.68 (ddd, *J* = 6.6 Hz, 2H, CH_2_); 3.91–4.07 (m, 2H, CH_2_); 5.27–5.35 (m, 1H, CH); 6.69 (s, 1H, CHar); 6.95 (s, 1H, CHar); 7.05–7.08 (m, 2H, CHar); 7.19–7.33 (m, 6H, CHar); 7.46–7.50 (m, 2H, CHar); 7.64 (s, 1H, CHar); 7.79 (m, 2H, CHar). ^13^CNMR (CD_3_OD): 37.5, 41.6, 48.7, 73.5, 119.5, 126.0, 126.3, 127.0, 127.2, 127.6, 128.1, 128.4, 128.7, 129.2, 129.5, 130.7, 132.5, 133.4, 135.5, 137.7, 170.5. Anal calcd for C_23_H_20_N_2_O_2_: C, 77.51; H, 5.66; N, 7.86. Found C, 77.58; H, 5.68; N, 7.84.

##### 6.1.3.3. 1-benzyl-2-(1*H*-imidazol-1-yl)ethyl 3-benzoylbenzoate (4)

White solid, 74% yield. M.p.: 86–87 °C. ^1^HNMR (CDCl_3_): *δ* 2.85 (ddd, *J* = 6.6 Hz, 2H, CH_2_); 4.08–4.26 (m, 2H, CH_2_); 5.52 (oct , *J* = 4.2 Hz, 1H, CH); 6.91 (s, 1H, CHar); 7.03 (s, 1H, CHar); 7.20–7.33 (m, 5H, CHar); 7.43 (s, 1H, CHar); 7.50–7.67 (m, 4H, CHar); 7.80 (d, *J* = 8.1 Hz, 2H, CHar); 8.01 (d, *J* = 8.1 Hz, 1H, CHar); 8.16 (d, *J* = 8.1 Hz, 1H, CHar); 8.38 (s, 1H, CHar). ^13^CNMR (CDCl_3_): 37.7, 49.0, 74.1, 119.7, 127.2, 128.5, 128.8, 129.3, 129.6, 130.0, 130.9, 132.9, 133.1, 134.6, 135.4, 136.8, 137.6, 138.0, 164.7. Anal calcd for C_26_H_22_N_2_O_3_: C, 76.08; H, 5.40; N, 6.82. Found C, 76.12; H, 5.42; N, 6.81.

##### 6.1.3.4. 1-benzyl-2-(1*H*-imidazol-1-yl)ethyl 3,5-dinitrobenzoate (5)

Yellow solid, 67% yield. M.p.: 144–145 °C. ^1^HNMR (CDCl_3_): *δ* 2.99 (ddd, *J* = 6.9, 7.2 Hz, 2H, CH_2_); 4.20–4.35 (m, 2H, CH_2_); 5.61 (oct, *J* = 4.2 Hz, 1H, CH); 6.92 (s, 1H, CHar); 7.05 (s, 1H, CHar); 7.23–7.37 (m, 5H, CHAr); 7.45 (s, 1H, CHar); 9.02 (d, *J* = 2.1 Hz, 2H, CHar); 9.22 (*t*, *J* = 2.1 Hz, 4.2 Hz, 1H, CHar). ^13^CNMR (CDCl_3_): 38.4, 49.4, 76.2, 117.5, 119.8, 123.2, 128.0, 129.5, 129.7, 130.6, 133.4, 135.3, 138.1, 149.1, 162.0. Anal calcd for C_19_H_16_N_4_O_6_: C, 57.58; H, 4.07; N, 14.14. Found C, 57.61; H, 4.06; N, 14.16.

#### 6.1.4. General procedure for the synthesis of 6–8

NaH (60% mineral oil, 0.68 mmol) was added to a solution of compound **1** (0.68 mmol) in dry DMF (2 ml) at r.t., under stirring and N_2_. After 15 min, the mixture was cooled at 0 °C, and the appropriate equimolar benzylisocyanate, or 2,4-dimethoxy-phenyl-thioisocyanate, or 2-methoxy-5-methylisothiocyanate was added. After 20 h, reactions were stopped and the solvent was evaporated off. The crude residue was treated with aqueous ammonium chloride (0.125 g dissolved in 5 ml of water), and the obtained mixture was extracted with diethyl ether (2 × 4 ml). The combined organic layers were then washed with water (5 ml), dried over anhydrous Na_2_SO_4_, and concentrated at reduced pressure. The crude compounds were finally purified by means of silica gel chromatography, using a mixture 98:2 of ethyl acetate and methanol as the mobile phase.

##### 6.1.4.1. 1-benzyl-2-(1*H*-imidazol-1-yl)ethyl benzylcarbamate (6)

Yellow oil, 42% yield. ^1^HNMR (CDCl_3_): *δ* 2.69 (ddd, *J* = 6.9 Hz, 7.2 Hz, 2H, CH_2_); 3.93–4.15 (m, 2H, CH_2_); 4.29 (dd, *J* = 3.6 Hz, 5.7 Hz, 2H, CH_2_); 5.23 (m, 1H, CH); 5.42 (*t*, *J* = 6.3, 11.7 Hz, 1H, NH); 6.88 (s, 1H, CHar); 7.03 (s, 1H, CHar); 7.17–7.34 (m, 10H, CHAr); 7.44 (s, 1H, CHar). ^13^CNMR (CDCl_3_): 37.7, 44.9, 48.9, 73.5, 119.8, 127.0, 127.4, 127.5, 128.7, 129.2, 129.3, 129.5, 135.9, 137.9, 138.1, 155.4. Anal calcd for C_20_H_21_N_3_O_2_: C, 71.62; H, 6.31; N, 12.53. Found C, 71.66; H, 6.29; N, 12.55.

##### 6.1.4.2. *O*-[1-benzyl-2-(1*H*-imidazol-1-yl)ethyl] (2,4-dimethoxyphenyl)thiocarbamate (7)

Yellow oil, 35% yield. ^1^HNMR (CDCl_3_): *δ* 2.82 (ddd, *J* = 6.6, 7.2 Hz, 2H, CH_2_); 3.72 (s, 3H, CH_3_); 3.76 (s, 3H, CH_3_); 4.05 (dd, *J* = 5.1, 15 Hz, 1H, CHH); 4.47 (dd, *J* = 3.9, 14.7 Hz, 1H, CHH); 5.29 (s, 1H, NH); 5.60 (m, 1H, CH); 6.39 (dd, *J* = 2.7, 8.1 Hz, 1H, CHar); 6.47 (d, *J* = 2.7 Hz, 1H, CHar); 6.96 (s, 1H, CHar); 7.05 (s, 1H, CHar); 7.19–7.30 (m, 6H, CHar); 7.48 (s, 1H, CHar). ^13^CNMR (CDCl_3_): 37.1, 47.7, 55.5, 55.6, 71.8, 99.7, 103.9, 120.0, 122.0, 126.9, 127.0, 128.4, 128.8, 129.3, 129.4, 135.9, 137.9, 151.4, 155.6, 157.6. Anal calcd for C_21_H_23_N_3_O_3_S: C, 63.45; H, 5.83; N, 10.57. Found C, 63.48; H, 5.84; N, 10.56.

##### 6.1.4.3. *O*-[1-benzyl-2-(1*H*-imidazol-1-yl)ethyl] (2-methoxy-4-methylphenyl)thiocarbamate (8)

White solid, 61% yield. M.p. 120–121 °C. ^1^HNMR (CDCl_3_): *δ* 2.25 (s, 3H, CH_3_); 2.79 (ddd, *J* = 5.1, 7.5 Hz, 2H, CH_2_); 3.83 (s, 3H, CH_3_); 4.07 (m, 2H, CH_2_), 4.25 (dd, *J* = 4.2, 15.0 Hz, 1H, CH) 6.04 (bs, 1H, NH); 6.77 (d, *J* = 8.1 Hz, 1H, CHar); 6.92–7.07 (m, 3H, CHar); 7.23–7.34 (m, 7H, CHAr). ^13^CNMR (CDCl_3_): 20.8, 36.9, 48.1, 55.8, 76.6, 110.0, 119.8, 126.4, 127.2, 128.8, 129.3, 129.6, 130.1, 135.5, 137.9, 152.5. Anal calcd for C_21_H_23_N_3_O_2_S: C, 66.12; H, 6.08; N, 11.01. Found C, 66.17; H, 6.09; N, 11.00.

##### 6.1.4.4. benzyl 3-(hydroxymethyl)piperidine-1-carboxylate (9)

A suspension of 3-hydroxymethyl-piperidine (10 mmol) in CH_2_Cl_2_ (20 ml) was cooled at 0 °C and then triethylamine (10 mmol) and benzylchloroformate (10 mmol) were added dropwise. The mixture was allowed to warm at room temperature and reacted under stirring for 24 h. Then, it was diluted with dichloromethane (80 ml) and washed with HCl 5% (2 × 50 ml), NaHCO_3ss_ (2 × 50 ml) and finally with brine (2 × 50 ml). The organic layer was dried over anhydrous Na_2_SO_4_, and the solvent was evaporated off to give the desired product as a pale yellow oil (75% yield). ^1^HNMR (CD_3_OD): *δ* 1.22–1.51 (m, 2H, CH_2_); 1.79–2.61 (m, 4H, CH_2_); 2.64 (d, *J* = 9.6 Hz, 1H, CH); 2.85 (d, *J* = 10.5 Hz, 1H, CH); 3.35–3.37 (m, 1H, CH); 3.43–3.98 (m, 2H, CH); 3.98 (dd, *J* = 7.7, 13.5 Hz, 2H, CH_2_OH); 5.09 (s, 2H, CH_2_); 7.28–7.38 (m, 5H, CHar). ^13^CNMR (CD_3_OD): 24.0, 27.7, 37.0, 43.5, 45.7, 65.7, 67.0, 127.4, 127.9, 128.1, 137.0, 156.4.

##### 6.1.4.5. benzyl 3-{[(methylsulfonyl)oxy]methyl}piperidine-1-carboxylate (10)

Compound **9** (6.32 mmol) was dissolved in CH_2_Cl_2_ (12 ml) and then triethylamine (6.32 mmol) and mesylchloride (1.05 mmol) were added dropwise at 0 °C, under stirring. The mixture was then warmed at room temperature and allowed to react for 30 min. The obtained precipitate was filtered off, and the filtrate was washed with NaHCO_3ss_ (2 × 10 ml), and NaCl_ss_ (2 × 10 ml). The organic layer was dried over Na_2_SO_4_, and the solvent was evaporated off to give the desired product as a colourless oil (94% yield). ^1^HNMR (CD_3_OD): *δ* 1.26–1.55 (m, 2H, CH_2_); 1.62–1.71 (m, 1H, CHH); 1.77–1.97 (m, 2H, CH_2_); 2.78–2.96 (m, 2H, CH_2_); 3.09 (s, 3H, CH_3_); 3.81–3.94 (m, 1H, CHH), 4.15–4.20 (m, 3H, CH + CH_2_); 5.10 (s, 2H, CH_2_); 7.28–7.41 (m, 5H, CHar); ^13^CNMR (CD_3_OD): 21.7, 31.3, 37.5, 42.9, 47.6, 61.2. 67.9, 75.2, 75.9, 127.9, 128.4, 128.8, 137.9, 155.2.

##### 6.1.4.6. benzyl 3-(1*H*-1,2,4-triazol-1-ylmethyl)piperidine-1-carboxylate (11)

Triazole (4.24 mmol) was suspended in dry DMF (2 ml) and NaH (60% mineral oil, 5.51 mmol) was added under N_2_, under stirring. After 30 min, a solution of intermediate **10** (4.24 mmol) in dry DMF (13 ml) was added dropwise, under N_2_, under stirring. The mixture was warmed at 100 °C, and allowed to react for 24 h. Then it was filtered, and concentrated at reduced pressure. The obtained residue was partitioned between water (20 ml) and ethyl acetate (20 ml), and the aqueous layer was further extracted with ethyl acetate (2 × 20 ml). The combined organic phases were washed with brine (2 × 25 ml), dried over anhydrous Na_2_SO_4,_ and concentrated *in vacuo* to give the crude product, which was purified by means of silica gel chromatography, using a mixture 9:1 of dichloromethane and methanol as the mobile phase. Colourless oil, 65% yield. ^1^HNMR (CDCl_3_): *δ* 1.20–1.29 (m, 1H, CH); 1.43–1.57 (m, 1H, CH); 1.62–1.72 (m, 2H, CH_2_); 2.05–2.18 (m, 1H, CH); 2.81 (q, *J* = 9.6, 12.9 Hz, 1H, CH); 2.91–3.10 (m, 1H, CH); 3.81–3.89 (m, 2H, CH_2_); 3.97–4.18 (m, 2H, CH_2_); 5.07 (s, 2H, CH_2_); 7.32–7.47 (m, 5H, CHar); 7.98 (s, 1H, CHar); 8.43 (s, 1H, CHar). ^13^CNMR (CDCl_3_): 27.8, 30.9, 36.0, 44.4, 46.8, 51.8, 67.1, 127.9, 128.0, 128.5, 138.2, 143.5, 152.1.

##### 6.1.4.7. 3-(1*H*-1,2,4-triazol-1-ylmethyl)piperidine (12)

Intermediate **11** (3.89 mmol) was dissolved in dry methanol (15 ml), and Pd/C (3.89 mmol) was added under N_2_, at room temperature, under stirring. Then H_2_ was bubbled for 6 h, after which the catalyst was filtered off. The solvent was concentrated at reduced pressure obtaining the desired intermediate **12** as a pale yellow oil (97% yield). ^1^HNMR (CDCl_3_): *δ* 1.18–1.29 (m, 1H, CHH); 1.79–1.89 (m, 3H, CH_2_+CHH); 2.35–2.42 (m, 1H, CHH); 2.50 (*t*, *J* = 12.3, 23.1 Hz, 1H, CH); 2.73–2.82 (m, 1H, CHH); 3.12 (d, *J* = 12.3 Hz, 1H, CHH); 3.25 (d, *J* = 12.3 Hz, 1H, CHH); 3.86 (ddd, *J* = 6.6, 7.8 Hz, 2H, CH_2_); 6.10 (bs, 1H; NH); 6.92 (s, 1H, CHar); 7.15 (s, 1H, CHar); 7.64 (s, 1H, CHar). ^13^CNMR (CDCl_3_): *δ* 25.1, 28.2, 37.1, 46.5, 49.4, 52.5, 143.4, 151.9.

#### 6.1.5. General synthesis of compounds 13–20

To a suspension of **12** (4.20 mmol) and triethylamine (12.60 mmol) in dry dichloromethane (10 ml), at 0 °C, was added, dropwise, a solution of the properly aryl sulphonyl chloride (5.04 mmol) in dry dichloromethane (2 ml). The reaction mixture was stirred at 0 °C for 2 h and at room temperature for 2 h. The reaction was quenched with distilled water (10 ml) and extracted with dichloromethane (3 × 10 ml). The combined organic layers were washed with distilled water again, dried over anhydrous Na_2_SO_4_ and then the solvent was evaporated off. The crude product was purified by column chromatography on silica gel, using a mixture 95:5 of dichloromethane and methanol as the mobile phase.

##### 6.1.5.1. 1-(phenylsulfonyl)-3-(1*H*-1,2,4-triazol-1-ylmethyl)piperidine (13)

Yellow solid, 38% yield. M.p.: 72–73 °C. ^1^HNMR (CDCl_3_): *δ* 1.23–1.29 (m, 1H, CH); 1.55–1.68 (m, 2H, CH_2_); 1.74–1.82 (m, 1H, CH); 2.28–2.38 (m, 1H, CH); 2.58 (q, *J* = 7.2, 11.7 Hz, 1H, CH); 2.86–2.95 (m, 1H, CH); 3.10 (dd, *J* = 3.6, 11.4 Hz, 2H, CH_2_); 4.06 (dd, *J* = 6.0, 13.8 Hz, 1H, CH), 4.23 (dd, *J* = 7.5, 13.8 Hz, 1H, CH); 7.50–7.60 (m, 3H, CHar); 7.70 (dd, *J* = 1.5, 8.4 Hz, 2H, CHar); 7.95 (s, 1H, CHar); 8.16 (s, 1H, CHar); ^13^C (CDCl_3_): *δ* 22.7, 26.7, 35.3, 46.6, 48.4, 51.2, 127.5, 129.1, 132.8, 136.1, 143.7, 152.8. Anal calcd for C_14_H_18_N_4_O_2_S: C, 54.88; H, 5.92; N, 18.29. Found C, 54.72; H, 5.94; N, 18.19.

##### 6.1.5.2. 1-[(4-methylphenyl)sulphonyl]-3-(1*H*-1,2,4-triazol-1-ylmethyl)piperidine (14)

Yellow oil, 75% yield. ^1^HNMR (CDCl_3_): *δ* 1.18–1.27 (m, 1H, CH); 1.55–1.68 (m, 2H, CH_2_); 1.71–1.83 (m, 1H, CH); 2.26–2.38 (m, 2H, CH_2_); 2.42 (s, 3H, CH_3_); 2.56 (q, *J* = 7.8, 11.7 Hz, 1H, CH); 3.07 (dd, *J* = 3.6, 11.7 Hz, 2H, CH); 4.06 (dd, *J* = 6.3, 13.8 Hz, 1H, CH), 4.23 (dd, *J* = 8.1, 13.8 Hz, 1H, CH); 7.29 (d, *J* = 7.8 Hz, 2H, CHar); 7.58 (d, *J* = 8.1 Hz, 2H, CHar); 7.96 (s, 1H, CHar); 8.18 (s, 1H, CHar). ^13^CNMR (CDCl_3_): *δ* 22.7, 26.7, 29.6, 35.2, 46.7, 48.4, 51.3, 125.8, 127.5, 128.8, 129.7, 129.8, 132.9, 143.7, 151.8. Anal calcd for C_15_H_20_N_4_O_2_S: C, 56.23; H, 6.29; N, 17.49. Found C, 56.37; H, 6.31; N, 17.41

##### 6.1.5.3. 1-[(2,4-dimethoxyphenyl)sulphonyl]-3-(1*H*-1,2,4-triazol-1-ylmethyl)piperidine (15)

Sticky yellow solid, 40% yield. ^1^HNMR (CD_3_OD): *δ* 1.16–1.29 (m, 1H, CH); 1.49–1.59 (m, 1H, CH); 1.63–1.8 (m, 2H, CH_2_); 2.14–2.38 (m, 1H, CH); 2.59 (q, *J* = 8.7, 12.9 Hz, 1H, CH); 2.79–2.87 (m, 1H, CH); 3.65 (dd, *J* = 3.9, 12.3 Hz, 2H, CH_2_); 3.86 (s, 3H, CH_3_); 3.87 (s, 3H, CH_3_); 4.12–4.27 (m, 2H, CH_2_), 6.58 (dd, *J* = 2.1, 8.7 Hz, 1H, CHar); 6.65 (d, *J* = 2.4 Hz, 1H, CHar); 7.67 (d, *J* = 8.7 Hz, 1H, CHar); 8.00 (s, 1H, CHar); 8.45 (s, 1H, CHar). ^13^CNMR (CD_3_OD): *δ* 23.5, 26.7, 36.0, 46.2, 51.2, 54.9, 55.0, 98.8, 104.5, 117.6, 127.6, 132.6, 144.1, 150.9, 158.5, 165.2. Anal calcd for C_16_H_22_N_4_O_4_S: C, 52.44; H, 6.05; N, 17.46. Found C, 52.38; H, 6.03; N, 17.48.

##### 6.1.5.4. 1-[(3-nitrophenyl)sulphonyl]-3-(1*H*-1,2,4-triazol-1-ylmethyl)piperidine (16)

Sticky white solid, 82% yield. ^1^HNMR (CDCl_3_): *δ* 1.22–1.26 (m, 1H, CH); 1.61–1.71 (m, 2H, CH_2_); 1.78–1.82 (m, 1H, CH); 2.33–2.37 (m, 1H, CH); 2.63 (q, *J* = 7.8, 11.7 Hz, 1H, CH); 2.94–2.98 (m, 1H, CH); 3.21–3.28 (m, 2H, CH); 4.07 (ddd, *J* = 6.3, 7.8 Hz, 2H, CH_2_); 7.73 (*t*, *J* = 7.5, 15.9 Hz, 1H, CHar); 7.95 (s, 1H, CHar); 8.04 (d, *J* = 8.4 Hz, 1H, CHar); 8.11 (s, 1H, CHar); 8.43 (d, *J* = 7.2 Hz, 1H, CHar); 8.54 (d, *J* = 4.2 Hz, 1H, CHar). ^13^CNMR (CDCl_3_): *δ* 22.8, 26.6, 35.3, 46.6, 48.5, 51.1, 122.4, 127.3, 130.6, 132.9, 138.8, 143.7, 148.4, 152.4. Anal calcd for C_14_H_17_N_5_O_4_S: C, 47.85; H, 4.88; N, 19.93. Found C, 47.88; H, 4.87; N, 19.94.

##### 6.1.5.5. 1-[(4-methyl-3-nitrophenyl)sulphonyl]-3-(1*H*-1,2,4-triazol-1-ylmethyl)piperidine (17)

Yellow solid, 56% yield. M.p.: 105–106 °C. ^1^HNMR (CDCl_3_): *δ* 1.19–1.28 (m, 1H, CH); 1.59–1.69 (m, 2H, CH_2_); 1.76–1.80 (m, 1H, CH); 2.31–2.35 (m, 1H, CH); 2.60–2.67 (m, 4H, CH + CH_3_); 2.91–2.95 (m, 1H, CH); 3.19 (dd, *J* = 3.6 Hz, 2H, CH); 4.06 (ddd, *J* = 6.0, 8.1 Hz, 2H, CH_2_); 7.51 (d, *J* = 7.8 Hz, 1H, CHar); 7.80 (dd, *J* = 2.1, 8.4 Hz, 1H, CHar); 7.94 (s, 1H, CHar); 8.10 (s, 1H CHar); 8.27 (d, *J* = 2.1 Hz, 1H, CHar); ^13^CNMR (CDCl_3_): *δ* 20.5, 22.7, 26.4, 35.3, 46.6, 48.5, 51.1, 123.7, 131.1, 133.9, 135.8, 138.3, 143.7, 149.2, 152.3. Anal calcd for C_15_H_19_N_5_O_4_S: C, 49.30; H, 5.24; N, 19.17. Found C, 49.32; H, 5.25; N, 19.15.

##### 6.1.5.6. 3-chloro-4-{[3-(1*H*-1,2,4-triazol-1-ylmethyl)piperidin-1-yl]sulphonyl}benzonitrile (18)

White solid, 45% yield. M.p.: 81–82 °C. ^1^HNMR (CDCl_3_): *δ* 1.23–1.32 (m, 1H, CH); 1.51–1.61 (m, 1H, CH_2_); 1.69–1.81 (m, 2H, CH); 2.17–2.22 (m, 1H, CH); 2.76 (q, *J* = 9.3, 12.9 Hz, 1H, CH); 2.98 (*t*, *J* = 6.6, 15.2 Hz, 1H, CH); 3.52–3.58 (m, 2H, CH); 4.17 (dd, *J* = 2.4, 6.6 Hz, 2H, CH_2_); 7.83 (dd, *J* = 1.5, 8.1 Hz, 1H, CHar); 7.98 (s, 1H, CHar); 8.05 (d, *J* = 1.2 Hz, 1H, CHar); 8.11 (d, *J* = 8.1 Hz, 1H, CHar); 8.44 (s, 1H, CHar); 8.54 (d, *J* = 4.2 Hz, 1H, CHar). ^13^CNMR (CDCl_3_): *δ* 26.8, 30.4, 38.9, 49.7, 51.6, 54.5, 119.3, 120.7, 133.8, 135.7, 136.5, 138.4, 144.3, 147.0, 155.6. Anal calcd for C_15_H_16_ClN_5_O_2_S: C, 49.25; H, 4.41; N, 19.14. Found C, 49.21; H, 4.40; N, 19.17.

##### 6.1.5.7. 4-{[3-(1*H*-1,2,4-triazol-1-ylmethyl)piperidin-1-yl]sulphonyl}benzonitrile (19)

White solid, 63% yield. M.p.: 95–96 °C. ^1^HNMR (CDCl_3_): *δ* 1.19–1.27 (m, 1H, CH); 1.57–1.68 (m, 2H, CH_2_); 1.74–1.84 (m, 1H, CH); 2.28–2.37 (m, 1H, CH); 2.58 (q, *J* = 7.8, 11.7 Hz, 1H, CH); 2.86–2.93 (m, 1H, CH); 3.19 (dd, *J* = 3.6, 12.0 Hz, 2H, CH_2_); 4.19 (ddd, *J* = 6.3, 7.5 Hz, 2H, CH_2_); 7.82 (s, 4H, CHar); 7.94 (s, 1H, CHar); 8.10 (s, 1H, CHar); 8.40 (s, 1H, NH). ^13^CNMR (CDCl_3_): *δ* 22.8, 26.7, 35.3, 46.6, 48.4, 51.1, 116.6, 117.1, 128.0, 132.9, 140.7, 143.7, 152.3. Anal calcd for C_15_H_17_N_5_O_2_S: C, 54.36; H, 5.17; N, 21.13. Found C, 54.39; H, 5.15; N, 21.16.

##### 6.1.5.8. *N*-(4-{[3-(1*H*-1,2,4-triazol-1-ylmethyl)piperidin-1-yl]sulphonyl}phenyl)acetamide (20)

White solid, 48% yield. M.p.: 92–93 °C. ^1^HNMR (CDCl_3_): *δ* 1.19–1.27 (m, 1H, CH); 1.55–1.65 (m, 2H, CH_2_); 1.71–1.76 (m, 1H, CH); 2.19 (s, 3H, CH_3_); 2.26–2.34 (m, 1H, CH); 2.52 (q, *J* = 7.8, 11.7 Hz, 1H, CH); 2.85–2.93 (m, 1H, CH); 3.04 (dd, *J* = 3.3, 11.4 Hz, 2H, CH_2_); 4.04 (ddd, *J* = 5.7, 8.2 Hz, 2H, CH_2_); 7.59 (dd, *J* = 9.3, 22.8 Hz, 4H, CHar); 7.94 (s, 1H, CHar); 8.13 (s, 1H, CHar); 8.40 (s, 1H, NH). ^13^CNMR (CDCl_3_): *δ* 22.6, 24.5, 26.7, 35.2, 46.7, 48.4, 51.2, 119.4, 128.6, 130.3, 142.5, 143.8, 152.1, 169.0. Anal calcd for C_16_H_21_N_5_O_3_S: C, 52.88; H, 5.82; N, 19.27. Found C, 52.92; H, 5.81; N, 19.31.

#### 6.1.6. Selected compounds chemical stability

A 0.01 M hydrochloric acid buffer (pH 2), as a non-enzymatic simulated gastric fluid (SGF), a 0.01 M phosphate buffer (pH 7.4) as non-enzymatic simulated intestinal fluid (SIF), and a 0.1 mM sodium hydroxide solution (pH 10) were used in this study. The reactions were initiated by adding 0.1 ml of compound stock solution (1 mg/mL in DMSO) to 1.9 ml aqueous buffer solution, in screw-capped vials at 37 ± 0.5 °C. Samples (5 µL) were withdrawn at appropriate intervals, i.e. after 5 min, 1 h, 2 h, 3 h, 4 h, 6 h, 8 h, and then after 24 h incubation time and analysed by HPLC.

### 6.2. Biological studies

#### 6.2.1. iNOS assay procedure

Recombinant human iNOS was purchased from Enzo Life Sciences, Inc. (New York, USA). The enzyme was diluted in HEPES buffer (pH =7.4) to obtain iNOS 2.5 μg/mL stock solution. To measure iNOS activity, 10 μL of iNOS stock solution were added to 80 μL of 2-[4–(2-hydroxyethyl)piperazin-1-yl]ethanesulfonic acid (HEPES) buffer pH =7.4, containing 0.1 mM CaCl_2_, 1 mM D,L-dithiothreitol (DTT), 0.5 mg/mL BSA, 10 μM flavin mononucleotide (FMN), 10 μM flavin adenine dinucleotide (FAD), 30 μM tetrahydrobiopterin (BH4), 10 μg/mL calmodulin (CaM), 10 μM L-Arg. Then, 10 μL of a 10 μM solution of 1400 W as the reference compound or of the test compound were added to the enzyme assay solution, followed by pre-incubation of 15 min at 37 °C. Each reaction was initiated by the addition of 10 μL of nicotinamide adenine dinucleotide phosphate (NADPH) 7.5 mM, carried out at 37 °C for 20 min, and stopped by adding 500 µL of ice-cold CH_3_CN. The mixture was brought to dryness under vacuum and eventually stored at −20 °C, before the fluorescence derivatization. The o-phthalaldehyde-N-acetylcysteine (OPA/NAC) reagent for fluorescence derivatization was prepared with the molar ratio of 1:3, reacting 5 ml of methanolic OPA solution and 20 ml of 0.2 M borate buffer containing 0.1 g of NAC for 90 min to final pH 9.3 ± 0.05. The OPA/NAC solution was stored at 4 °C and saved for no longer than seven days. 600 μL of HPLC grade water were added to the residue of the enzymatic assay and centrifuged at 1,610 x g for 20 min. The fluorescence reaction is realised stirring 190 µL of supernatant and 60 µL of OPA/NAC solution for 5 min.

#### 6.2.2. Aromatase assay

The aromatase inhibitory activity of the novel compounds was determined using a commercial fluorimetric kit (Aromatase CYP19A Inhibitor Screening kit, BioVision, Milpitas, CA, USA). The assay utilises a fluorogenic aromatase substrate, that is converted into a highly fluorescent metabolite detected in the visible range (Ex/Em = 488/527 nm), ensuring a high signal-to-background ratio with little interference by autofluorescence. The inhibition assay was performed as reported in previous works^10^^,^^12^.

#### 6.2.3. Cell Cultures

Human breast cancer MCF-7 (HTB-22™) cells were purchased from ATCC® and maintained in Dulbecco’s Modified Eagle Medium (DMEM) high glucose supplemented with 10% of foetal bovine serum (FBS) and 1% of penicillin/streptomycin (all from Gibco-Thermofisher Scientific, MD, USA) at 37 °C and 5% CO_2_.

#### 6.2.4. Cell metabolic activity (MTT assay)

Cell metabolic activity of MCF-7 human breast cancer cells was assessed by MTT (3–(4,5-dimethylthiazol-2-yl)-2,5-diphenyltetrazolium bromide) test (Sigma Aldrich, Milan, Italy). Cells were seeded (0.1 × 10^4^/well) in a 96-well tissue culture-treated plate (Falcon^®^, Corning Incorporated, NY, USA) and let them adhere for 24 h. Next, the cell monolayer was incubated in the presence of loading concentrations of compounds **2**, **5** and **6** (0–400 µM) diluted in DMSO (final concentration 0.2%) for 24, 48 and 72 h. After the exposure time, cells were incubated with 100 μL/well of MTT (5 mg/mL) 1:10 with fresh growth medium (final concentration 0.5 mg/mL) for 4 h at 37 °C and 5% CO_2_. Finally, the MTT solution was removed and replaced with 100 µL/well of DMSO. Cells were incubated for an additional 20 min at 37 °C and 5% CO_2_ and afterward gently swirled for 10 min at room temperature. The optical density was measured at 540 nm by means of a spectrophotometer (Multiskan GO, Thermo Scientific, Monza, Italy). Results were expressed as the percentage of cells in the presence of vehicle (DMSO) set as 100% and each experiment was performed in triplicate (*n* = 3). Concentration-response curves and IC_50_ were fitted and calculated with the GraphPad Prism 5.0 (GraphPad Software, San Diego, CA, USA)^27^.

#### 6.2.5. Cytotoxicity test (lactate dehydrogenase release)

After 24 h, cell supernatants were collected from the MTT plate, centrifuged at 450 × *g* for 4 min and stored on ice. To quantify the cytotoxicity of loading concentrations of compounds **2**, **5** and **6** (0–400 µM), the CytoTox 96^®^ Non-Radioactive Cytotoxicity Assay (Promega Corporation, WI, USA) was performed. The CytoTox 96^®^ Assay quantitatively measures lactate dehydrogenase (LDH), a stable cytosolic enzyme released upon cell lysis. The absorbance signal resulting from the conversion of a tetrazolium salt (iodonitrotetrazolium violet; INT) into a red formazan product was measured at 490 and 690 nm (background) with a spectrophotometer (Multiskan GO, Thermo Scientific, Monza, Italy). The results were normalised on MTT absorbances and expressed as fold increases on the LDH released by cultures in the presence of vehicle (DMSO) set as 1.

#### 6.2.6. Statistics

Statistics were performed using the one-way analysis of variance (ANOVA) followed by Tukey’s multiple comparison test by means of the Prism 5.0 software (GraphPad, San Diego, CA, USA). Results are the mean values ± standard deviations. Values of *p* ≤ 0.05 were considered statistically significant.

### 6.3. Microbiology

#### 6.3.1. Yeast strains and culture conditions

The antifungal activity of the most representative compounds was evaluated versus one ATCC 90028 strain and 15 clinical isolates belonging to the most clinically relevant *Candida* spp. (*C. albicans*, *C. tropicalis*, and *C. glabrata*). All strains were stored at −80 °C in Sabouraud Broth (Oxoid LTD, Basingstoke, Hampshire, England) with 20% of glycerine until their use. The strains were grown on Sabouraud CAF agar (Thermo Fisher Scientific Waltham, MA, USA), and incubated for 24–48 h at 37 °C. The study did not require ethical approval, because all the isolates were previously obtained as part of routine diagnostic microbiology^28^^,^^29^.

#### 6.3.2. Antifungal activity

The *in vitro* antifungal activity was determined by broth microdilution method with RPMI 1640 (Sigma-Aldrich. ST Louis, MO, USA) as recommended by EUCAST, and the E. Def 7.3, method document (http://www.eucast.org). Ninety-six-well polystyrene microtitre plates, containing serial dilutions of the compounds, were inoculated with each strain to yield the appropriate density (0.5–2.5 × 10^5^ CFU/mL) in a 200 µL final volume. Each plate included the following controls: (i) inoculum suspension (growth control), (ii) drug-free medium, (iii) medium with drug, and (iv) distilled water (used for the inoculum preparation). Fluconazole was used as a reference drug according to the EUCAST guidelines. The plates were incubated for 24 h at 37 °C, and then read with a plate reader at 530 nm. The MIC for all isolates was defined as the lowest concentration giving inhibition of growth of ≥50% of that of the drug-free control. Three independent experiments were performed in triplicate.

### 6.4. Molecular modelling studies

#### 6.4.1. Preparation of protein structures

The crystal structure of hCYP19A1 in complex with 4-androstene-3–17-dione (pdb: 3s79; 2.75 Å), human iNOS in complex with ethylisothiourea (pdb: 4nos; 2.25 Å), *C. albicans* CYP51 in complex with itraconazole (pdb: 5v5z; 2.90 Å), and *C. albicans* carbonic anhydrase CaNce103p without a co-crystallized ligand (pdb: 6gwu; 2.20 Å) were obtained from the RCSB Protein Data Bank. Subsequently, the structures were prepared using the protein preparation tool of Schrödinger (v2021-1, Schrödinger, Inc., New York, USA). All water and buffer molecules were omitted. Subunit A was retained and all other subunits, if present, were omitted. In the case of 6gwu, a water molecule was added to the zinc ion at the position of the sulphur atom in the co-crystallized buffer beta-mercaptoethanol. Subsequently, hydrogen atoms were added and the system was minimised using the OPLS4 forcefield.

#### 6.4.2. Docking studies

The two stereoisomers of compound **2** were prepared using the LigPrep tool of Schrödinger and minimised with the OPLS4 forcefield. Subsequently, both stereoisomers were docked into the active sites of hCYP19A1, iNOS, CaCYP51 and CaNce103p using the Glide tool of Schrödinger with the SP settings. The three highest scoring poses were obtained for each ligand and the poses were subsequently minimised using the Prime tool and MM-GBSA forcefield. To this end, the ligand and all residues within 4 Å, except the zinc ion, zinc binding residues and zinc-bound water, were unrestrained.

#### 6.4.3. Molecular dynamics simulations

The ligand-enzyme complexes obtained with the docking procedure were subjected to a 50 ns MD simulation using Desmond. The complex was first placed in an orthorombic box (at least 10 Å between complex and boundary) and then filled with Tip5P water molecules and 0.15 M NaCl. The amount of Na or Cl atoms was adjusted to create a neutral system. Afterwards, all heavy atoms were restrained and the system was minimised for 100 ps using the OPLS4 forcefield. Finally, the system was simulated for 50 ns under isothermic (Nose–Hoover chain, 1 ps relaxation time) and isobaric (Martyna–Tobial–Klein, 2 ps relaxation time, isotropic coupling) conditions without restraints. Snapshots were saved every 100 ps.

## Supplementary Material

Supplemental Material
