# Supplementary material for: New azolyl-derivatives as multitargeting agents against breast cancer and fungal infections: synthesis, biological evaluation and docking study
Source: J Enzyme Inhib Med Chem. 2021 Jul 21;36(1):1632–45. doi: 10.1080/14756366.2021.1954918 (PMC8300937; doi:10.1080/14756366.2021.1954918)
Supplement: Supplemental Material [file IENZ_A_1954918_SM8737.pdf]

Supplementary Materials

New azolyl-derivatives as multitargeting agents against breast cancer and fungal infections: synthesis, biological evaluation and docking study

Cristina Maccallini[1], Marialucia Gallorini[1], Francesca Sisto[2], Atilla Akdemir[3], Alessandra Ammazzalorso[1], Barbara De Filippis[1], Marialuigia Fantacuzzi[1], Letizia Giampietro[1], Simone Carradori[1,3,]*, Amelia Cataldi[1], Rosa Amoroso[1]

[1]*Department of Pharmacy, University "G. d'Annunzio" of Chieti -Pescara, Via dei Vestini 31, 66100 Chieti, Italy*

[2]*Dipartimento di Scienze Biomediche, Chirurgiche ed Odontoiatriche, University of Milan, 20122 Milan, Italy*

[3]*Bezmialem Vakif University, Computer-aided drug discovery laboratory, Department of Pharmacology, Faculty of Pharmacy, 34093 Istanbul, Turkey*

***Corresponding Author:** Simone Carradori; simone.carradori@unich.it

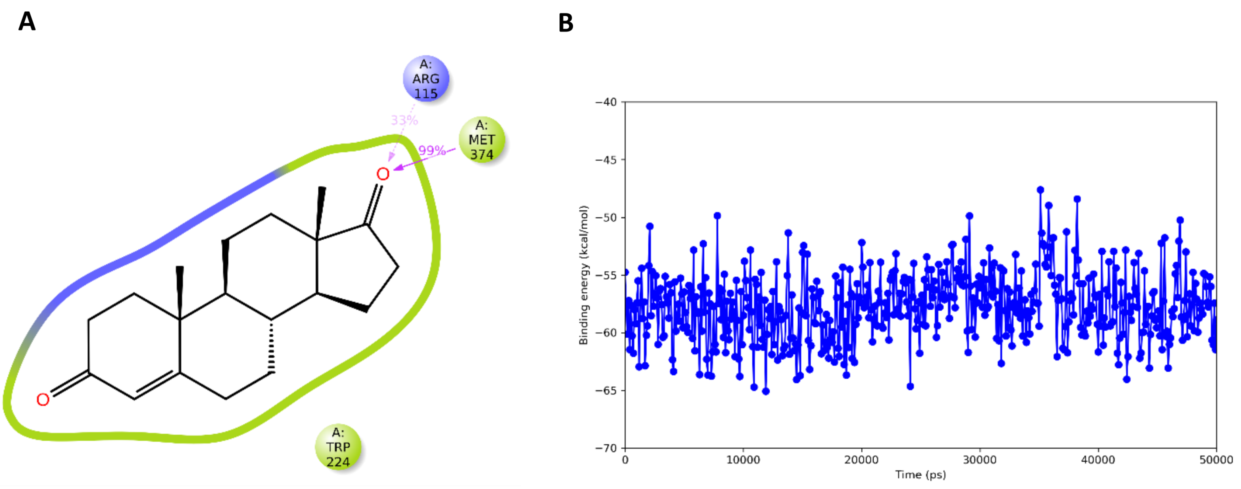

Figure S1. The binding interactions (**panel A**) and binding energy (**panel B**) of compound **4-androstene-3-17-dione** with the hCYP19A1 active site during the 50 ns MD simulation. Hydrophobic amino acids are indicated in green and cationic amino acids are indicated in purple.

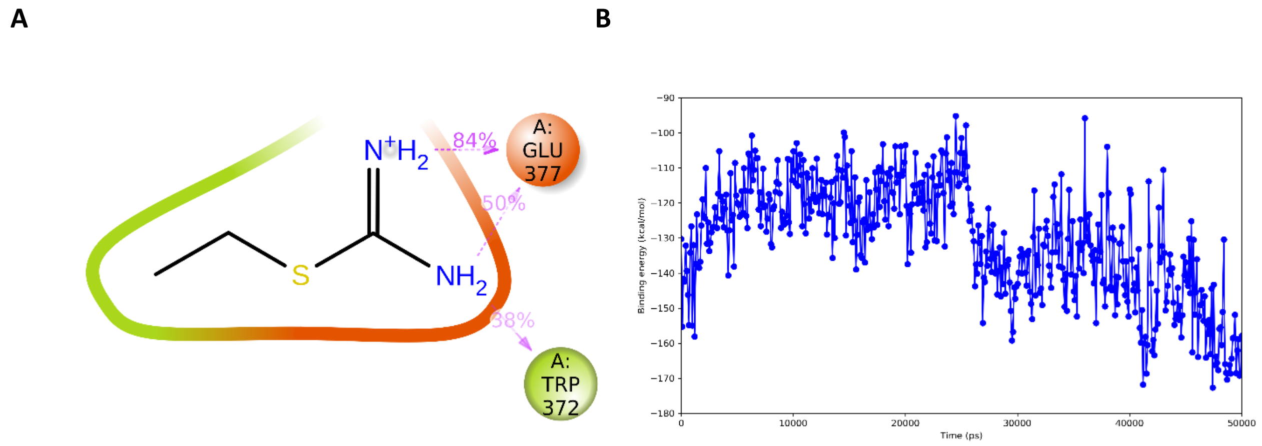

Figure S2. The binding interactions (**panel A**) and binding energy (**panel B**) of compound **ethylisothiourea** with the iNOS active site during the 50 ns MD simulation. Hydrophobic amino acids are indicated in green and anionic amino acids are indicated in red.

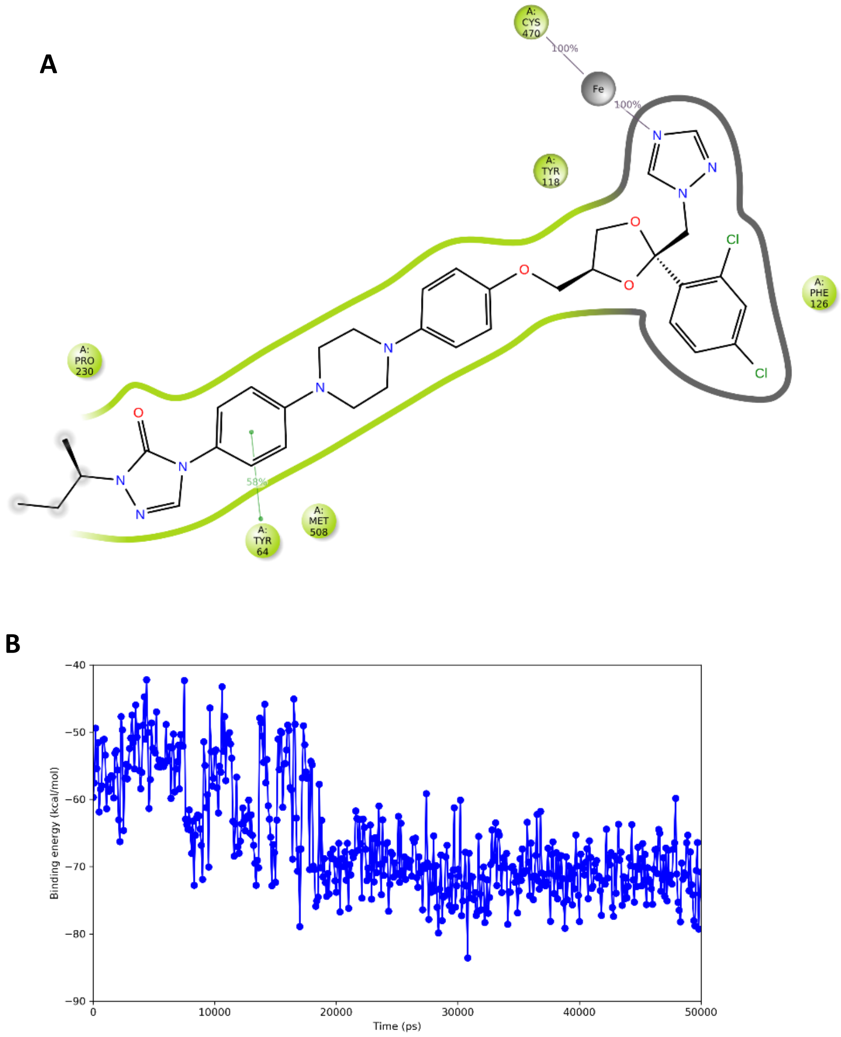

Figure S3. The binding interactions (**panel A**) and binding energy (**panel B**) of compound **itraconazole** with the CaCYP51 active site during the 50 ns MD simulation. Hydrophobic amino acids are indicated in green.

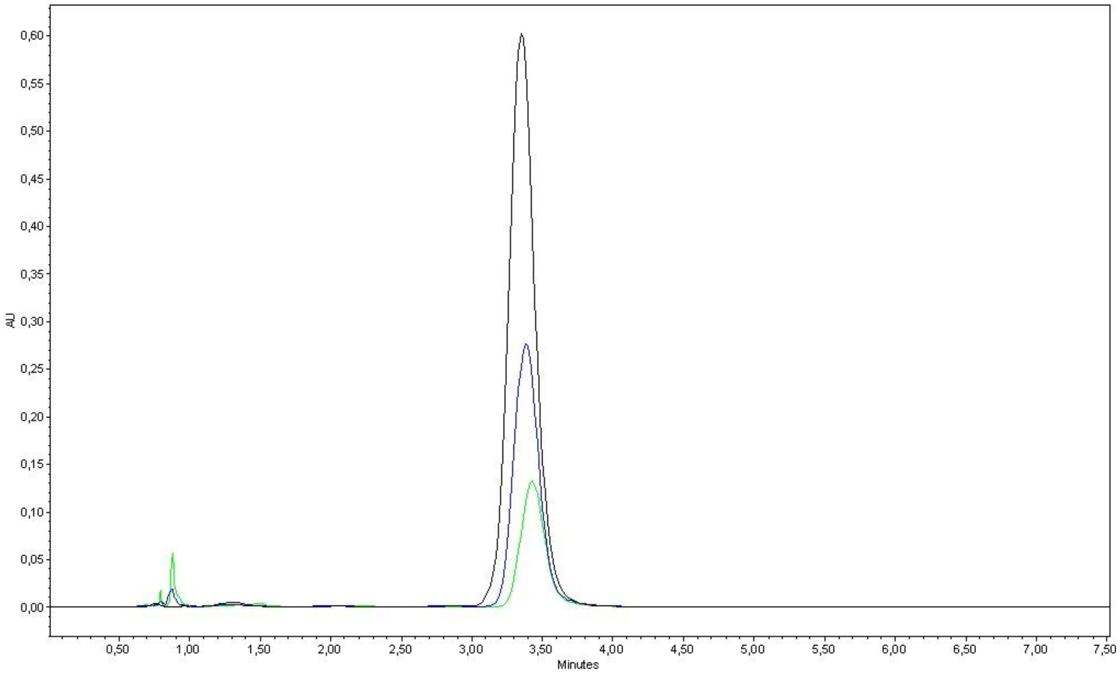

Figure S4. Compound 5 chemical stability in NaOH (pH=9): superimposition of selected chromatograms recorded after 5’(black), 1 h (blue) and 2 h (green) incubation time at 37° C.

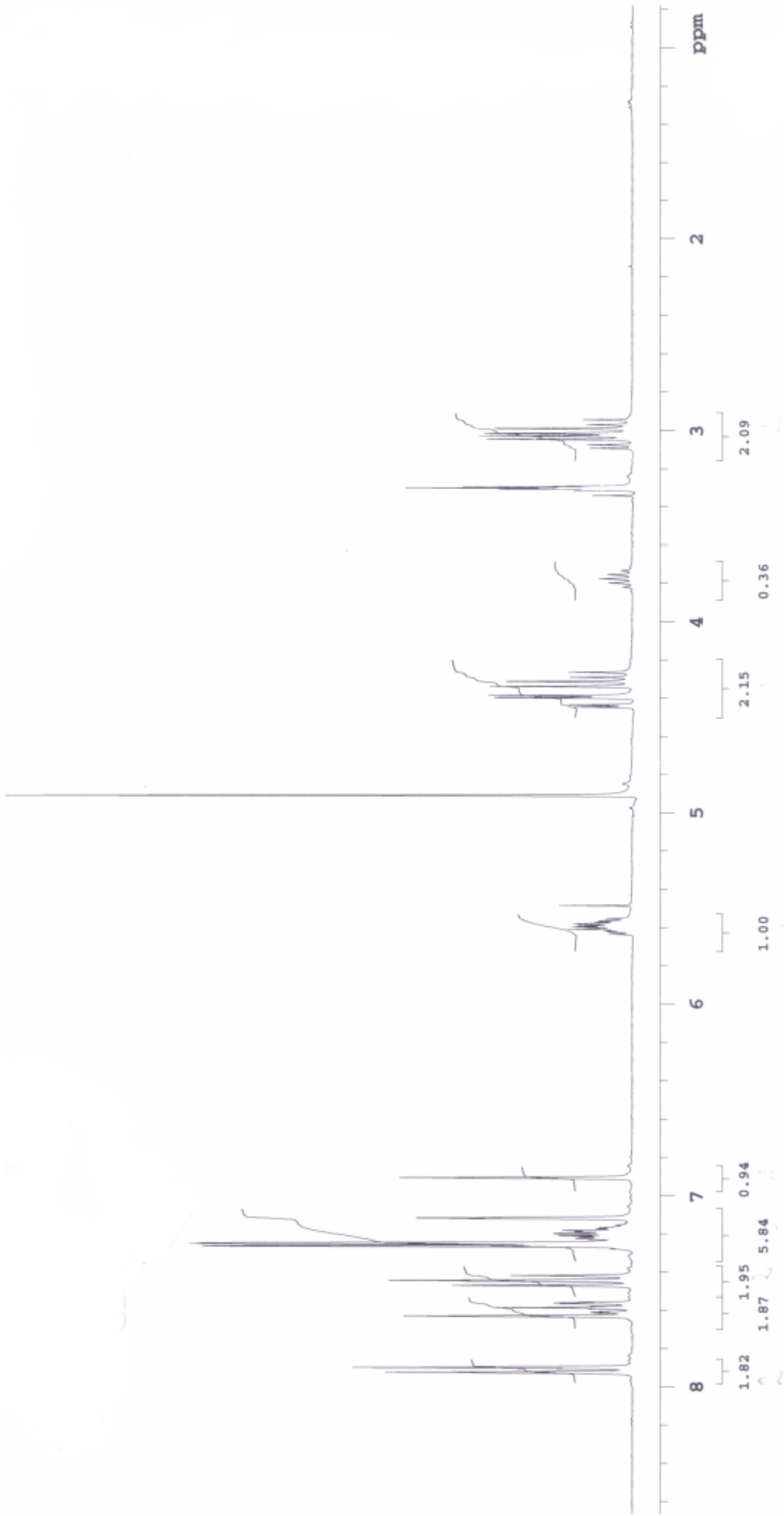

Compound 2

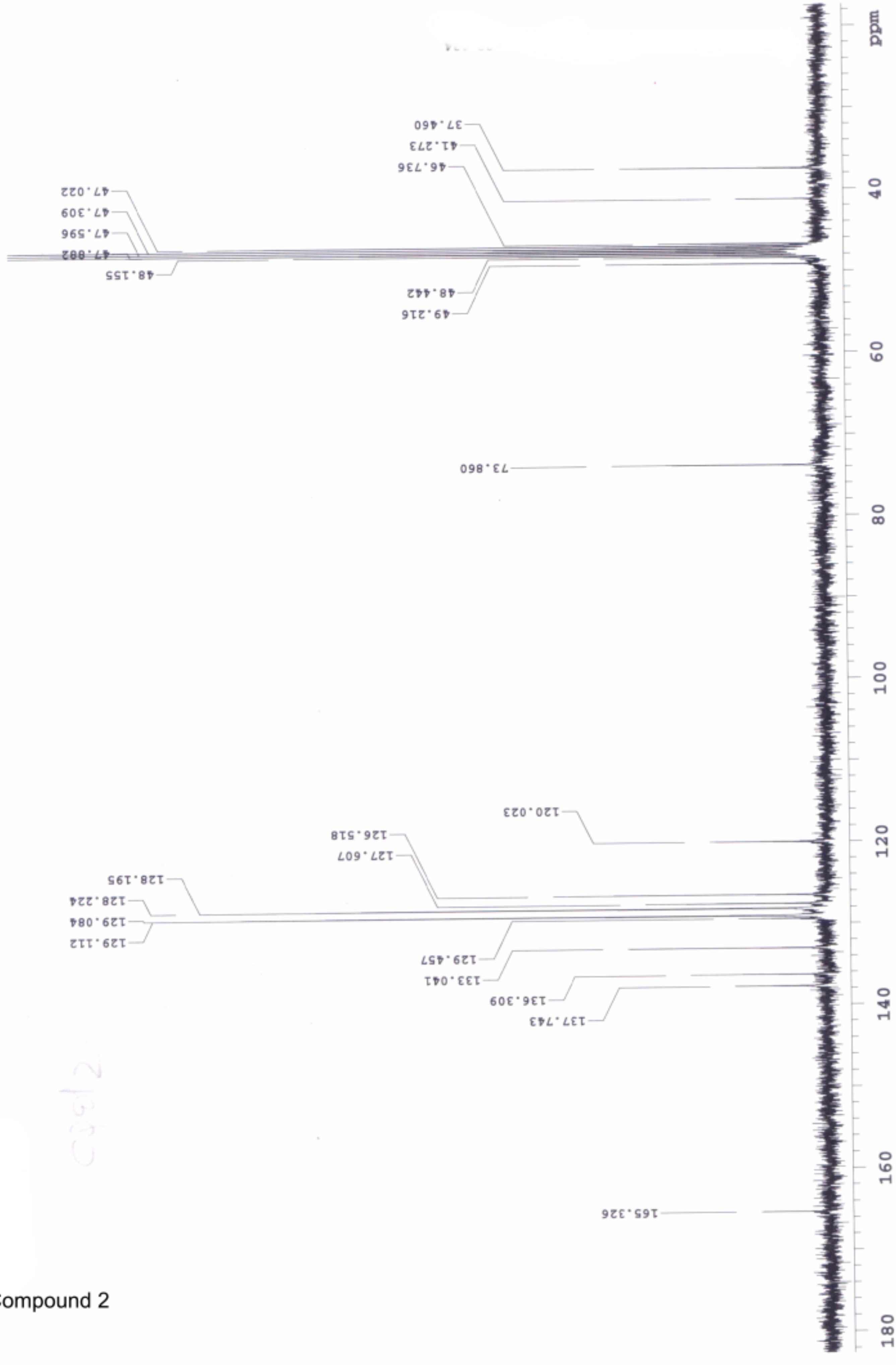

ppm
37.460
41.273
46.736
47.022
47.309
47.596
47.882
48.155
48.442
49.216
73.860
120.023
126.518
127.607
128.195
128.224
129.084
129.112
129.457
133.041
136.309
137.743
165.326
40
60
80
100
120
140
160
180

Compound 2

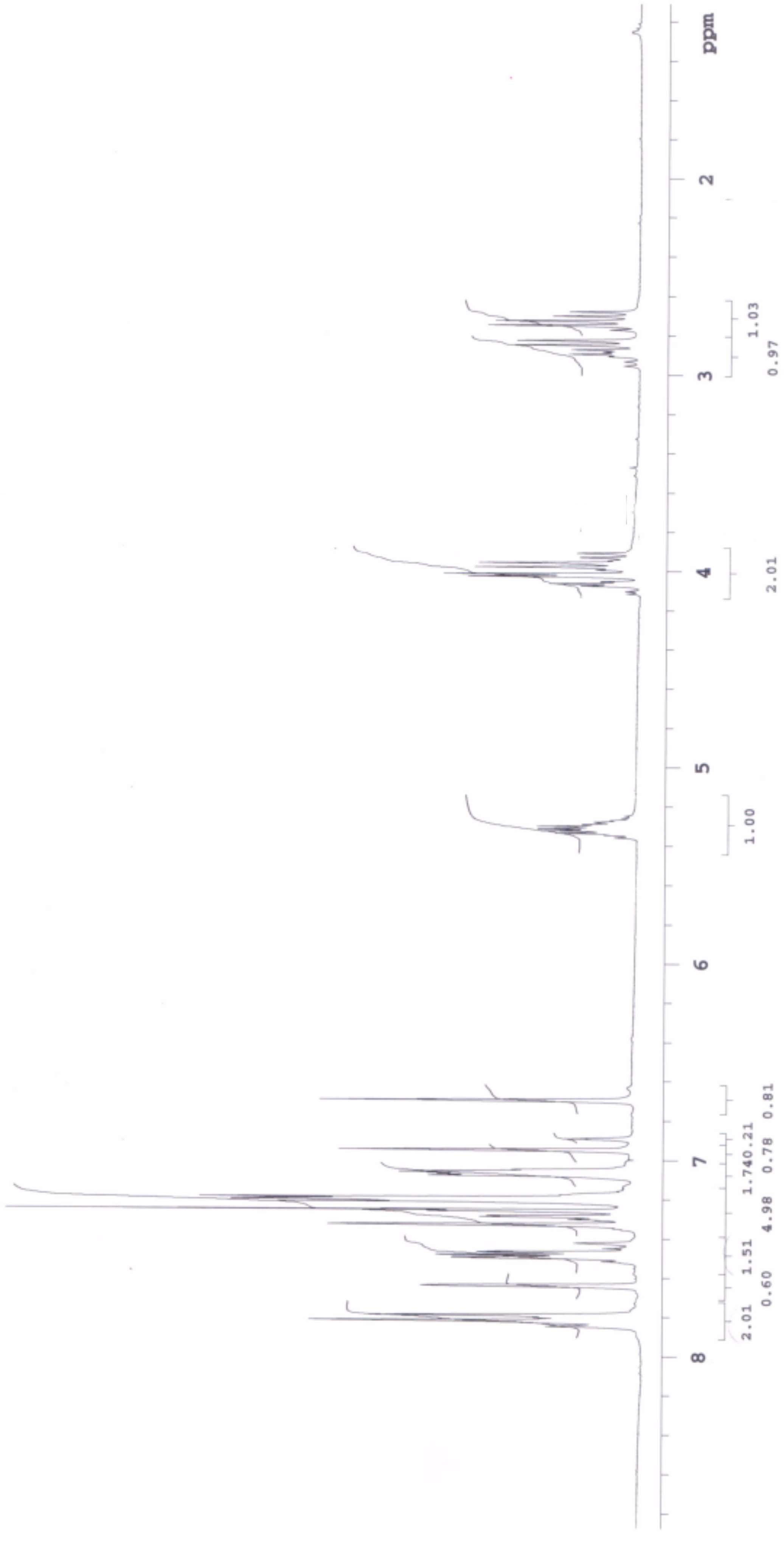

Compound 3

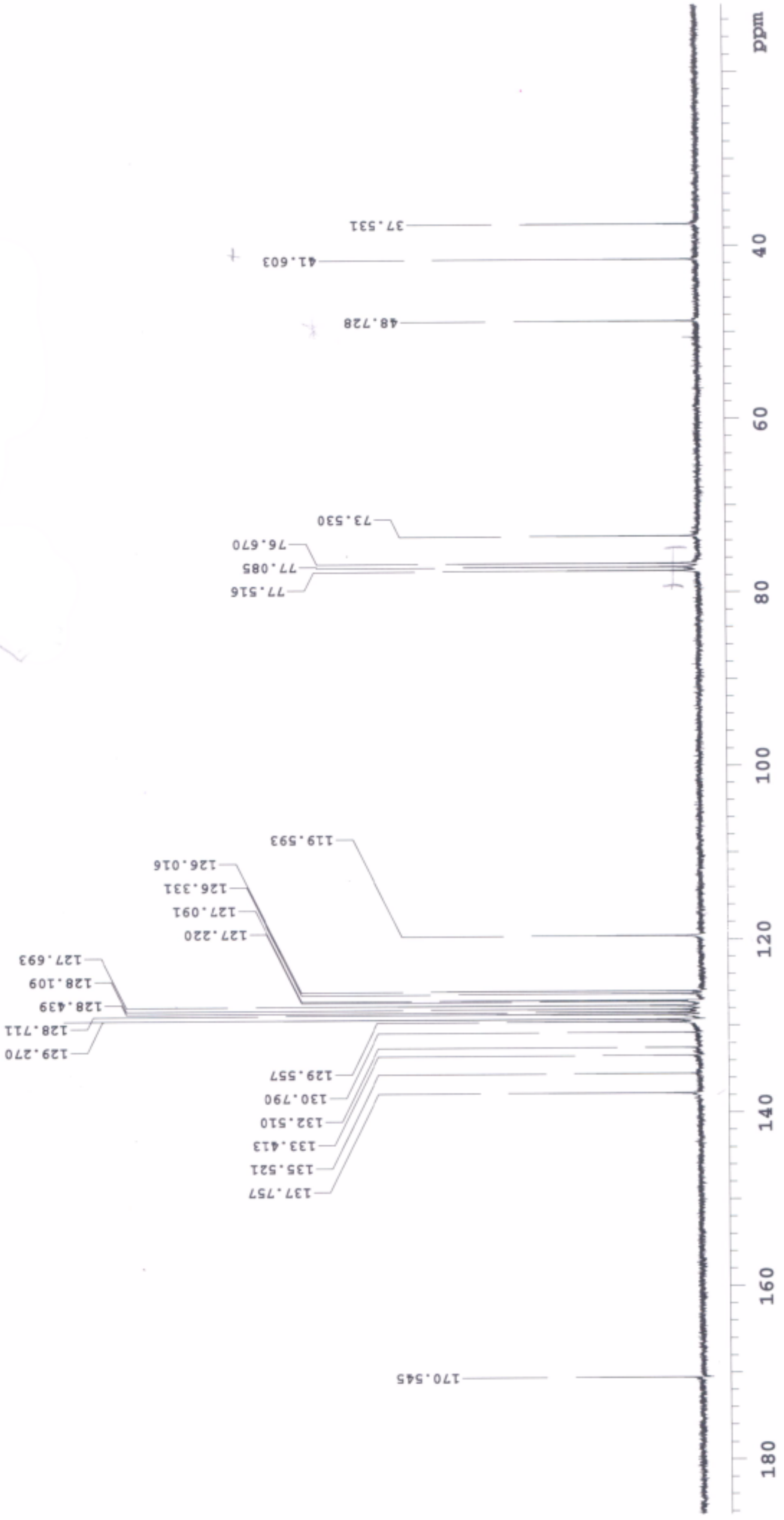
37.531
41.603
48.728
73.530
76.670
77.085
77.516
119.593
126.016
126.331
127.091
127.220
127.693
128.109
128.439
128.711
129.270
129.557
130.790
132.510
133.413
135.521
137.757
170.545
ppm
40
60
80
100
120
140
160
180

Compound 3

Compound 4

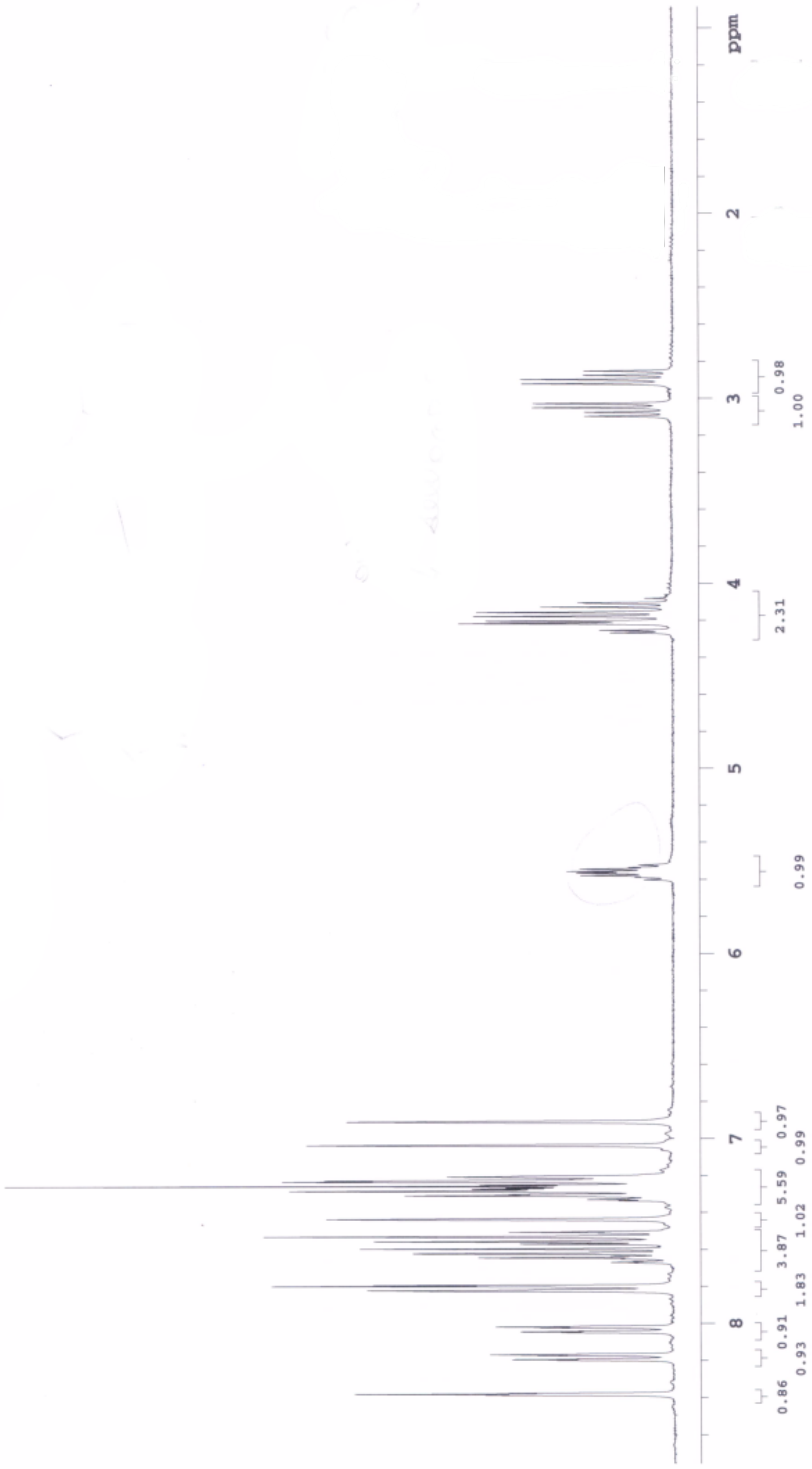

Compound 4

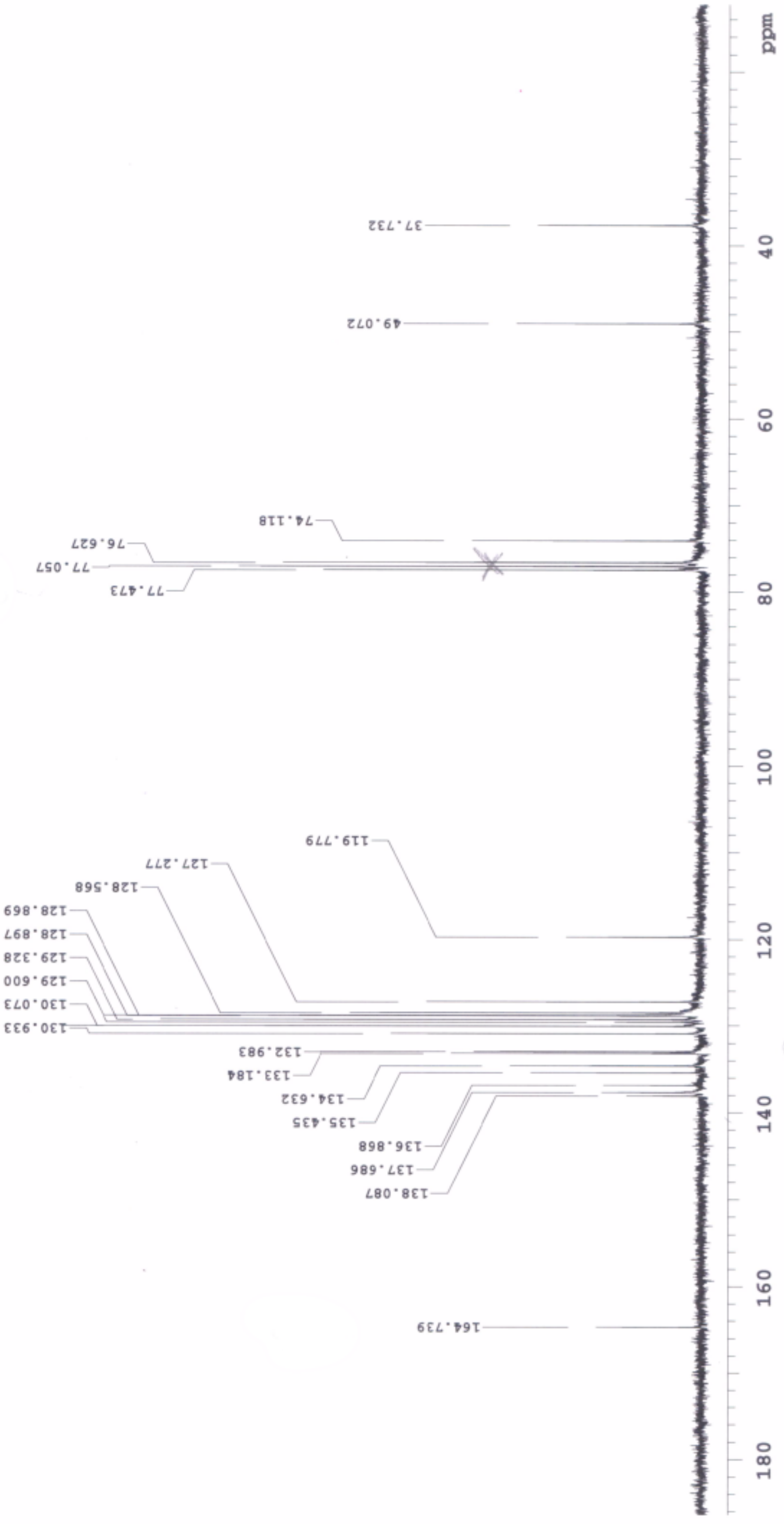
37.732
49.072
74.118
76.627
77.057
77.473
119.779
127.277
128.568
128.869
128.897
129.328
129.600
130.073
130.933
132.983
133.184
134.632
135.435
136.868
137.686
138.087
164.739
ppm
40
60
80
100
120
140
160
180

Compound 5

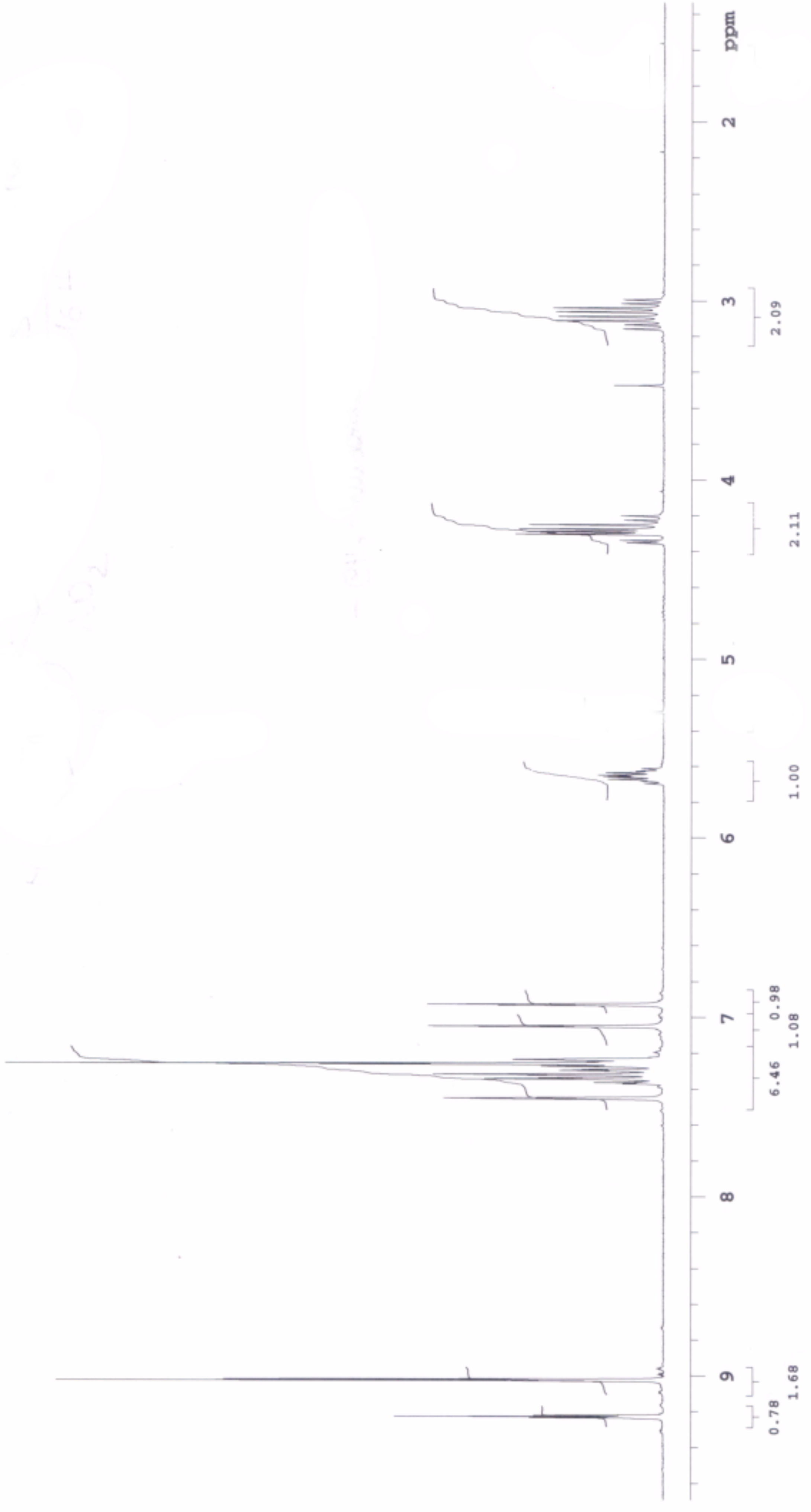

Compound 5

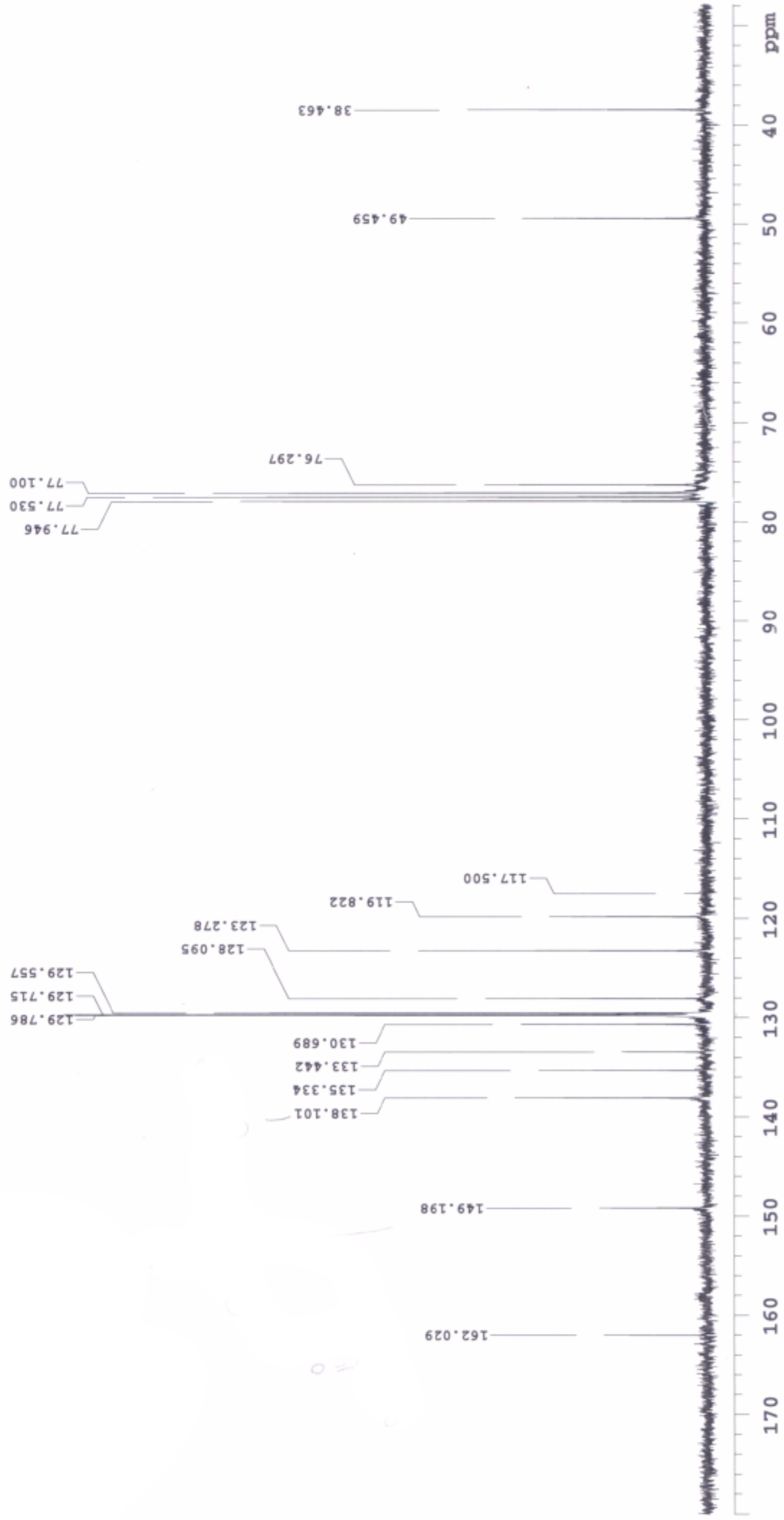
38.463
49.459
76.297
77.100
77.530
77.946
117.500
119.822
123.278
128.095
129.557
129.715
129.786
130.689
133.442
135.334
138.101
149.198
162.029
ppm
40
50
60
70
80
90
100
110
120
130
140
150
160
170

Compound 6

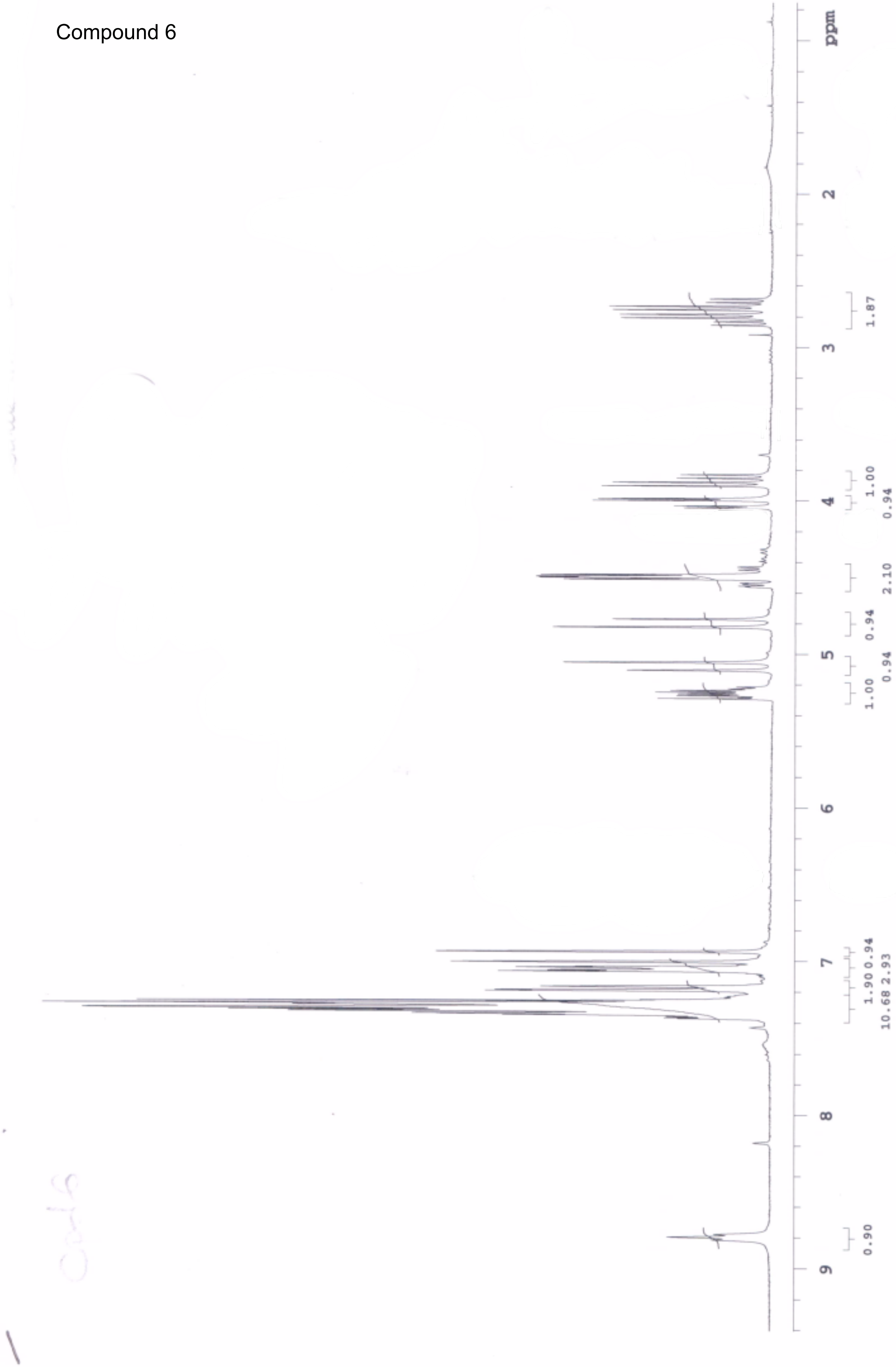

Compound 6

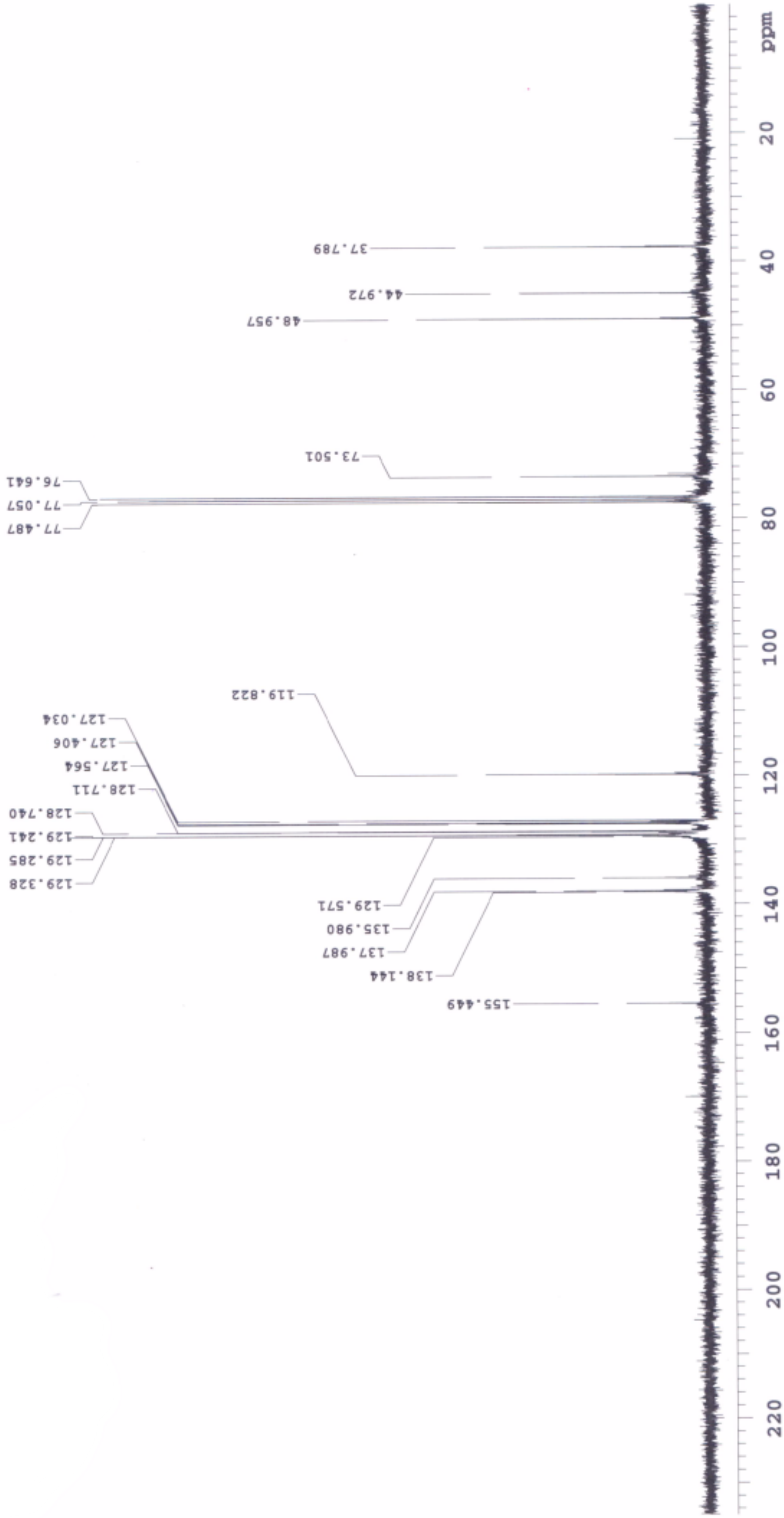

37.789
44.972
48.957
73.501
76.641
77.057
77.487
119.822
127.034
127.406
127.564
128.711
128.740
129.241
129.285
129.328
129.571
135.980
137.987
138.144
155.449
ppm
20
40
60
80
100
120
140
160
180
200
220

Compound 7

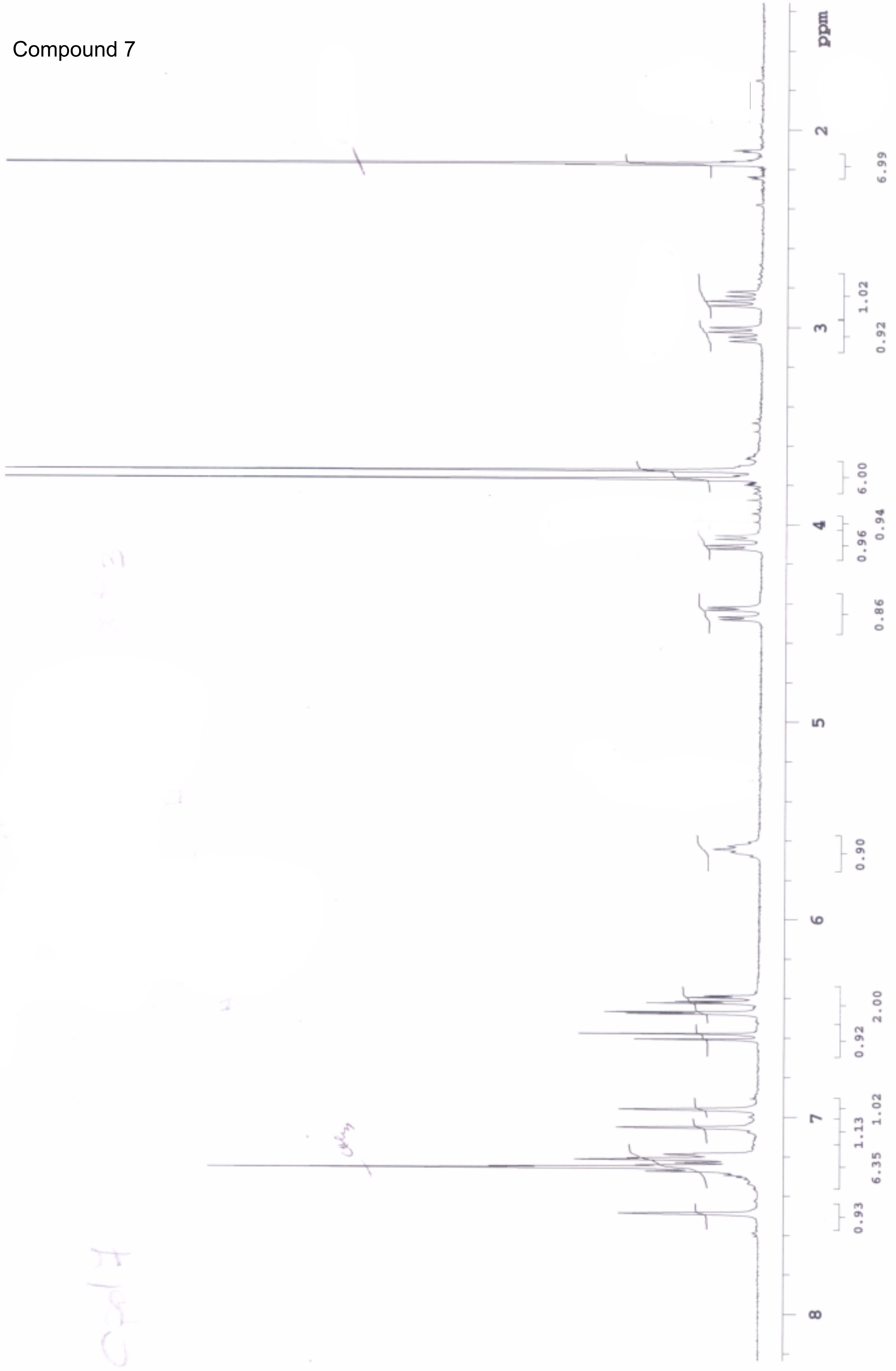
ppm
2
3
4
5
6
7
8
6.99
1.02
0.92
6.00
0.96
0.94
0.86
0.90
2.00
0.92
1.13
1.02
6.35
0.93

Compound 7

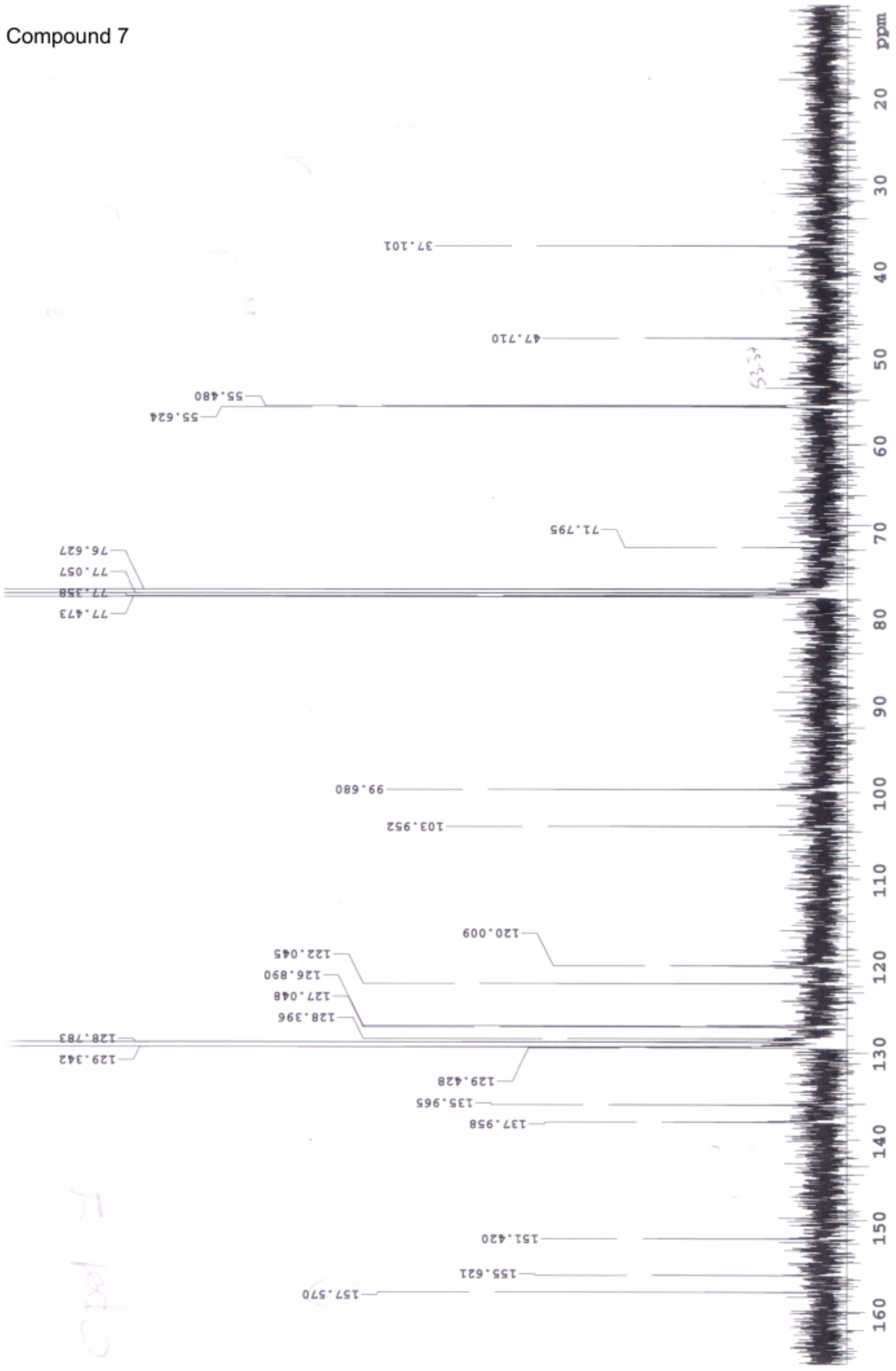
37.101
47.710
55.480
55.624
71.795
76.627
77.057
77.358
77.473
99.680
103.952
120.009
122.045
126.890
127.048
128.396
128.783
129.342
129.428
135.965
137.958
151.420
155.621
157.570
ppm
20
30
40
50
60
70
80
90
100
110
120
130
140
150
160

Compound 8

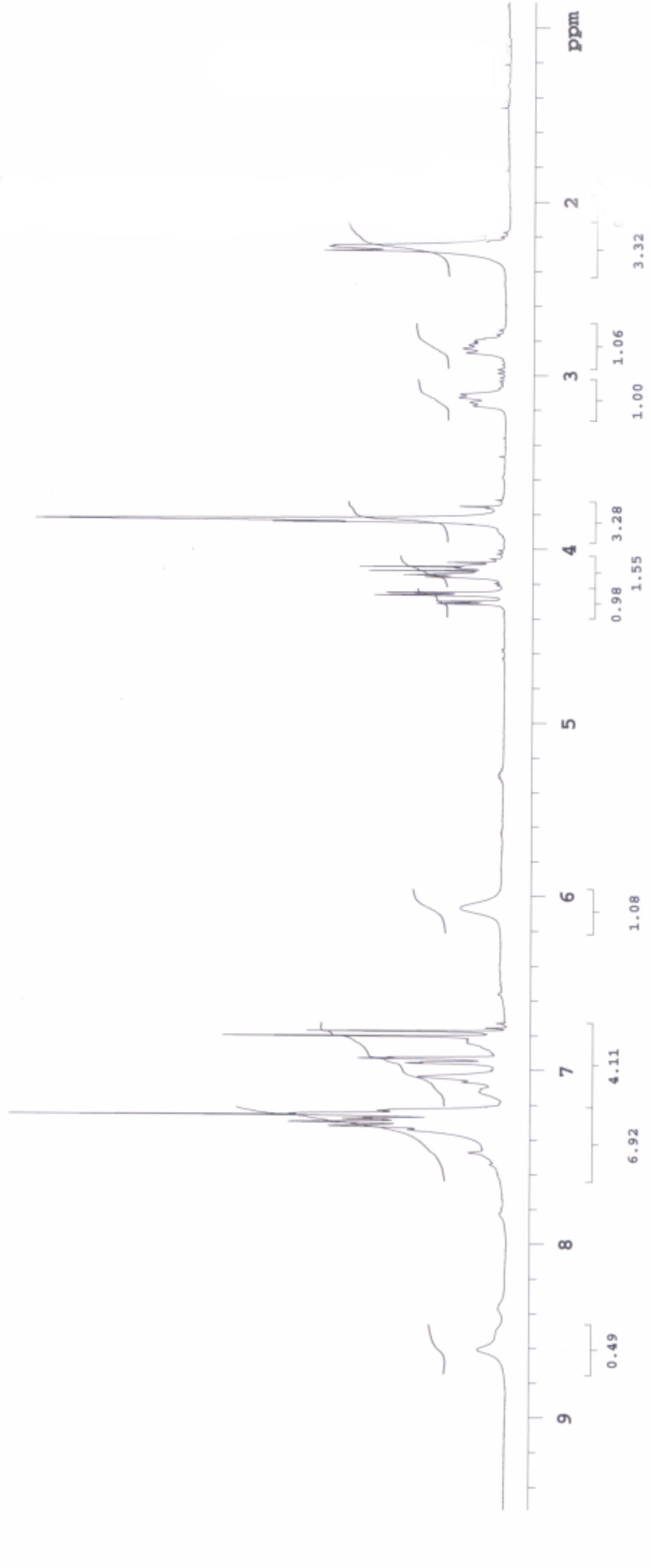

Compound 8

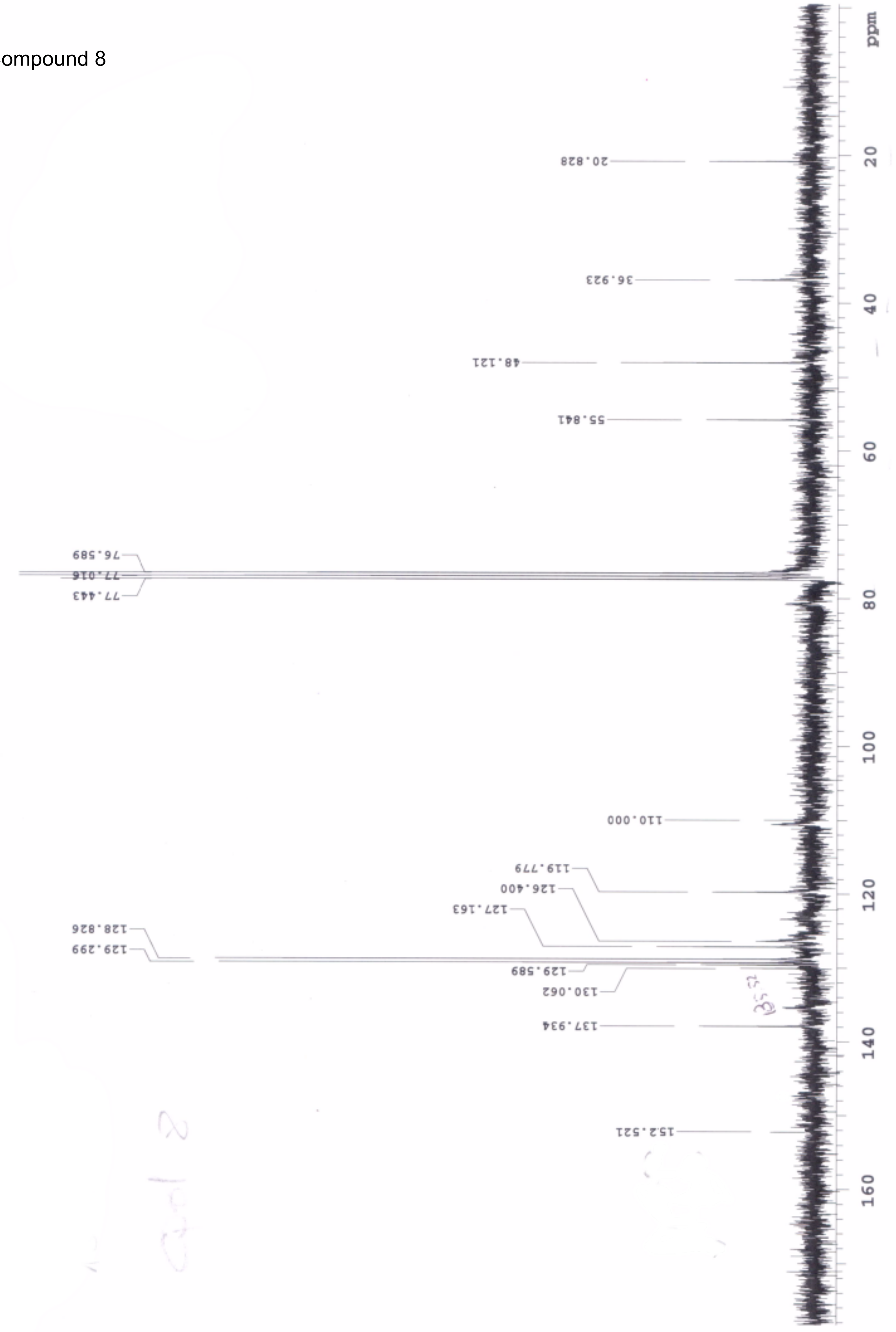

20.828
36.923
48.121
55.841
76.589
77.016
77.443
110.000
119.779
126.400
127.163
128.826
129.299
129.589
130.062
137.934
152.521
ppm
20
40
60
80
100
120
140
160

Compound 13

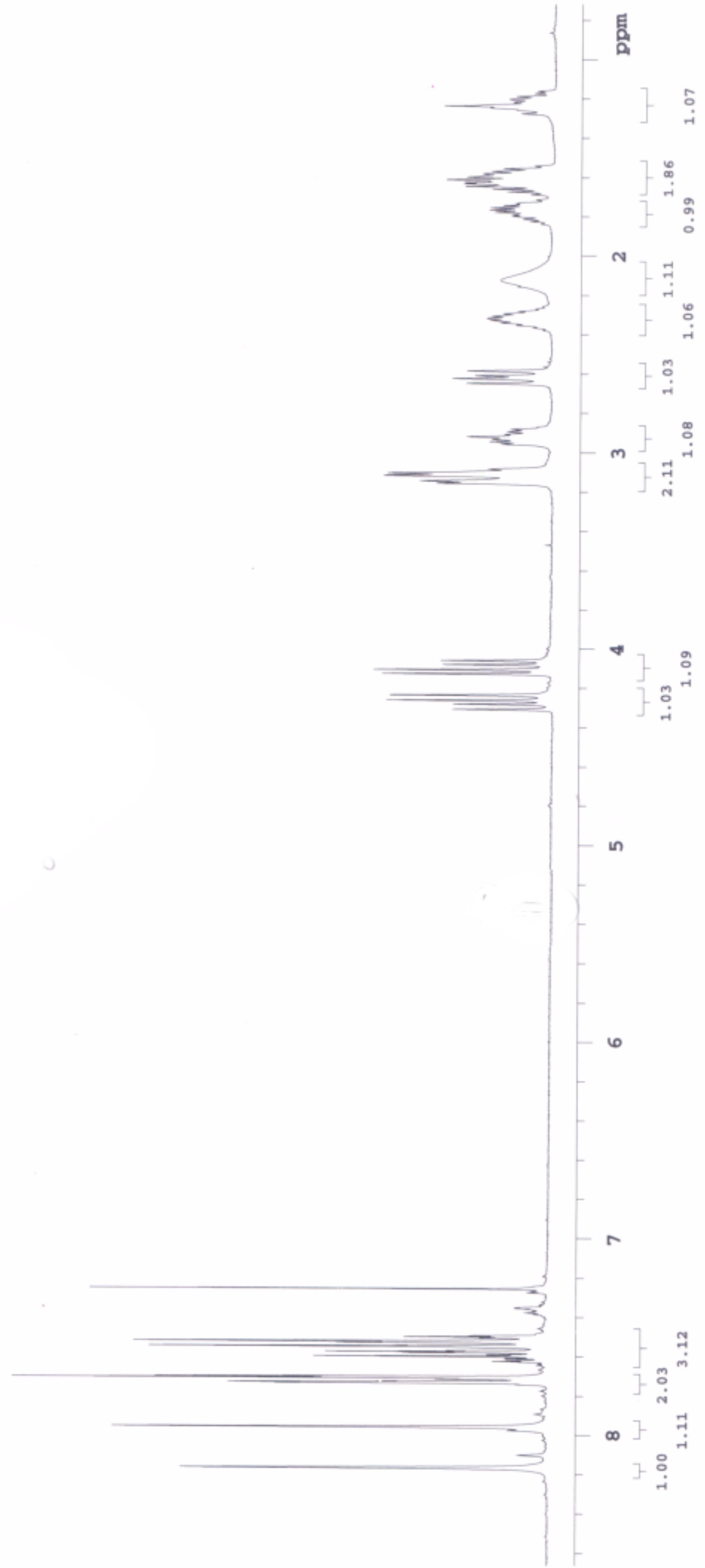

ppm
2
3
4
5
6
7
8
1.07
1.86
0.99
1.11
1.06
1.03
1.08
2.11
1.09
1.03
3.12
2.03
1.11
1.00

Compound 13

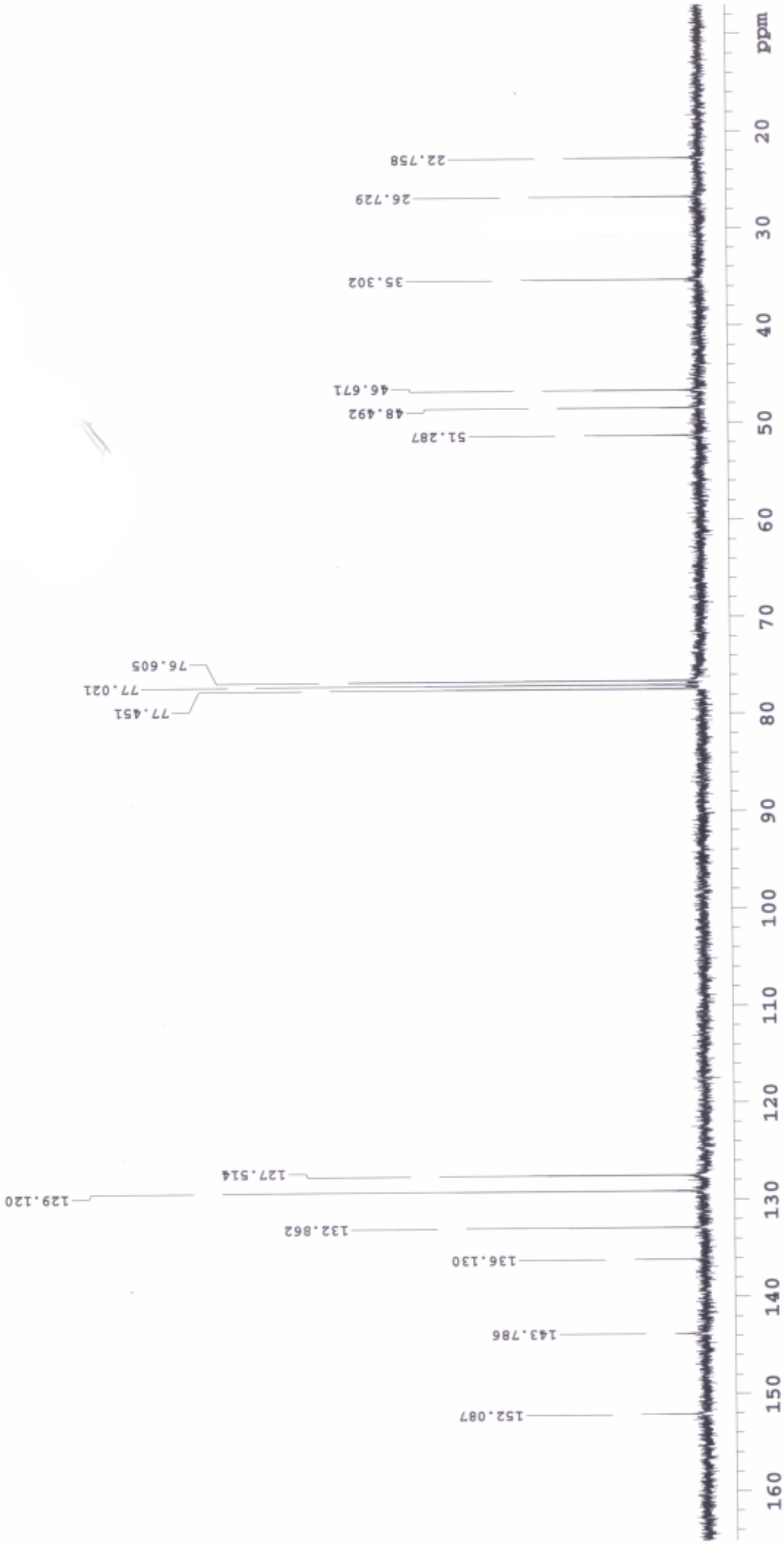
ppm
22.758
26.729
35.302
46.671
48.492
51.287
76.605
77.021
77.451
127.514
129.120
132.862
136.130
143.786
152.087

Compound 14

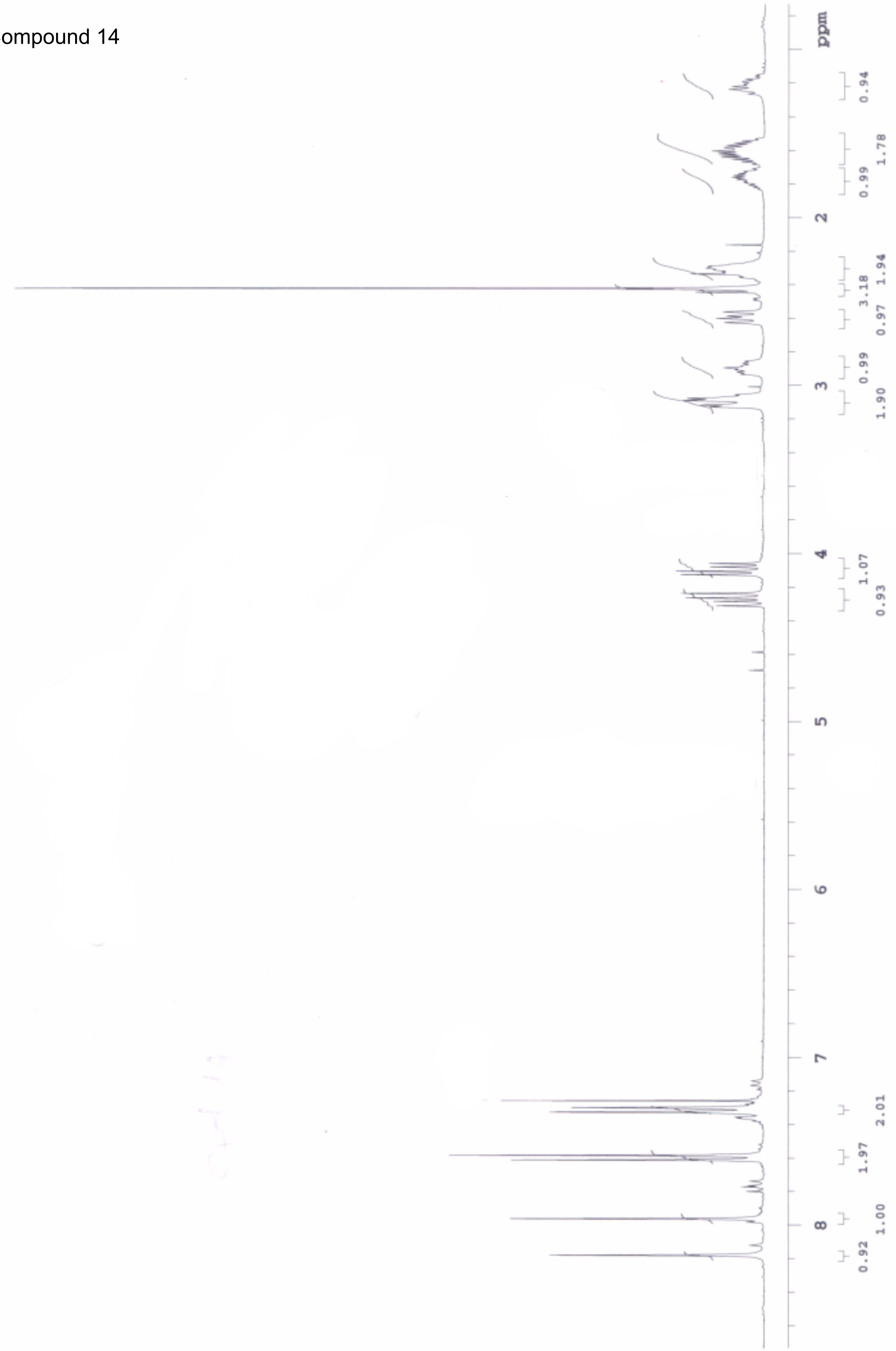
ppm
2
3
4
5
6
7
8
0.94
0.99
1.78
3.18
1.94
0.97
0.99
1.90
1.07
0.93
2.01
1.97
1.00
0.92

Compound 14

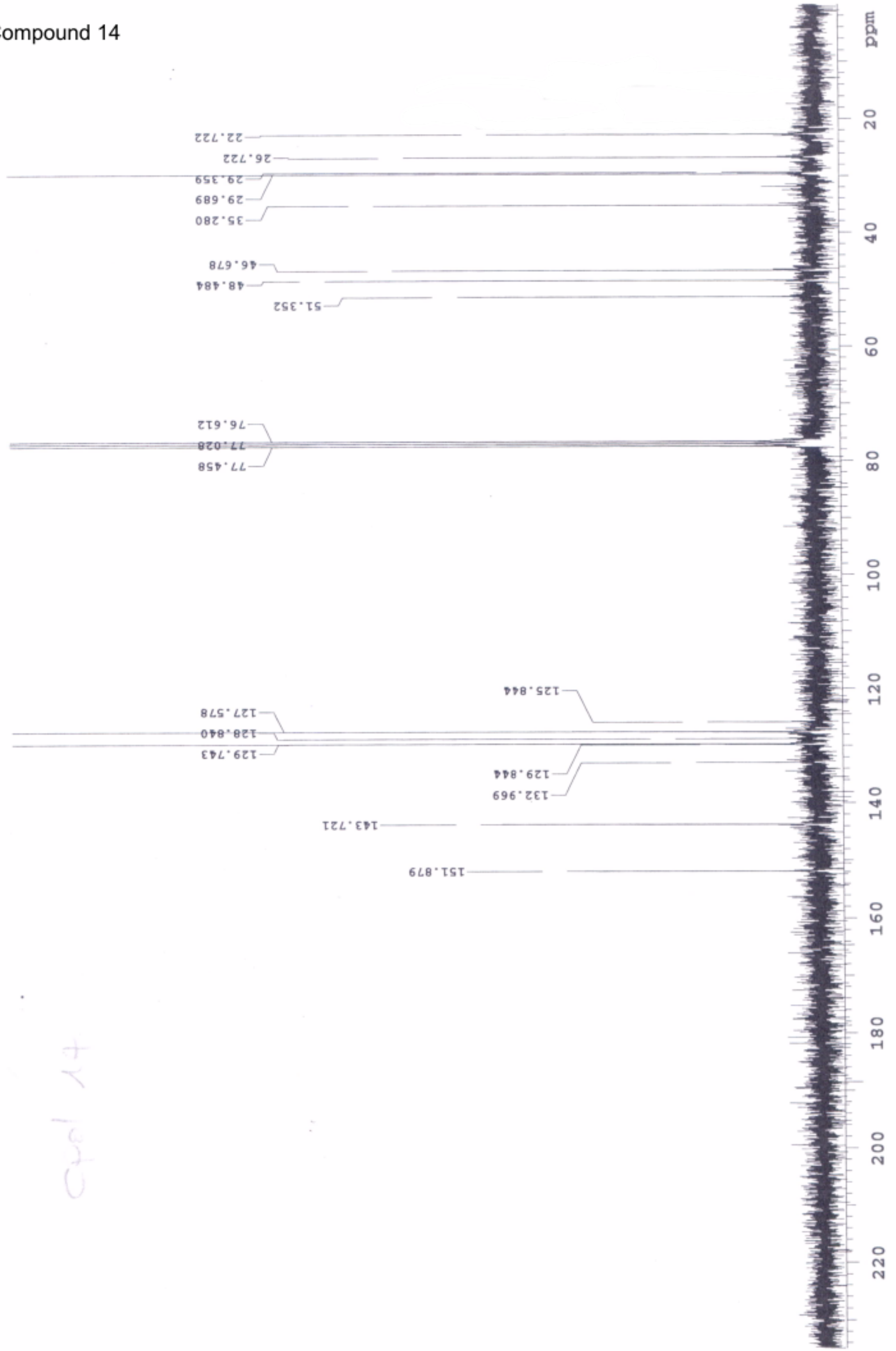
22.722
26.722
29.359
29.689
35.280
46.678
48.484
51.352
76.612
77.028
77.458
125.844
127.578
128.840
129.743
129.844
132.969
143.721
151.879
ppm
20
40
60
80
100
120
140
160
180
200
220

Compound 15

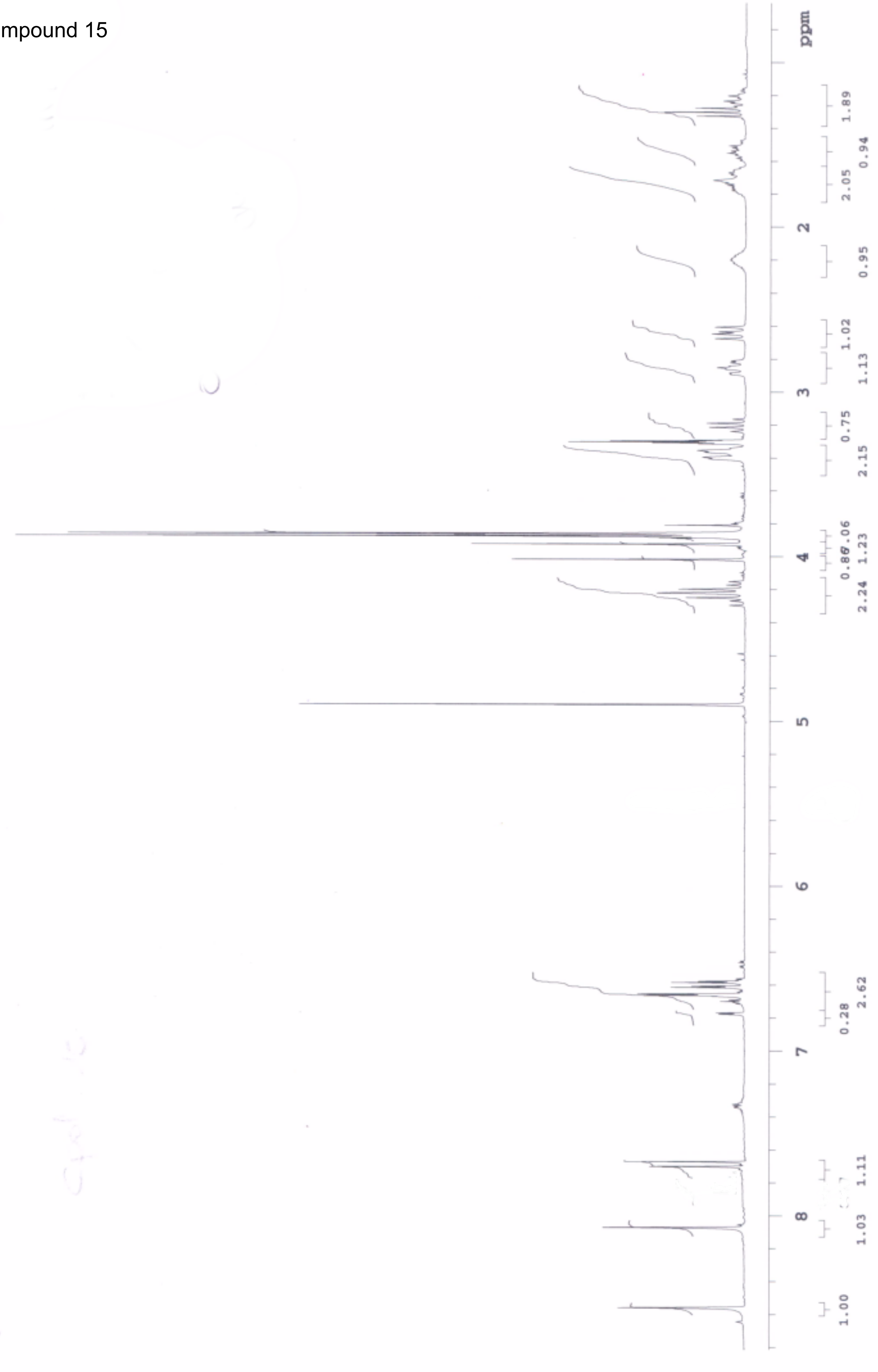
ppm
2
3
4
5
6
7
8
1.89
0.94
2.05
0.95
1.02
1.13
0.75
2.15
1.23
2.24
0.28
2.62
1.11
1.03
1.00

Compound 15

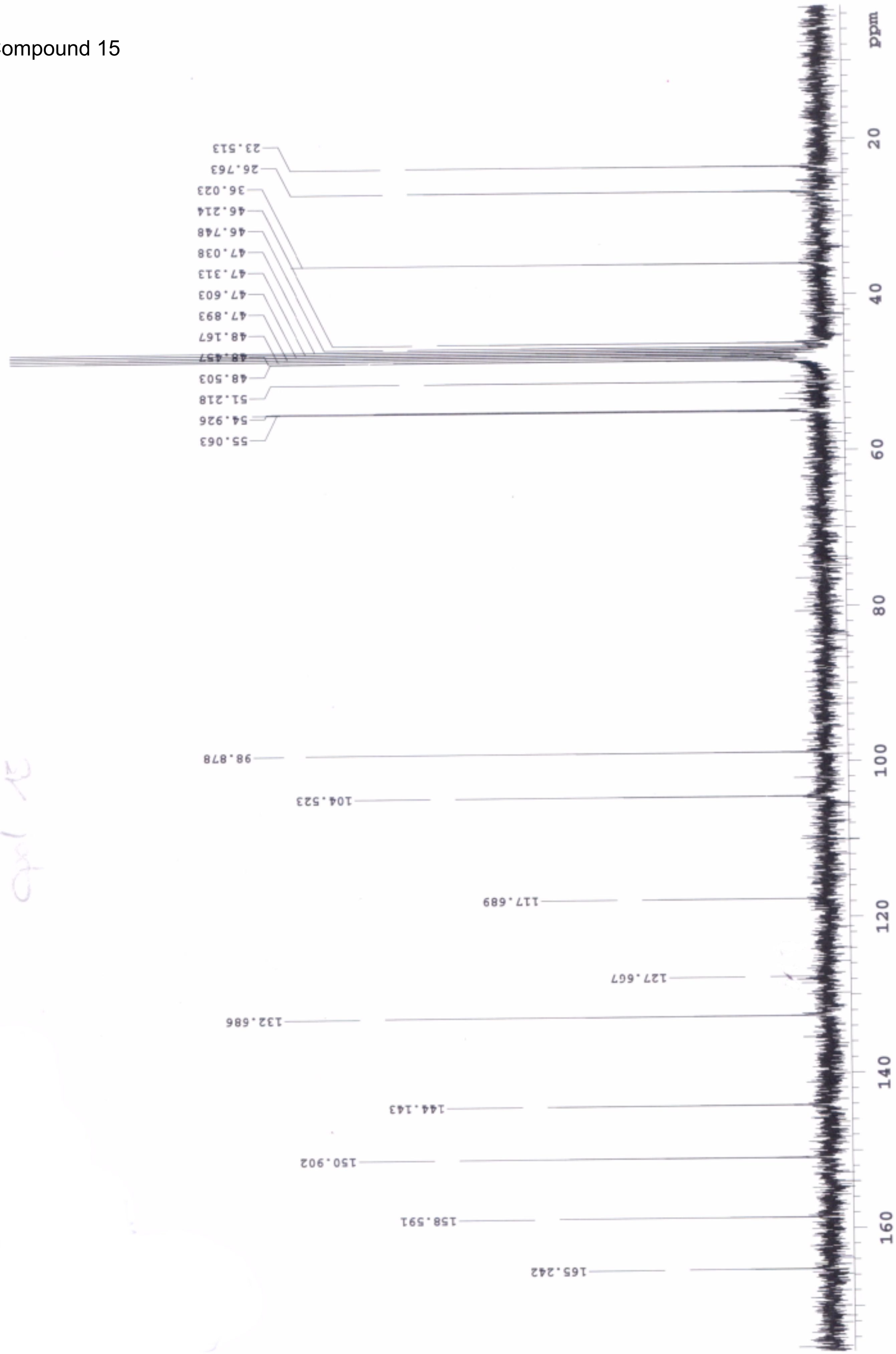

23.513
26.763
36.023
46.214
46.748
47.038
47.313
47.603
47.893
48.167
48.457
48.503
51.218
54.926
55.063
98.878
104.523
117.689
127.667
132.686
144.143
150.902
158.591
165.242
ppm
20
40
60
80
100
120
140
160

Compound 16

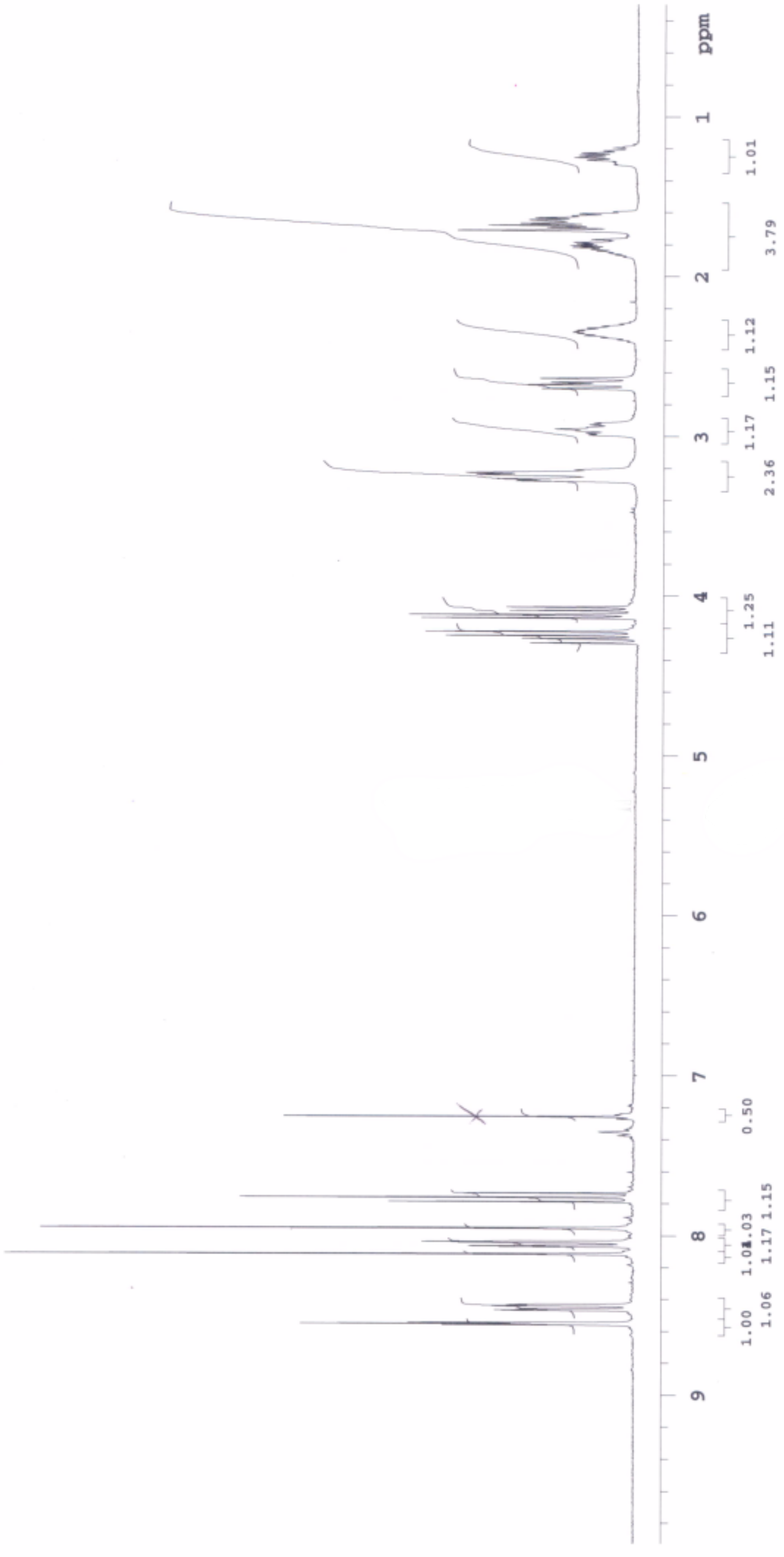

ppm
1
2
3
4
5
6
7
8
9
1.01
3.79
1.12
1.15
1.17
2.36
1.25
1.11
0.50
1.15
1.03
1.17
1.06
1.00

Compound 16

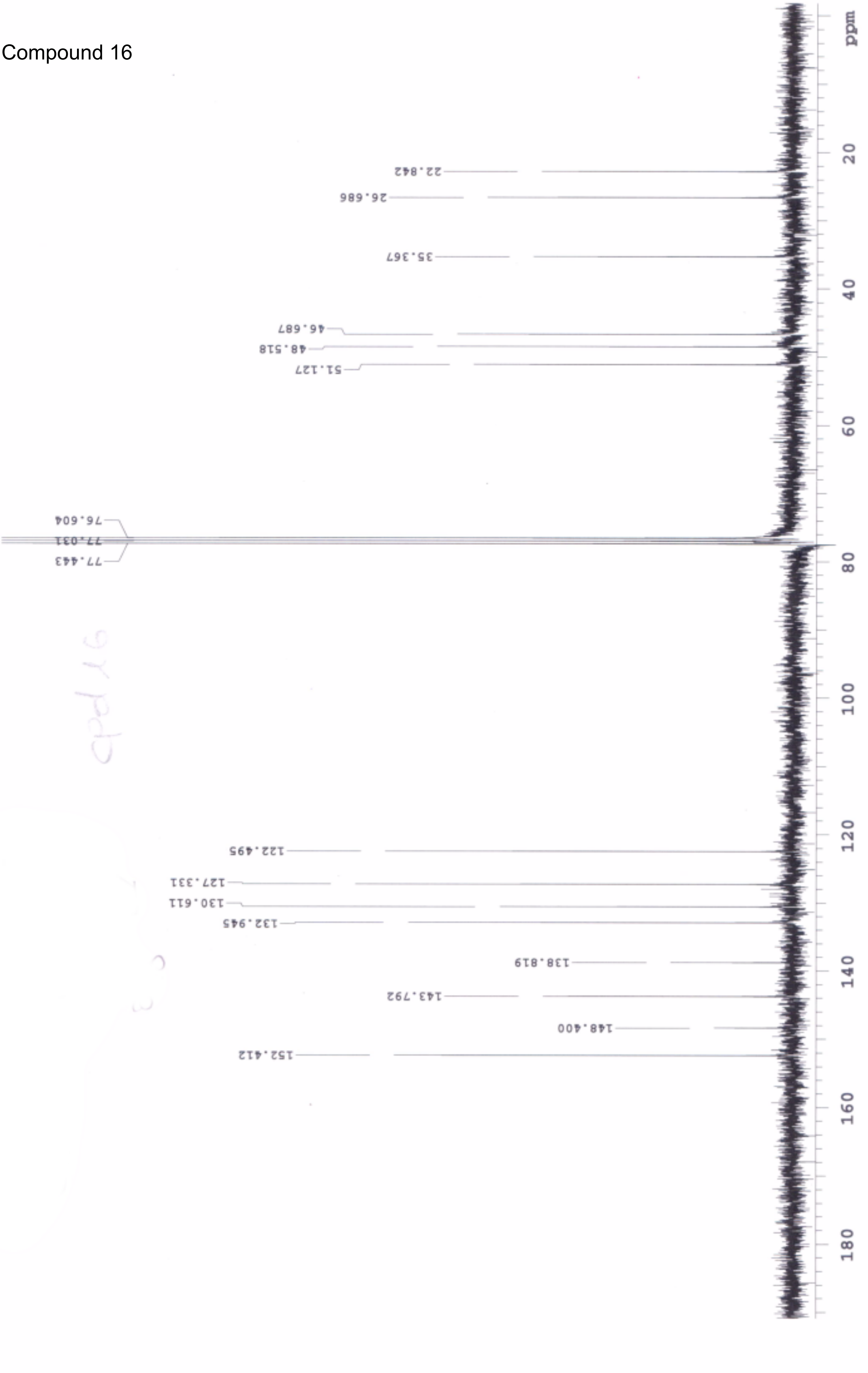
22.842
26.686
35.367
46.687
48.518
51.127
76.604
77.031
77.443
122.495
127.331
130.611
132.945
138.819
143.792
148.400
152.412
ppm
20
40
60
80
100
120
140
160
180
cpd 16

Compound 17

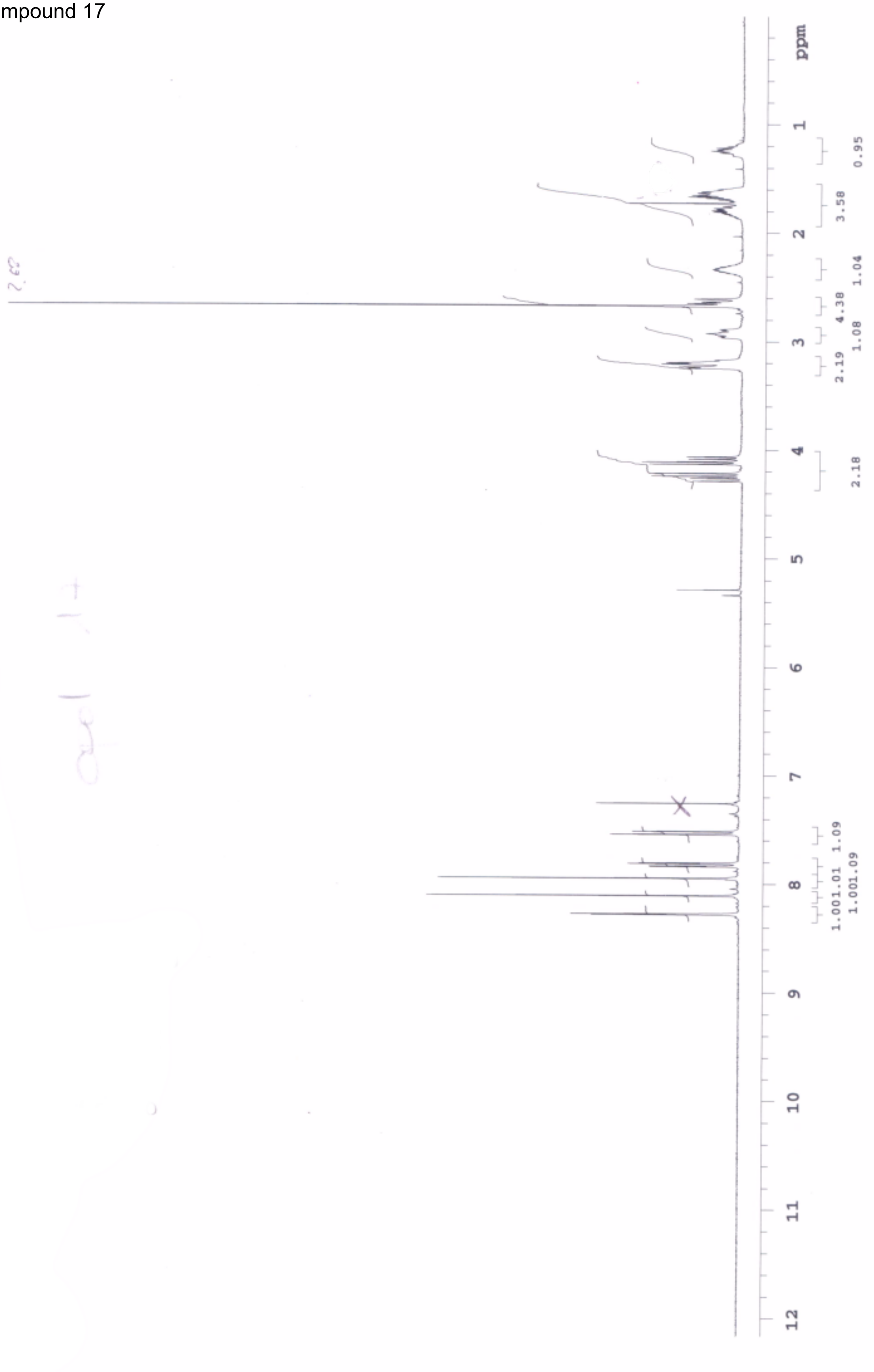
ppm
1
2
3
4
5
6
7
8
9
10
11
12
0.95
3.58
1.04
4.38
1.08
2.19
2.18
1.09
1.001.01
1.001.09

Compound 17

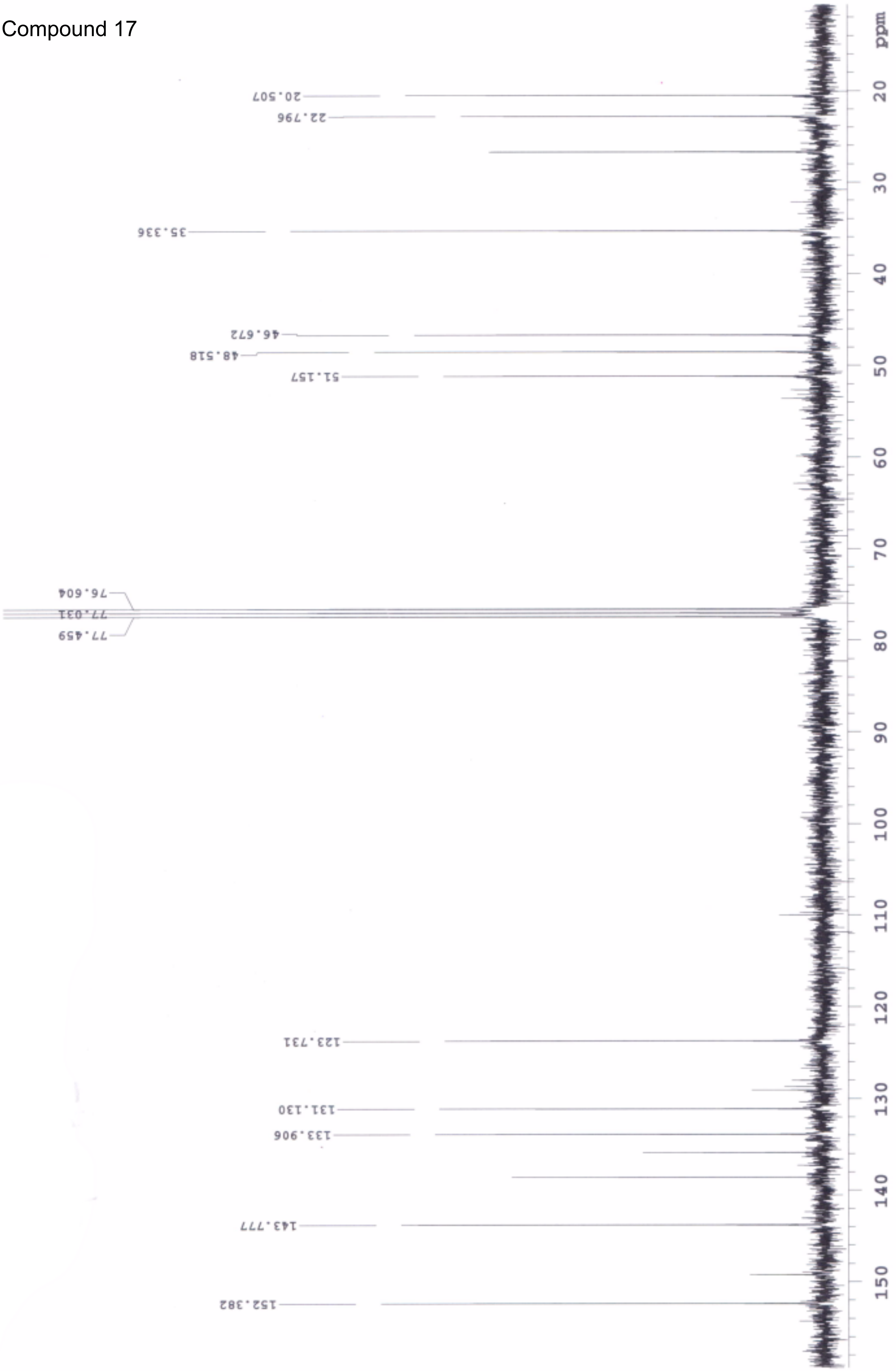
20.507
22.796
35.336
46.672
48.518
51.157
76.604
77.031
77.459
123.731
131.130
133.906
143.777
152.382
ppm
20
30
40
50
60
70
80
90
100
110
120
130
140
150

Compound 18

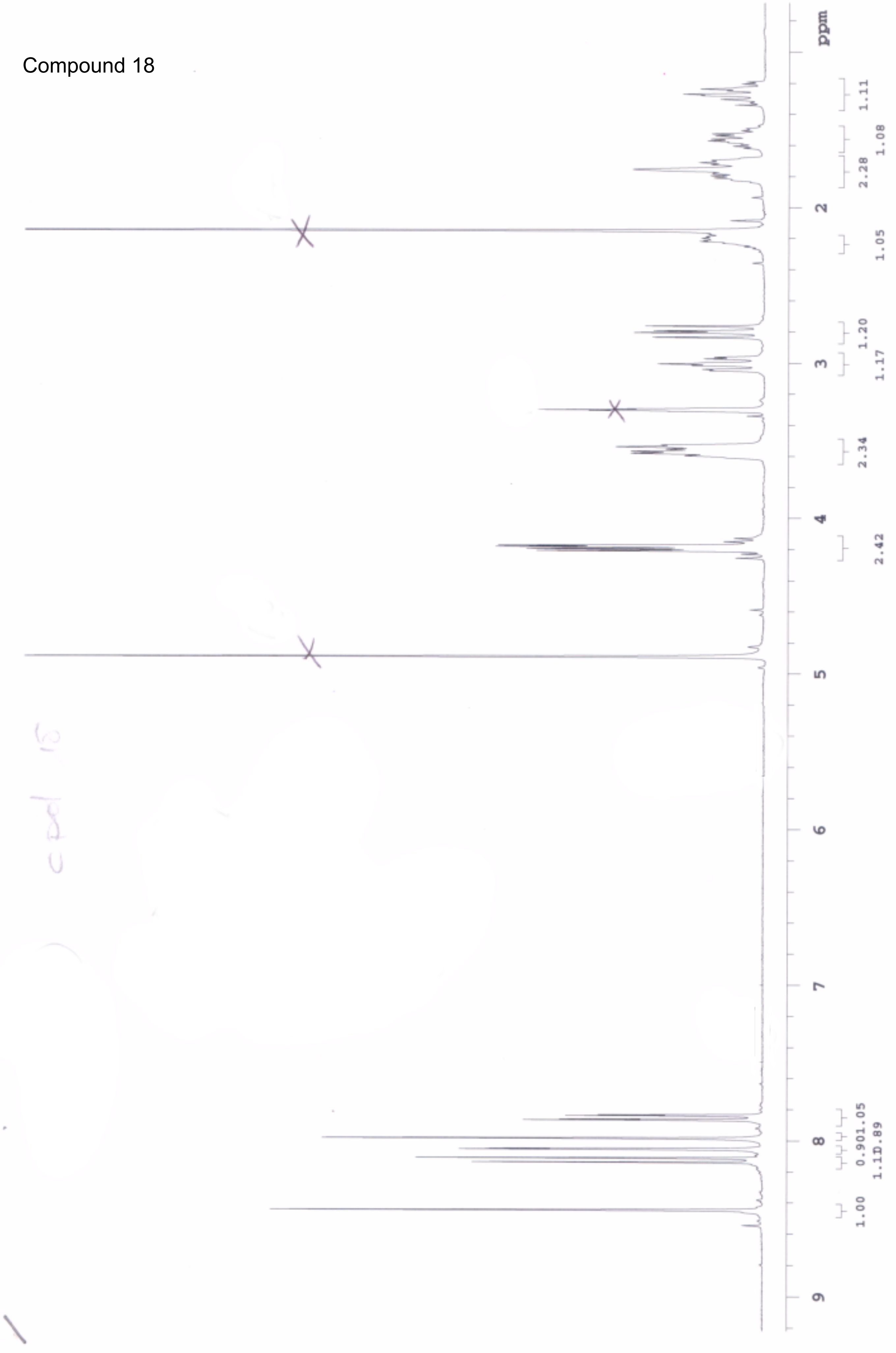

Compound 18

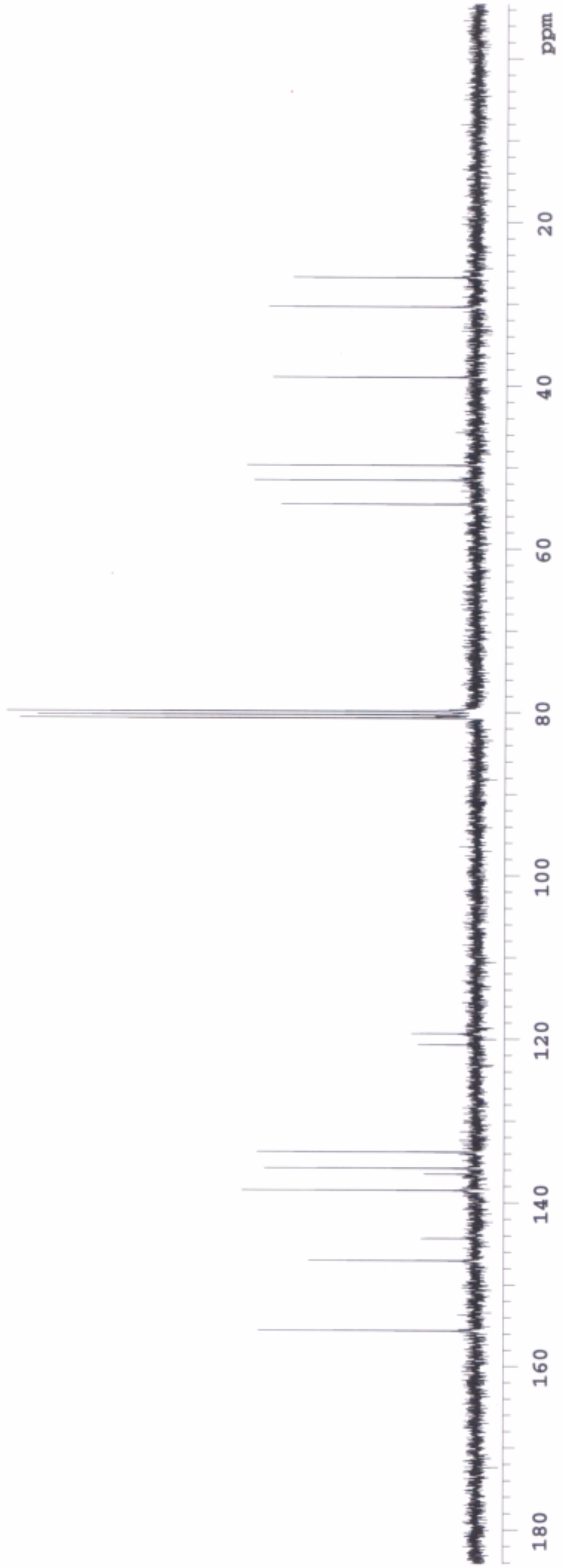

Compound 19

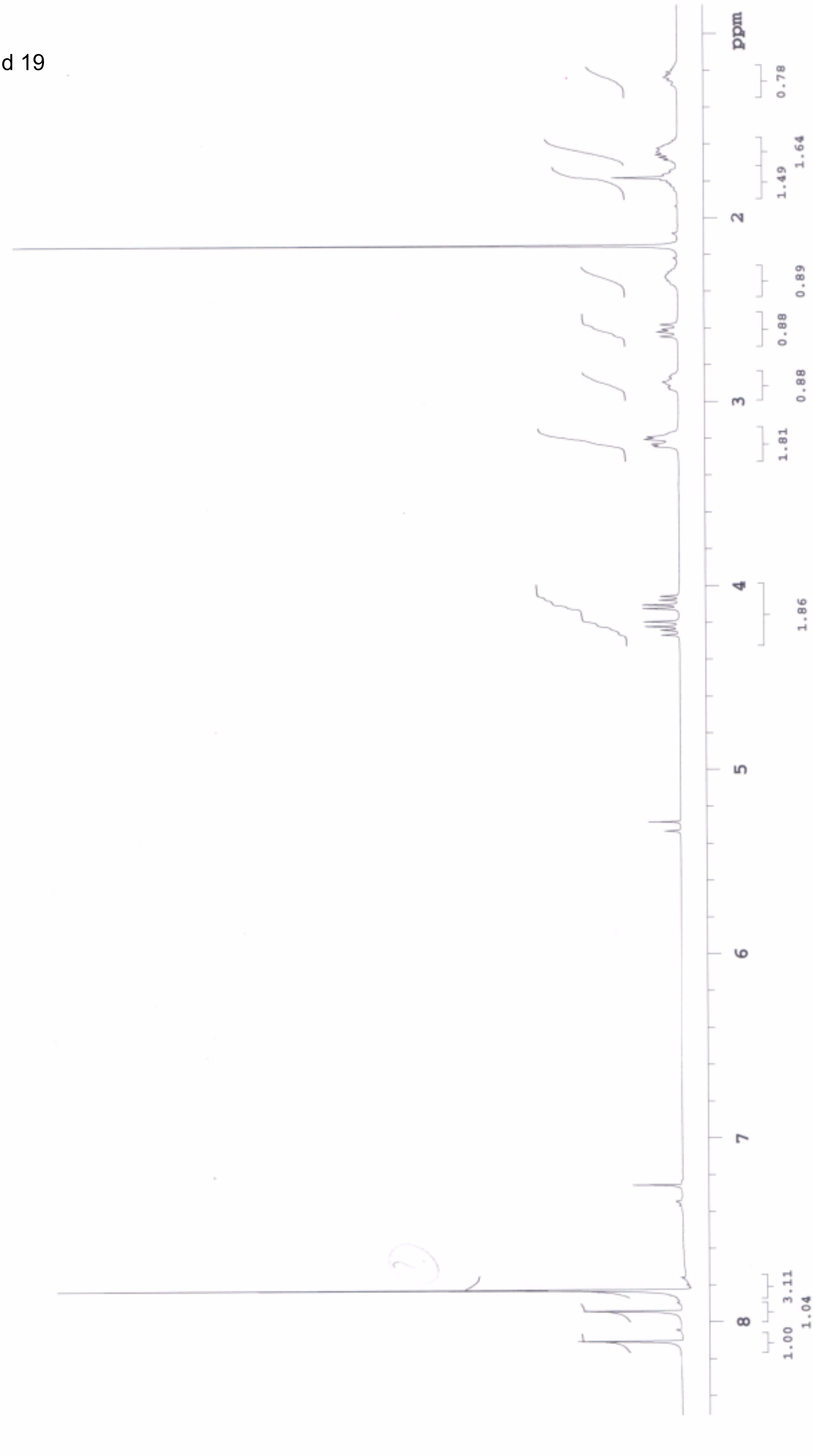

Compound 19

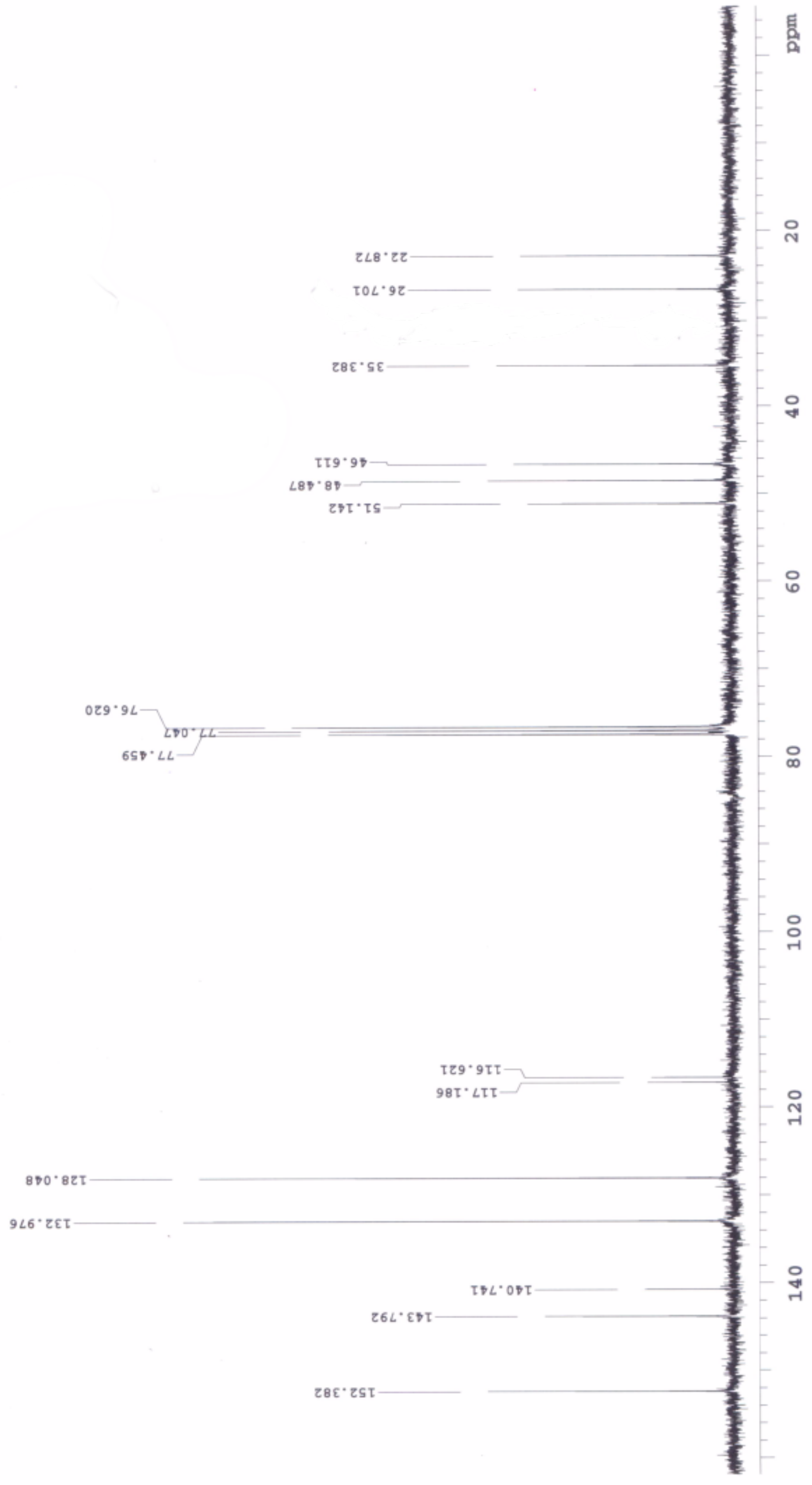

ppm
20
40
60
80
100
120
140
22.872
26.701
35.382
46.611
48.487
51.142
76.620
77.047
77.459
116.621
117.186
128.048
132.976
140.741
143.792
152.382

Compound 20

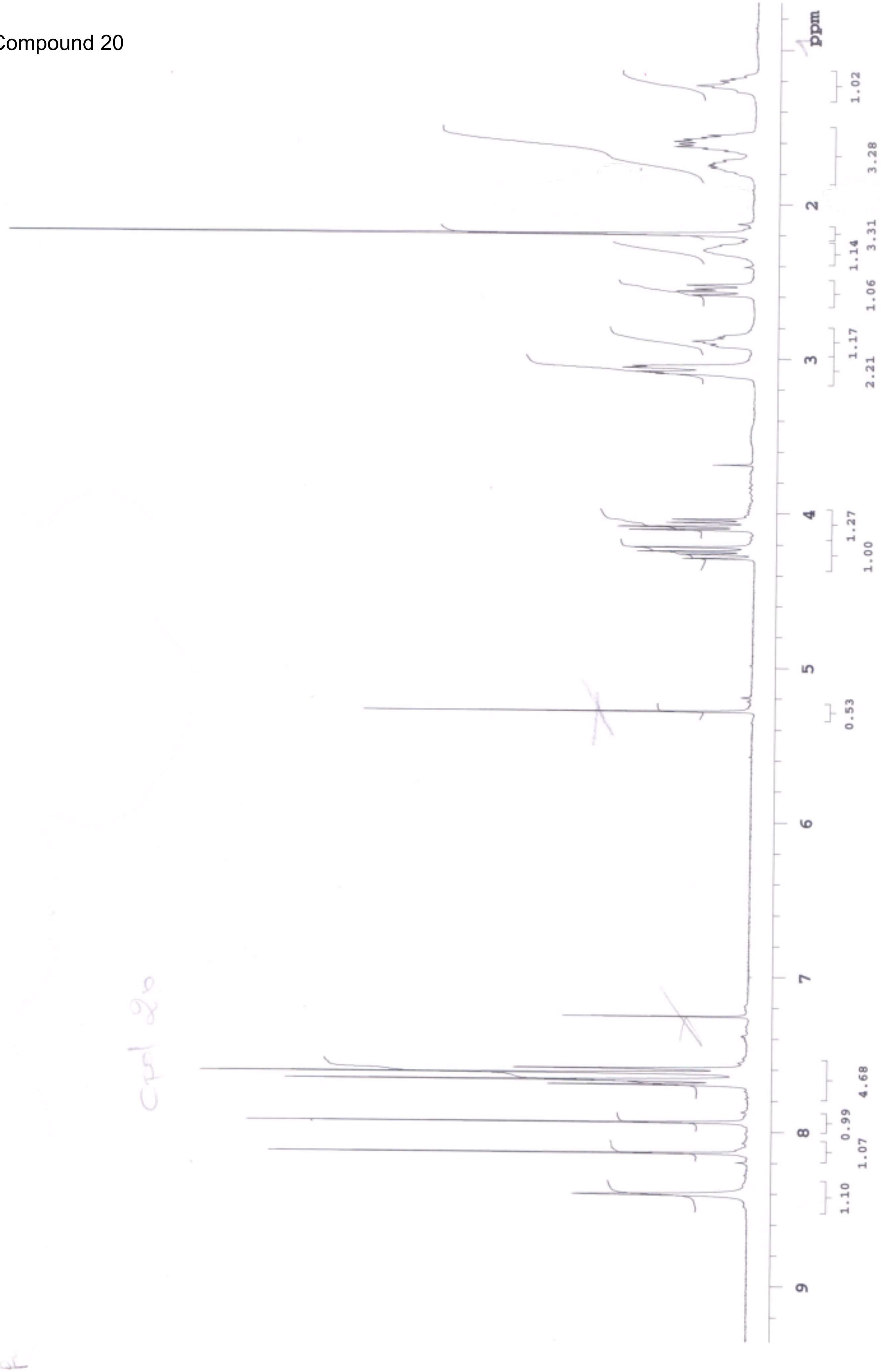
ppm
2
3
4
5
6
7
8
9
1.02
3.28
1.14
3.31
1.06
1.17
2.21
1.27
1.00
0.53
4.68
0.99
1.07
1.10

Compound 20

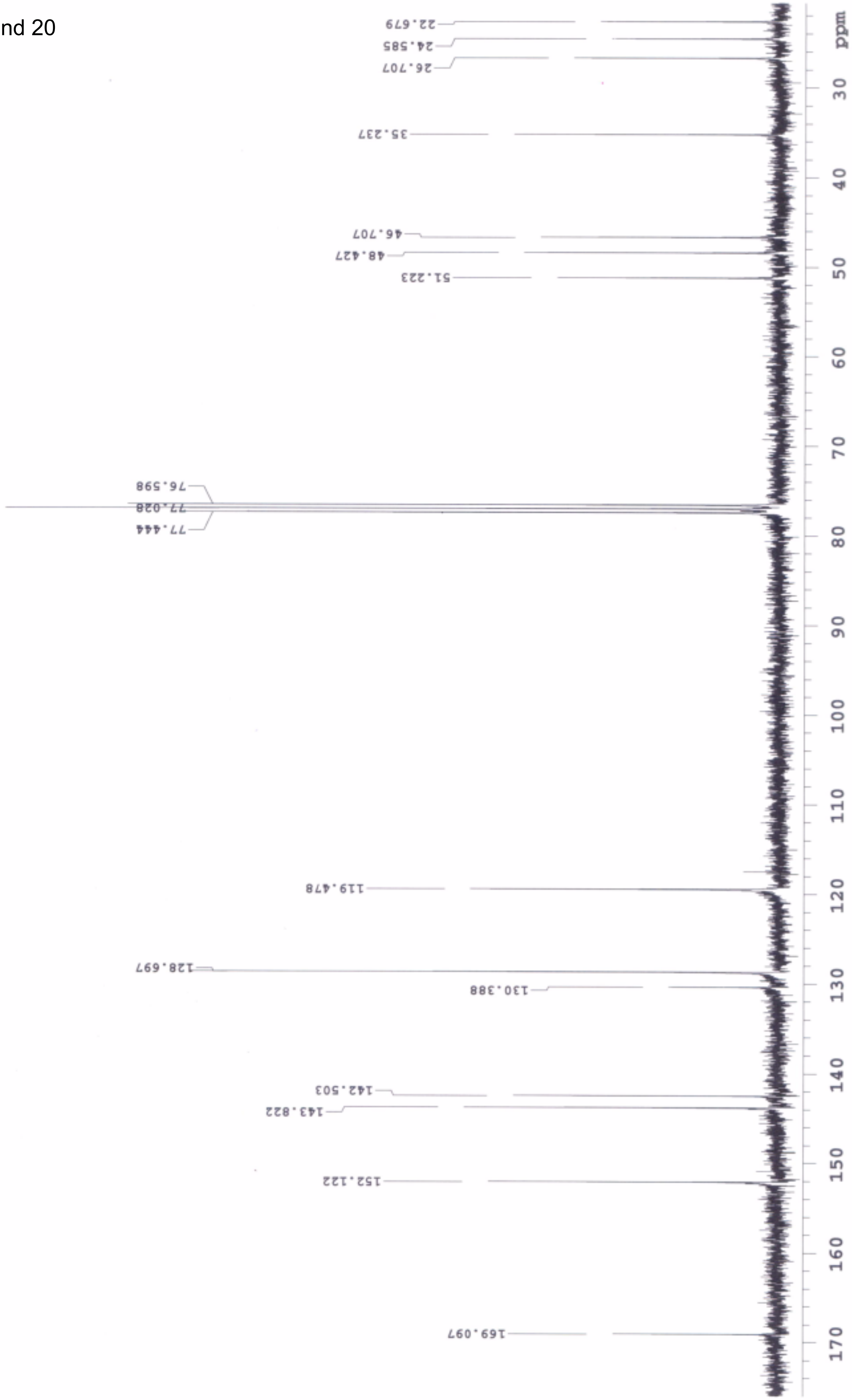
22.679
24.585
26.707
35.237
46.707
48.427
51.223
76.598
77.028
77.444
119.478
128.697
130.388
142.503
143.822
152.122
169.097
ppm
30
40
50
60
70
80
90
100
110
120
130
140
150
160
170
